# The power of the smallest: The inhibitory activity of microbial volatile organic compounds against phytopathogens

**DOI:** 10.3389/fmicb.2022.951130

**Published:** 2023-01-04

**Authors:** Octávio Augusto Costa Almeida, Natália Oliveira de Araujo, Bruno Henrique Silva Dias, Carla de Sant’Anna Freitas, Luciane Fender Coerini, Choong-Min Ryu, Juliana Velasco de Castro Oliveira

**Affiliations:** ^1^Brazilian Biorenewables National Laboratory (LNBR), Brazilian Center for Research in Energy and Materials (CNPEM), Campinas, Brazil; ^2^Graduate Program in Genetics and Molecular Biology, Institute of Biology, University of Campinas (UNICAMP), Campinas, Brazil; ^3^Molecular Phytobacteriology Laboratory, Korea Research Institute of Bioscience & Biotechnology (KRIBB), Daejeon, South Korea; ^4^Biosystems and Bioengineering Program, University of Science and Technology, Daejeon, South Korea

**Keywords:** microbial volatile organic compounds, sustainability, phytopathogens, bioactive compounds, biotechnology, biological control

## Abstract

Plant diseases caused by phytopathogens result in huge economic losses in agriculture. In addition, the use of chemical products to control such diseases causes many problems to the environment and to human health. However, some bacteria and fungi have a mutualistic relationship with plants in nature, mainly exchanging nutrients and protection. Thus, exploring those beneficial microorganisms has been an interesting and promising alternative for mitigating the use of agrochemicals and, consequently, achieving a more sustainable agriculture. Microorganisms are able to produce and excrete several metabolites, but volatile organic compounds (VOCs) have huge biotechnology potential. Microbial VOCs are small molecules from different chemical classes, such as alkenes, alcohols, ketones, organic acids, terpenes, benzenoids and pyrazines. Interestingly, volatilomes are species-specific and also change according to microbial growth conditions. The interaction of VOCs with other organisms, such as plants, insects, and other bacteria and fungi, can cause a wide range of effects. In this review, we show that a large variety of plant pathogens are inhibited by microbial VOCs with a focus on the *in vitro* and *in vivo* inhibition of phytopathogens of greater scientific and economic importance in agriculture, such as *Ralstonia solanacearum*, *Botrytis cinerea*, *Xanthomonas* and *Fusarium* species. In this scenario, some genera of VOC-producing microorganisms stand out as antagonists, including *Bacillus*, *Pseudomonas*, *Serratia* and *Streptomyces*. We also highlight the known molecular and physiological mechanisms by which VOCs inhibit the growth of phytopathogens. Microbial VOCs can provoke many changes in these microorganisms, such as vacuolization, fungal hyphal rupture, loss of intracellular components, regulation of metabolism and pathogenicity genes, plus the expression of proteins important in the host response. Furthermore, we demonstrate that there are aspects to investigate by discussing questions that are still not very clear in this research area, especially those that are essential for the future use of such beneficial microorganisms as biocontrol products in field crops. Therefore, we bring to light the great biotechnological potential of VOCs to help make agriculture more sustainable.

## Introduction

World population growth is expected to reach almost 10 billion people by 2050 and one of the major concerns relies on the food insecurity that may accompany it (United Nations, 2022). The issue becomes even more complex since the most important crops (e.g., rice, wheat, tomato, potato, banana, citrus and apple) are strongly affected by bacterial and fungal pathogens. They infect crops at the early stages of plant growth and remain until the handling of the food product in the market or industry. For instance, postharvest diseases have been estimated to be responsible for 30–50% of crop losses in developing countries (Fakruddin et al., 2015; Kumar and Kalita, 2017). Of the 7,100 classified bacterial species, approximately 150 cause serious losses in many different crops throughout the entire world, being more frequent in tropical and subtropical countries (Kannan et al., 2015). These microorganisms, known as phytopathogenic bacteria, provoke symptoms such as spots, blights, cankers and tissue rots (Martins et al., 2018). The genera most often associated with plant disease are *Clavibacter, Xanthomonas, Erwinia*, *Pectobacterium*, *Pantoea*, *Agrobacterium*, *Pseudomonas*, *Ralstonia*, *Burkholderia*, *Acidovorax*, *Streptomyces*, *Xylella*, *Spiroplasma*, and *Phytoplasma* (Kannan et al., 2015). On the other hand, most fungal pathogen species belong to the phyla Ascomycota and Basidiomycota. Among ascomycetes, fungal plant pathogens belong to various classes, such as Sordariomycetes (e.g., *Fusarium* sp.), Dothideomycetes (e.g., *Phaeosphaeria* sp., *Alternaria* sp.) or Leotiomycetes (e.g., *Botrytis* sp.*, Monilinia* sp., *Sclerotium* sp.). Basidiomycetes are represented by two large and important plant pathogen groups: rusts (Pucciniomycetes) and smuts (Ustilaginomycetes) (Doehlemann et al., 2017). Fungi have a wide spectrum of lifestyles and high genetic plasticity, which allow them to colonize new hosts, develop resistance to fungicides and break the resistance trait created by breeding programs (Doehlemann et al., 2017). In addition, Oomycetes (a phylogenetic lineage of fungus-like eukaryotic microorganisms) include some of the most important plant pathogens, which can cause seedling blights, damping-off, root rots, downy mildews and various other diseases (Kamoun et al., 2015). Consequently, new biotechnological strategies are also needed to reduce their damage to crops.

In this scenario, microorganisms have vast biotechnological potential to meet global agricultural demands in a sustainable way (Singh et al., 2020). One of these demands is to control plant diseases that are responsible for the huge losses in crop and ornamental plant production (Green et al., 2020; Thakur et al., 2020). Thus, scientists have sought to understand, reproduce and enhance the protective effects that beneficial microbes might offer to plants in nature. For this purpose, microbial volatile organic compounds (VOCs), a blend of small signaling molecules produced by microorganisms, are important bioactive compounds against plant pathogens (Mitchell et al., 2010; Munjal et al., 2016; Raza et al., 2016a; Guevara-Avendaño et al., 2019). Each microorganism produces a wide and unique range of VOCs, also called volatilome, encompassing compounds of several chemical classes, and its production may also vary according to the microbial growth conditions.

In the last two decades, several studies have reported the ability of bacteria and fungi to inhibit the growth of phytopathogens through VOC emission. Part of the above-mentioned phytopathogens have already been successfully inhibited by microbial antagonists *in vitro*. Interestingly, some of the studies have demonstrated the antimicrobial activity *in vivo* as well, controlling diseases in grapes, wheat, peaches, and strawberries, for instance. Bacteria such as *Bacillus*, *Pseudomonas* and *Serratia*, and fungi, such as *Aureobasidium* and *Candida*, stood out as potential biological control agents. Importantly, several VOCs produced by these antagonists have been validated, including alcohols (e.g., 2-phenylethanol and 3-methyl-1-butanol), ketones (2-nonanone and 2-heptanone), pyrazines (2,5-dimethyl pyrazine and 2-methyl pyrazine) and sulfur-containing compounds (dimethyl disulfide and dimethyl trisulfide). Such diversity of molecules reflects on the very different molecular mechanisms involved in the pathogen growth inhibition discovered so far. For instance, it was showed that VOCs from *Pseudomonas* sp. caused DNA damaged to the sugarcane pathogen *Thielaviopsis ethacetica* (Freitas et al., 2022). Structural damage on hyphae and down-regulation in the expression of virulence factors in *Ralstonia solanacearum* are other mechanisms by which VOCs act (Raza et al., 2016a,b,c).

Thus, taking advantage on the effects of VOCs on phytopathogens, these bioactive compounds can be a safe biotechnological alternative to reduce the use of chemicals in agriculture and increase productivity. Interestingly, microbial VOC producers do not need to colonize the roots or the rhizosphere once VOCs can permeate through soil and then interact with plants and other microorganisms at a certain distance (Holopainen and Blande, 2012; Schulz-Bohm et al., 2018). However, important issues still rely on the production of a bioproduct that can effectively provide plant protection under open-field conditions, but also being economically viable and safe to human and environmental health (Korpi et al., 2009; Tilocca et al., 2020).

**Figure 1:**
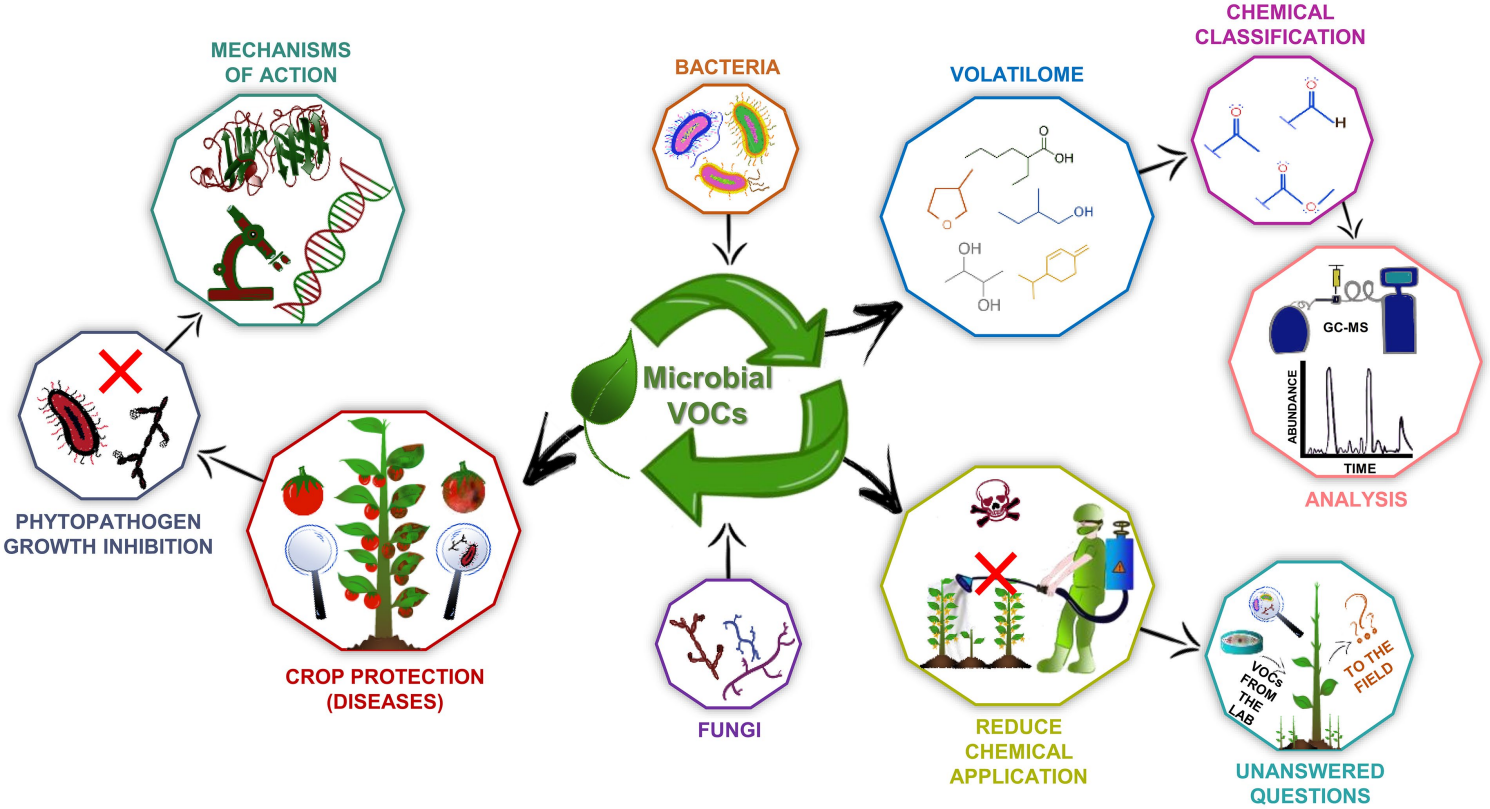
Biotechnological aspects of microbial volatile organic compounds (VOCs). They are produced by fungi and bacteria and belong to diverse chemical classes that can be identified by several techniques, such as gas chromatography coupled to mass spectrometry (GC–MS). These molecules have great biotechnology potential as plant growth promoters and phytopathogen inhibitors, and many studies have demonstrated such effects *in vitro* and *in vivo*. Therefore, it has become possible to develop bioproducts to be applied in the field and thereby reduce the use of agrochemicals. Nevertheless, some questions about the use of microbial VOCs as biotechnological products need to be answered. Moreover, although several beneficial microorganisms and bioactive VOCs have been described, the molecular mechanisms of action of these compounds remain poorly understood.

Herein, we approach the main aspects of microbial VOCs able to inhibit phytopathogens (Figure 1). First, it will describe the main characteristics about the volatilomes and its dynamicity. Sequentially, we highlight the biotechnological potential of these molecules in inhibiting some of the main economically important plant pathogens, as diverse studies have reported through *in vitro* and *in vivo* assays. Then, the mechanisms by which VOCs directly affect pathogens are addressed. Finally, similar to any innovation, there are several questions and challenges to overcome in the future, which are discussed in the last section.

## Microbial VOCs at a glance

Microbial VOCs are mostly carbon-based, small signaling molecules that evaporate easily at room temperature (Schulz and Dickschat, 2007; Korpi et al., 2009; Bitas et al., 2013; Kanchiswamy et al., 2015), and are able to move long distances through elements such as water, air and soil while in a gaseous state (Kanchiswamy et al., 2015). These compounds are mostly lipophilic, have low molecular weight (<300 g․mol^−1^), are derived from several biosynthetic pathways, and have high vapor pressures (0.01 KPa) at a temperature of 20°C (Bitas et al., 2013; Kanchiswamy et al., 2015). Generally, microbial VOCs are synthesized as side products during both primary and secondary metabolism (Schulz and Dickschat, 2007; Korpi et al., 2009).

Fungi and bacteria are capable of releasing a wide range of VOCs into the environment (Stahl and Parkin, 1996; Schulz and Dickschat, 2007). The predominant chemical classes of microbial VOCs are alkenes, alcohols, ketones, terpenes, benzenoids, pyrazines, acids, esters and aldehydes (Schulz and Dickschat, 2007; Korpi et al., 2009; Wenke et al., 2012; Peñuelas et al., 2014; Li et al., 2016), although the percentage of compounds in each class varies between bacteria and fungi (Peñuelas et al., 2014). For instance, bacteria emit a greater variety of terpenes, alkenes and ketones than fungi, whereas fungal VOCs emit a higher range of aldehydes, benzenoids and alcohols. Fungi also emit arsenics, chlorides, nitriles, thiofurans, alkynes, bromides and tellurium compounds, which are not commonly identified in bacterial volatilomes (Peñuelas et al., 2014).

It is well known that microbial volatilomes also vary according to the producing microorganism, showing some similarities among microbes belonging to the same genus (Choudoir et al., 2019; Guo et al., 2019b) or even the same species (Macías-Rubalcava et al., 2018; Guevara-Avendaño et al., 2019), but also some uniqueness by each one. Those conditions can be media composition (Fiddaman and Rossall, 1994; Bruce et al., 2004; Raza et al., 2015; Asari et al., 2016; Huang et al., 2018; Morita et al., 2019), media consistency (Dickschat et al., 2005; Qadri et al., 2015), period of incubation/growth stage (Weise et al., 2012; Giorgio et al., 2015; Sánchez-Fernández et al., 2016; Macías-Rubalcava et al., 2018; Misztal et al., 2018; Yang et al., 2019), humidity/water content (Jeleń, 2002; Misztal et al., 2018), temperature (Jeleń, 2002), oxygen and carbon dioxide levels (Zhang et al., 2013a), and interactions with other organisms (Sánchez-Fernández et al., 2016; Delgado et al., 2021).

Such dynamicity and diversity of microbial VOCs can influence complex environmental trophic interactions (Fiedler et al., 2001; Bitas et al., 2013). In this regard, microbial VOCs play an essential function as info-chemicals in microbial interactions by inducing or repressing the development and behavior of a wide range of organisms such as other microorganisms, plants, and animals (Bitas et al., 2013; Chaparro et al., 2014; Kanchiswamy et al., 2015). In plant-microbe interactions, microbial VOCs drives different responses in plant fitness and physiology, resulting in growth promotion, induced systemic resistance, tolerance against abiotic stress and microbiome shifting (Ryu et al., 2003; Vaishnav et al., 2015; Sharifi and Ryu, 2016; Dias et al., 2021; Kong et al., 2021). Regarding microbe-animal interactions, fungal and bacterial volatiles can also act as allelochemicals, triggering olfactory responses in insects and, consequently, influencing their behavior (Thakeow et al., 2008; Kandasamy et al., 2019; Goelen et al., 2020), which might also be applied to protect forest and crop production against these predators. Moreover, these molecules can enhance or inhibit the growth of other surrounding microorganisms. The interesting and efficient VOC-mediated antimicrobial activity against economically important phytopathogens will be explored in the upcoming sections.

## Microbial VOCs inhibit a wide range of economically important phytopathogens

In the last two decades, studies have demonstrated that inhibition of phytopathogens can also be achieved through the release of VOCs by microorganisms. Thus, this section will provide an overview of fungal and bacterial species that were inhibited *in vitro* and *in vivo* by VOCs, with a focus on the main species that cause damage to agriculture and their antagonists. Table 1 brings those and more phytopathogens reported in literature and their respective antagonists. Furthermore, some specific VOCs were already validated as the molecules with the inhibitory activity. In order to validate them, the volatilomes produced by the microorganisms are first identified using different techniques (Morita et al., 2019; Guo et al., 2019b; Delgado et al., 2021), and then the compounds are purified using chromatography techniques or purchased in their synthetic form, to be tested *in vitro* against pathogens (Vinale et al., 2008; Munjal et al., 2016; Raza et al., 2016a, 2016b; Gotor-Vila et al., 2017). Due to some reasons that will be discussed later, it is not trivial to reproduce similar effects observed in the tests with the microbial antagonist by using synthetic compounds instead; however, several molecules with inhibitory activity have been validated thus far, which can be checked on Supplementary Table S1. Interestingly, some microbes and specific VOCs have already been validated in plants as well as in postharvest commodities (e.g., cherries, peaches, strawberries, tomatoes), which reinforces their biotechnological potential. We will also show that microbial VOCs can control numerous diseases affecting different parts of the plants (roots, stems, leaves, and fruits), thus improving their performance and productivity. Although some of these *in vivo* assays were performed with the microbial antagonist in physical contact with the phytopathogens (where other diffusible metabolites could be acting), the role of the VOCs in the growth inhibition was previously validated *in vitro*.

**Table 1:** Phytopathogens inhibited (*in vitro* and/or *in vivo*) by potential biocontrol agents.

Phytopathogens	Inhibited by	References
*Agrobacterium tumefaciens*	*Pseudomonas chlororaphis*, *Pseudomonas fluorescens*, S*erratia plymuthica, Serratia proteamaculans*	Plyuta et al., 2016
	*Ps. chlororaphis*, *Ps. fluorescens*, *Se. plymuthica, Se. proteamaculans*	Popova et al., 2014
	*Pseudomonas putida*, *Se. plymuthica*, *Burkholderia phytofirmans*	Chernin et al., 2013
	*Ps. fluorescens, Se. plymuthica*	Dandurishvili et al., 2011
*Agrobacterium vitis*	*Ps. fluorescens*, *Se. plymuthica*	Dandurishvili et al., 2011
	*Pseudomonas putida*, *Se. plymuthica*, *Burkholderia phytofirmans*	Chernin et al., 2013
*Alternaria alternata*	*Aureobasidium pullulans*	Yalage Don et al., 2020
	*Bacillus pumillus*	Morita et al., 2019
	*Bacillus subtilis*	Chaurasia et al., 2005
	*B. subtilis*	Gao et al., 2018
	*Bacillus velezensis*	Myo et al., 2019
	*Burkholderia ambifaria*	Groenhagen et al., 2013
*Alternaria brassicae*	*Bacillus amyloliquefaciens*	Asari et al., 2016
	*Ba. pumillus*, *Ba. subtilis*, *Paenibacillus polymyxa*	Liu et al., 2008
*Alternaria brassicola*	*Ba. amyloliquefaciens*	Asari et al., 2016
*Alternaria helianthi*	*Muscodor crispans*	Mitchell et al., 2010
*Alternaria solani*	*Muscodor yucatenensis*	Macías-Rubalcava et al., 2010
	*Ba. pumillus*, *Ba. subtilis*, *Pa. polymyxa*	Liu et al., 2008
	*Nodulisporium* sp.	Sánchez-Fernández et al., 2016
*Ascochyta citrullina*	*Ba. pumillus*, *Ba. subtilis*, *Pa. polymyxa*	Liu et al., 2008
*Aspergillus carbonarius*	*Cyberlindnera jadinii*	Farbo et al., 2018
*Aspergillus flavus*	*Diaporthe phaseolarum*	Qadri et al., 2015
	*Wickerhamomyces anomalus*	Hua et al., 2014
*Aspergillus niger*	*Arthrobacter* sp., *Pseudoalteromonas* sp.	Papaleo et al., 2012
	*Ba. pumillus*	Morita et al., 2019
	*Lysinibacillus* sp.	Che et al., 2017
	*Serratia odorifera*, *Se. plymuthica*, *Stenotrophomonas maltophilia*, *Stenotrophomonas rhizophila*	Vespermann et al., 2007
*Aspergillus ochraceus*	*Candida intermedia, Candida friedrichii, Lachancea thermotolerans*	Tilocca et al., 2019
	*Hanseniaspora uvarum, Pichia kluyveri, W. anomalus*	Masoud et al., 2005
*Aspergillus tubingensis*	*Gluconobacter cerinus + Hanseniaspora osmophila* (bioproduct)	Delgado et al., 2021; Besoain Canales et al., 2017
*Athelia rolfsii*	*Bacillus megaterium*	Munjal et al., 2016
	*Burkholderia tropica*	Tenorio-Salgado et al., 2013
	*Pseudomonas putida*	Sheoran et al., 2015
*Aureobasidium pullulans*	*Saccharomyces cerevisiae, Serratia* sp.	Bruce et al., 2004
*Bipolaris sorokiniana*	*Mu. crispans*	Mitchell et al., 2010
*Blumeria graminis*	*Irpex lacteus*	Koitabashi et al., 2002, 2004
*Botrydiplodium thobrone*	*Sa. cerevisiae, Serratia* sp.	Bruce et al., 2004
*Botrytis mali*	*Bacillus cereus*, *Ba. pumillus*, *Ba. subtilis*	Jamalizadeh et al., 2010
*Botrytis cinerea*	*A. pullulans*	Yalage Don et al., 2020
	*A. pullulans*, *Aureobasidium* sp., *Candida stellimacola, Candida tropicalis, Galactomyces candidum*, *Monilliela* sp., *Pichia kudiavzevii, Sa. cerevisiae, Saccharomyces paradoxus*	Chen et al., 2018
	*A. pullulans*	Di Francesco et al., 2015
	*A. pullulans, Metschnikowia pulcherrima, Sa. cerevisiae, Wickerhamomyces cerevisiae*	Parafati et al., 2015
	*Ba. amyloliquefaciens*	Asari et al., 2016
	*Ba. amyloliquefaciens*	Gotor-Vila et al., 2017
	*Ba. amyloliquefaciens*, *Bacillus licheniformis*, *Ba. subtilis*	Chen et al., 2019
	*Bacillus atrophaeus*	Zhang et al., 2013a
	*Ba. subtilis*	Gao et al., 2018
	*Ba. subtilis, Ba. pumillus, Pa. polymyxa*	Liu et al., 2008
	*Ba. velezensis*	Cheffi et al., 2019
	*Ba. velezensis*	Jiang et al., 2018
	*Ba. velezensis*	Myo et al., 2019
	*Candida sake, Pi. kluyveri*	Mewa-Ngongang et al., 2019
	*Ca. sake*	Arrarte et al., 2017
	*G. cerinus + H. osmophila* (bioproduct)	Delgado et al., 2021; Besoain Canales et al., 2017
	*H. uvarum*	Tahir et al., 2017b; Qin et al., 2017
	*Mu. crispans*	Mitchell et al., 2010
	*Pseudomonas stutzeri*	Rojas-Solís et al., 2018
	*Streptomyces mycarofaciens, Streptomyces philanthi*	Boukaew et al., 2017
	*Streptomyces platensis*	Wan et al., 2008
*Burkholderia cepacia*	*Arthrobacter* sp., *Gillisia* sp., *Octadecabacter* sp., *Pseudoalteromonas* sp., *Rhodococcus* sp., *Roseobacter* sp. *Shewanella* sp., *Sphingopyxis* sp.	Papaleo et al., 2012
*Bursaphelenchus xylophilus*	*Bacillus simplex*, *Ba. subtilis*, *Bacillus weihenstephanensis*, *Microbacterium oxydans*, *Serratia marcescens*, *Ste. maltophilia*, *Streptomyces lateritius*	Gu et al., 2007
*Cephalosporium gramineum*	*Mu. crispans*	Mitchell et al., 2010
*Ceratocystis ulmi*	*Mu. crispans*	Mitchell et al., 2010
	*Phoma* sp.	Strobel et al., 2011
*Cercospora beticola*	*Phoma* sp.	Strobel et al., 2011
*Cercospora kikuchii*	*Ba. pumillus, Ba. subtilis*, *Pa. polymyxa*	Liu et al., 2008
*Chaetomium* sp.	*Collimonas pratensis*	Garbeva et al., 2014
*Cladosporium cladosporioides*	*Ba. pumillus*	Morita et al., 2019
	*Ba. velezensis*	Cheffi et al., 2019
	*Streptomyces griseoruber*	Herrington et al., 1987
*Clavibacter michiganensis* subsp. *sepedonicus*	*Ba. subtilis*	Rajer et al., 2017
*Cochliobolus carbonum*	*Mu. crispans*	Mitchell et al., 2010
*Colletotrichum acutatum*	*A. pullulans*	Di Francesco et al., 2015
	*Candida pyralidae, Pi. kluyveri*	Mewa-Ngongang et al., 2019
	*Lysinibacillus* sp.	Che et al., 2017
*Colletotrichum gloeosporioides*	*A. pullulans, G. candidum*	Chen et al., 2018
	*Ba. megaterium*	Munjal et al., 2016
	*Bacillus mycoides, Ba. velezensis*	Guevara-Avendaño et al., 2019
	*Ba. subtilis*	Gao et al., 2018
	*Ba. velezensis*	Cheffi et al., 2019
	*Bu. tropica*	Tenorio-Salgado et al., 2013
	*Debaryomyces nepalensis*	Zhou et al., 2018
	*Ps. putida*	Sheoran et al., 2015
*Colletotrichum lagenarium*	*Mu. crispans*	Mitchell et al., 2010
*Colletotrichum lindemuthianum*	*Ba. amyloliquefaciens*	Martins et al., 2019
	*Ba. velezensis*	Gao et al., 2017
*Colletotrichum* sp.	*Mu. yucatenensis*	Macías-Rubalcava et al., 2010
*Curvularia lunata*	*Ba. pumillus*	Morita et al., 2019
	*Mu. crispans*	Mitchell et al., 2010
*Cylindrocarpon destructans*	*Ba. velezensis*	Cheffi et al., 2019
*Drechslera portulacae*	*Mu. crispans*	Mitchell et al., 2010
*Drechslera teres*	*Mu. crispans*	Mitchell et al., 2010
*Drechslera tritici-repentis*	*Mu. crispans*	Mitchell et al., 2010
*Epicocum nigrum*	*Trichoderma gamsii*	Chen et al., 2016
*Erwinia carotovora*	*Ba. amyloliquefaciens, Ba. subtilis*	Ryu et al., 2004
	*Ps. chlororaphis*	Han et al., 2006
*Exserohilum turcicum*	*Enterobacter aerogenes*	D’Alessandro et al., 2014
*Fulvia fulva*	*Ba. velezensis*	Myo et al., 2019
*Fusarium avenaceum*	*Ba. velezensis*	Cheffi et al., 2019
	*Mu. crispans*	Mitchell et al., 2010
*Fusarium cerealis*	*Ba. velezensis*	Cheffi et al., 2019
*Fusarium culmorum*	*Aspergillus clavatonanicus*	Mishra et al., 2017
	*Ba. subtilis*, *Burkholderia cepacia, Ps. fluorescens*, *Pseudomonas trivialis*, *Se. plymuthica*, *Staphylococcus epidermidis*, *Ste. maltophilia*, *Ste. rhizophila*	Vespermann et al., 2007
	*Burkholderia* sp., *Burkholderia sediminicola, Bu. cepacia, Cellulomonas* sp., *Chryseobacterium indologenes, Collimonas arenae, Collimonas fugivorans, Colli. pratensis, Flavobacterium* sp., *Hydrogenophaga* sp., *Luteibacter* sp., *Lysobacter antibioticus, Matsuebacter chitosanotabidus, Methylobacterium* sp., *Ps. fluorescens, Se. plymuthica, Ste. maltophilia, Streptomyces atratus, Xanthomonas campestris*	Garbeva et al., 2014
	*Bu. tropica*	Tenorio-Salgado et al., 2013
	*Mu. crispans*	Mitchell et al., 2010
	*Pseudomonas donghuensis*	Ossowicki et al., 2017
*Fusarium flocciferum*	*T. gamsii*	Chen et al., 2016
*Fusarium graminearum*	*Aspergillus clavatonanicus*	Mishra et al., 2017
	*Ba. pumillus, Ba. subtilis, Pa. polymyxa*	Liu et al., 2008
	*Ba. velezensis*	Gao et al., 2017; Myo et al., 2019
*Fusarium incarnatum*	*A. pullulans, G. cerevisiae*, *Sa. cerevisiae*	Chen et al., 2018
*Fusarium moniliforme*	*Lysinibacillus* sp.	Che et al., 2017
*Fusarium oxysporum*	*Achromobacter* sp., *Serratia* sp.	Minerdi et al., 2009
	*A. clavatonanicus*	Mishra et al., 2017
	*Ba. amyloliquefaciens*	Yuan et al., 2012
	*Ba. pumillus, Ba. subtilis, Pa. polymyxa*	Liu et al., 2008
	*Ba. pumillus*	Morita et al., 2019
	*Ba. subtilis*	Chaurasia et al., 2005
	*Ba. velezensis*	Gao et al., 2017
	*Ba. velezensis*	Myo et al., 2019
	*Burkholderia gladioli* pv. *agricola*	Elshafie et al., 2012
	*Burkholderia* sp., *B. sediminicola, Cellulomonas* sp., *Ch. indologenes, Colli. arenae, Colli. fugivorans, Colli. pratensis, Hydrogenophaga* sp., *Luteibacter* sp.*, L. antibioticus, Mat. chitosanotabidus, Ps. fluorescens, Se. plymuthica, Ste. maltophilia, X. campestris*	Garbeva et al., 2014
	*Bu. tropica*	Tenorio-Salgado et al., 2013
	*Diaporthe phaseolarum*	Qadri et al., 2015
	*Hypoxylon anthochroum*	Macías-Rubalcava et al., 2018
	*Lysinibacillus* sp.	Che et al., 2017
	*Mu. crispans*	Mitchell et al., 2010
	*Nodulisporium* sp.	Sánchez-Fernández et al., 2016
	*Pa. polymyxa*	Raza et al., 2015
	*Pseudomonas* sp.	Reverchon et al., 2019
	*Streptomyces albulus*	Wu et al., 2015
	*Streptomyces goshikiensis*	Faheem et al., 2015
*Fusarium redolens*	*Ba. velezensis*	Cheffi et al., 2019
*Fusarium sambucinum*	*Muscodor albus*	Corcuff et al., 2011
*Fusarium solani*	*Arthrobacter* sp., *Pseudomonas* sp., *Staphylococcus* sp., *Streptomyces* sp.	Reverchon et al., 2019
	*Bacillus acidiceler*, *Bacillus aerius, Ba. mycoides, Bacillus stratosphericus, Ba. velezensis, Pseudomonas fredderiksbergensis*	Guevara-Avendaño et al., 2019
	*Ba. amyloliquefaciens, Ba. subtilis, Bacillus* sp.	Li et al., 2015
	*Ba. velezensis*	Cheffi et al., 2019
	*Brevundimonas sp*., *Burkholderia* sp.*, Cellulomonas* sp., *Ch. indologenes, Colli. arenae, Colli. fugivorans, Colli. pratensis*., *Flavobacterium* sp.*, Luteibacter* sp.*, Lysobacter antibioticus, Methylobacterium* sp.*, Ps. fluorescens, Se. marcescens Se. plymuthica, Ste. maltophilia, S. atratus, X. campestris*	Garbeva et al., 2014
	*Diaporthe phaseolarum*	Qadri et al., 2015
	*Lysinibacillus* sp.	Che et al., 2017
	*Mu. crispans*	Mitchell et al., 2010
	*X. campestris* pv. *vesicatoria*	Weise et al., 2012
*Fusarium sulphureum*	*Ba. velezensis*	Cheffi et al., 2019
*Fusarium* sp.	*Ba. acidiceles*, *B. stratosphericus, Ba. velezensis*	Guevara-Avendaño et al., 2019
*Ganoderma* sp.	*Mu. crispans*	Mitchell et al., 2010
*Ganoderma boninense*	*Bipolaris* sp., *Diaporthe miriciae*, *Saccharicola bicolor*, *Trichoderma asperellum*	Sim et al., 2019
*Gibberella moniliformis*	*Ba. megaterium*	Munjal et al., 2016
	*Ps. putida*	Sheoran et al., 2015
*Gloeophyllum trabeum*	*Trichoderma pseudokoningii*, *Trichoderma viride*	Wheatley et al., 1997
*Guignardia mangiferae*	*Mu. yucatenensis*	Macías-Rubalcava et al., 2010
*Helminthosporium sativum*	*Ps. chlororaphis, Ps. fluorescens*, *Se. proteomaculans*, *Se. plymuthica*	Popova et al., 2014
*Helminthosporium solani*	*Mu. albus*	Corcuff et al., 2011
*Heterobasidium annosum*	*Ba. velezensis*	Cheffi et al., 2019
*Macrophomina phaseolina*	*Enterobacter cloacae*	Howell, 1988
*Magnaporthe oryzae*	*Ba. megaterium*	Munjal et al., 2016
	*Pseudomonas* sp.	Spence et al., 2014
*Microdochium bolleyi*	*Ba. subtilis, Bu. cepacia*, *Ps. fluorescens*, *Ps. trivialis*, *Se. odorifera*, *Se. plymuthica*, *Ste. maltophilia*, *Ste. rhizophila*	Vespermann et al., 2007
*Monilinia fructicola*	*Ba. amyloliquefaciens*	Gotor-Vila et al., 2017
	*Ba. subtilis, Ba. velezensis*	Gao et al., 2018
	*Debaryomyces hansenii, W. anomalus*	Grzegorczyk et al., 2017
	*Kloeckera apiculate, Pichia membranaefaciens*	Zhang et al., 2017
	*Phaeosphaeria nodorum*	Pimenta et al., 2012
	*Pseudomonas synxantha*	Aiello et al., 2019
	*A. pullulans*	Di Francesco et al., 2020
*Monilinia fructigena*	*Ps. synxantha*	Aiello et al., 2019
	*D. hansenii*, *W. anomalus*	Grzegorczyk et al., 2017
	*A. pullulans*	Di Francesco et al., 2020
*Monilinia laxa*	*Ba. amyloliquefaciens*	Gotor-Vila et al., 2017
	*A. pullulans*	Di Francesco et al., 2020
*Monilinia polystroma*	*A. pullulans*	Di Francesco et al., 2020
*Mucor hiemalis*	*Colli. pratensis*	Garbeva et al., 2014
*Mycosphaerella fijiensis*	*Mu. crispans*	Mitchell et al., 2010
*Ophiostoma piceae*	*Sa. cerevisiae*, *Serratia* sp.	Bruce et al., 2004
*Ophiostoma piliferum*	*Sa. cerevisiae*, *Serratia* sp.	Bruce et al., 2004
*Pectobacterium atrosepticum*	*Mu. albus*	Corcuff et al., 2011
*Penicillium digitatum*	*A. pullulans*	Di Francesco et al., 2015
*Penicillium expansum*	*A. pullulans*	Di Francesco et al., 2015
	*Ba. subtilis*	Gao et al., 2018
	*Ca. sake*	Arrarte et al., 2017
	*G. cerinus + H. osmophila* (bioproduct)	Delgado et al., 2021; Besoain Canales et al., 2017
	*Ps. fluorescens*	Wallace et al., 2017
*Penicillium italicum*	*A. pullulans*	Di Francesco et al., 2015
	*Ba. pumillus*	Morita et al., 2019
*Penicillium* sp.	*Ba. subtilis, Ps. fluorescens*, *Ps. trivialis*, *Se. odorifera*, *Se. plymuthica*, *Ste. maltophilia*, *Ste. rhizophila*	Vespermann et al., 2007
*Peronophythora litchii*	*Ba. amyloliquefaciens, Ba. pumillus*, *Exiguobacterium acetylicum*	Zheng et al., 2019
	*Streptomyces fimicarius*	Xing et al., 2018
*Phoma arachnidicola*	*Ba. pumillus*, *Ba. subtilis*, *Pa. polymyxa*	Liu et al., 2008
*Phoma betae*	*Ba. subtilis, Bu. cepacia*, *Ps. fluorescens*, *Ps. trivialis, Se. odorifera*, *Se. plymuthica*, *Sta. epidermidis*, *Ste. maltophilia*, *Ste. rhizophila*	Vespermann et al., 2007
*Phoma eupyrena*	*Ba. subtilis, Bu. cepacia*, *Ps. fluorescens*, *Ps. trivialis*, *Se. odorifera*, *Se. plymuthica*, *Ste. maltophilia*, *Ste. rhizophila*	Vespermann et al., 2007
*Phoma herbarum*	*T. gamsii*	Chen et al., 2016
*Phomopsis* sp.	*Mu. yucatenensis*	Macías-Rubalcava et al., 2010
*Phyllosticta citricarpa*	*Sa. cerevisiae*	Fialho et al., 2010
*Phytophthora cactorum*	*Ba. velezensis*	Cheffi et al., 2019
*Phytophthora capsici*	*Ba. amyloliquefaciens*, *Ba. velezensis*, *Acinetobacter* sp.	Syed-Ab-Rahman et al., 2019
	*Ba. megaterium*	Munjal et al., 2016
	*Mu. yucatenensis*	Macías-Rubalcava et al., 2010
	*Nodulisporium* sp.	Sánchez-Fernández et al., 2016
	*Ps. putida*	Agisha et al., 2019; Sheoran et al., 2015
*Phytophthora cinnamomi*	*Mu. crispans*	Mitchell et al., 2010
*Phytophthora citrotophthora*	*Ba. velezensis*	Cheffi et al., 2019
*Phytophthora crispans*	*A. pullulans*	Di Francesco et al., 2017
*Phytophthora cryptogea*	*Ba. velezensis*	Cheffi et al., 2019
*Phytophthora megasperma*	*Ba. velezensis*	Cheffi et al., 2019
*Phytophthora nicotianae*	*Ba. pumillus*, *Burkholderia territorii*, *Pseudomonas geniculata*, *Rhodococcus jialingiae*	Riera et al., 2017
*Phytophthora palmivora*	*Mu. crispans*	Mitchell et al., 2010
	*Nodulisporium* sp.	Sánchez-Fernández et al., 2016
	*Phoma* sp.	Strobel et al., 2011
*Phytophthora parasitica*	*Mu. yucatenensis*	Macías-Rubalcava et al., 2010
	*Nodulisporium* sp.	Sánchez-Fernández et al., 2016
*Phytophthora plurivora*	*Ba. velezensis*	Cheffi et al., 2019
*Phytophthora ramorum*	*Ba. velezensis*	Cheffi et al., 2019
*Phytophthora rosacearum*	*Ba. velezensis*	Cheffi et al., 2019
*Phytophthora vexans*	*Ba. velezensis*	Cheffi et al., 2019
*Postia placenta*	*T. pseudokoningii*, *T. viride*	Wheatley et al., 1997
*Pseudomonas syringae*	*Ampelomyces* sp., *Cladosporium* sp.	Naznin et al., 2014
	*Dietzia* sp., *Streptomyces* sp.	Choudoir et al., 2019
*Pythium aphanidermatum*	*Ba. mycoides*	Huang et al., 2018
	*Nodulisporium* sp.	Sánchez-Fernández et al., 2016
*Pythium myriotylum*	*Ba. megaterium*	Munjal et al., 2016
	*Ps. putida*	Sheoran et al., 2015
*Pythium sylvaticum*	*Ba. velezensis*	Cheffi et al., 2019
*Pythium ultimum*	*Ba. subtilis*	Fiddaman and Rossall, 1993
	*Ba. velezensis*	Cheffi et al., 2019
	*Colli. pratensis*	Garbeva et al., 2014
	*D. phaseolarum*	Qadri et al., 2015
	*E. cloacae*	Howell, 1988
	*Lysobacter capsici*	Vlassi et al., 2020
	*Mu. crispans*	Mitchell et al., 2010
	*Nodulisporium* sp	Sánchez-Fernández et al., 2016
	*Ps. donghuensis*	Ossowicki et al., 2017
*Radopholus similis*	*Ba. megaterium*	Munjal et al., 2016
	*Ps. putida*	Sheoran et al., 2015
*Ralstonia solanacearum*	*Ba. amyloliquefaciens*	Raza et al., 2016a
	*Ba. amyloliquefaciens*	Raza et al., 2016b
	*Ba. amyloliquefaciens, Ba. atrophaeus*	Tahir et al., 2017a
	*Ba. megaterium*	Munjal et al., 2016
	*Ps. fluorescens*	Raza et al., 2016a
*Resinicium bicolor*	*Ba. velezensis*	Cheffi et al., 2019
*Rhizoctonia solani*	*Ba. megaterium*	Munjal et al., 2016
	*Ba. mycoides*	Huang et al., 2018
	*Ba. subtilis*	Fiddaman and Rossall, 1993
	*Ba. subtilis, Bu. cepacia, Ps. fluorescens, Ps. trivialis, Se. odorifera, Se. plymuthica, Sta. epidermidis, Ste. maltophilia, Ste. rhizophila*	Kai et al., 2007
	*Ba. subtilis, Ps. trivialis, Se. odorifera, Se. plymuthica, Ste. maltophilia, Ste. rhizophila*	Vespermann et al., 2007
	*Ba. velezensis*	Cheffi et al., 2019
	*Ba. velezensis*	Gao et al., 2017
	*Bu. gladioli*pv. *agricola*	Elshafie et al., 2012
	*D. phaseolarum*	Qadri et al., 2015
	*E. cloacae*	Howell, 1988
	*L. capsici*	Vlassi et al., 2020
	*Mu. crispans*	Mitchell et al., 2010
	*Ps. chlororaphis, Ps. fluorescens, Se. plymuthica, Se. proteomaculans*	Popova et al., 2014
	*Ps. donghuensis*	Ossowicki et al., 2017
	*Ps. putida*	Sheoran et al., 2015
	*Str. platensis*	Wan et al., 2008
	*Streptomyces* sp.	Cordovez et al., 2015
	*X. campestris* pv. *vesicatoria*	Weise et al., 2012
*Rhizoctonia* sp.	*Bu. ambifaria*	Groenhagen et al., 2013
	*Mu. yucatenensis*	Macías-Rubalcava et al., 2010
*Rhizopus stolonifer*	*Ca. pyralidae*, *Pi. kluyveri*	Mewa-Ngongang et al., 2019
	*G. cerinus + H. osmophila* (bioproduct)	Delgado et al., 2021; Besoain Canales et al., 2017
*Sclerophoma pythiphila*	*S. cerevisiae, Serratia* sp.	Bruce et al., 2004
*Sclerotinia minor*	*L. capsici*	Vlassi et al., 2020
*Sclerotinia sclerotiorum*	*Ba. amyloliquefaciens*	Asari et al., 2016
	*Ba. pumillus, Ba. subtilis, Pa. polymyxa*	Liu et al., 2008
	*Ba. subtilis, Bu. cepacia*, *Ps. fluorescens, Ps. trivialis, Se. odorifera*, *Se. plymuthica, Sta. epidermidis, Ste. maltophilia, Ste. rhizophila*	Vespermann et al., 2007
	*Ba. velezensis*	Gao et al., 2017
	*Mu. crispans*	Mitchell et al., 2010
	*Pseudomonas aurantiaca, Pseudomonas corrugata*, *Ps. chlororaphis*, *Ps. fluorescens*	Fernando et al., 2005
	*Ps. fluorescens, Se. proteomaculans, Se. plymuthica*	Popova et al., 2014
	Rhizobacteria consortium	Giorgio et al., 2015
	*Str. albulus*	Wu et al., 2015
	*Str. platensis*	Wan et al., 2008
*Sclerotium rolfsii*	*Bu. tropica*	Tenorio-Salgado et al., 2013
*Sclerotium* sp.	*Trichoderma* sp.	Wonglom et al., 2019
*Scytalidium lignicola*	*T. gamsii*	Chen et al., 2016
*Stagonospora* sp.	*Mu. crispans*	Mitchell et al., 2010
*Tapesia yallundae*	*Mu. crispans*	Mitchell et al., 2010
*Thielaviopsis basicola*	*E. cloacae*	Howell, 1988
*Thielaviopsis ethacetica*	*Pseudomonas* sp.	Freitas et al., 2022
*Ustilaginoidea virens*	*Ba. velezensis*	Myo et al., 2019
*Valsa mali*	*Ba. velezensis*	Gao et al., 2017
*Verticillium dahliae*	*Ba. pumillus, Ba. subtilis, Pa. polymyxa*	Liu et al., 2008
	*Ba. subtilis, Ps. fluorescens, Ps. trivialis, Se. odorifera, Se. plymuthica, Ste. maltophilia, Ste. rhizophila*	Vespermann et al., 2007
	*E. cloacae*	Howell, 1988
	*Mu. crispans*	Mitchell et al., 2010
	*Phoma* sp.	Strobel et al., 2011
	*Ps. donghuensis*	Ossowicki et al., 2017
*Xanthomonas axonopodis*	*Ba. megaterium*	Munjal et al., 2016
	*Mu. crispans*	Mitchell et al., 2010
*Xanthomonas oryzae pv. oryzae*	*Bacillus* sp.	Xie et al., 2018

### Crown gall pathogens

*Agrobacterium tumefaciens* is a soil-borne bacterium that easily infects wound sites in plant hosts and promotes neoplastic growth, causing crown gall tumors and, on a large scale, losses in crop production (Smith and Townsend, 1907). It is also one of the main model organisms in molecular biology, especially for its ability to mediate genetic transformation, which is very useful in fungal and plant biotechnology (Hwang et al., 2017). Another species of this genus is *A. vitis*, the causative agent of crown gall in grapevines (*Vitis vinifera*). It was demonstrated that diseases caused by both *Agrobacterium* species have the potential to be controlled by strains of the rhizobacteria *Pseudomonas fluorescens* and *Serratia plymuthica* (Dandurishvili et al., 2011). *In vitro* analysis indicated that the inhibitory effects against *Agrobacterium* were due to the VOCs emitted by the antagonist strains and not by antibiotics produced by them (Dandurishvili et al., 2011). Furthermore, analysis of the volatile profile of *P. fluorescens* and *S. plymuthica* revealed dimethyl disulfide (DMDS) as a compound emitted by all evaluated strains, and *in vitro* tests showed that this compound could efficiently suppress the growth of the *Agrobacterium* strains (Dandurishvili et al., 2011). The authors also demonstrated that the antagonists suppressed crown gall formation, resulting in a three- to eight-fold decrease in tumor mass, indicating that this molecule might be involved in the suppression of oncogenicity in tomato seedlings (Dandurishvili et al., 2011). Similar results were obtained with such plants infected with *A. vitis*. Accordingly, other studies reported the potential inhibitory activity of VOCs produced by different strains of *Pseudomonas*, as well as *Serratia*, against *Agrobacterium* sp. (Chernin et al., 2013; Popova et al., 2014; Plyuta et al., 2016). The observed effects on the pathogens include reduced biofilm formation, a decrease in the number of living cells composing the biofilm by almost 4-fold and a decreased mycelial growth. Those studies also confirmed DMDS as bioactive compound since it can cause the pathogens inhibition in almost 100%. The ketones 2-nonanone (Popova et al., 2014; Plyuta et al., 2016), 2-heptanone and 2-undecanone (Plyuta et al., 2016) were validated as well. Curiously, Ossowicki et al. (2017) did not observe inhibition of *Agrobacterium* sp. when it was in contact with VOCs produced by *Pseudomonas donghuensis* even that DMDS was identified in its volatilome. Sometimes, different results are reported in literature, regarding the inhibition of pathogens belonging to the same species. There are certain aspects that might influence it, and they will be further discussed.

### Soft rot and bacterial speck pathogens

Of great agronomic importance, the genus *Pectobacterium* has a broad host range since it is isolated from various plant species, including important crops and ornamental plants (Ma et al., 2007). Complete inhibition of *Pe. atrosepticum* was achieved with VOCs emitted by the fungus *Muscodor albus* (Corcuff et al., 2011). Another important phytobacterium is *Pseudomonas syringae*, which has more than 60 pathovars, each of which is capable of infecting specific plant hosts and causing high economic impacts in several crops and trees (Xin et al., 2018), making it one of the most impactful phytopathogens worldwide (Mansfield et al., 2012). *Ps. syrinage* pv. *tomato* is the causal agent of bacterial speck of tomatoes, and it is a model species in molecular studies for understanding bacterial virulence mechanisms (Preston, 2000; Xin et al., 2018). A study showed that the strain *Ps. syringae* pv. *tomato* DC3000 could be inhibited by volatiles from two actinobacteria, *Streptomyces* sp. and *Dietzia* sp. (Choudoir et al., 2019). Although the volatilomes of these antagonists were determined, no functional validation was performed. Moreover, *in vivo* experiments showed that a blend of VOCs (2-methyl-propanol, 3-methyl-butanol, 4-heptanone, 3-octanone, *m*-methyl-anisole, *m*-cresol, 2-phenylethanol and cubenene) from *Ampelomyces* sp. and methyl benzoate from *Cladosporium* sp., reduced disease severity on *Arabidopsis thaliana* leaves infected with *Ps. syringae* pv. *tomato* DC3000 (Naznin et al., 2014). When individually tested, three VOCs from *Ampelomyces* sp., 3-octanone, *m*-cresol and 2-phenylethanol, and synthetic methacrylic acid and isobutyl acetate, both previously identified in *Phoma* sp. volatilome (Naznin et al., 2013), reduced disease severity in *A. thaliana* as well. Yet, the VOC hexadecane, produced by *Paenibacillus polymyxa*, conferred induced systemic resistance to *A. thaliana* against *Ps. syringae* (Park et al., 2013), which represents another mechanism by which VOCs can increase productivity without compromising the environment.

### Bacterial wilt pathogens

The ‘*Ralstonia solanacearum* species complex’ (RSSC) comprises several strains belonging to a wide range of geographical origins, hosts, and pathogenic behaviors (Mansfield et al., 2012), and a recent classification proposed a division into three different species, *R. solanacearum*, *R. pseudosolanacearum* and *R. syzygyi* (Safni et al., 2014; Prior et al., 2016). They are capable of infecting more than 200 plant hosts, causing bacterial wilt and, consequently, leading to extensive economic losses in crops (e.g., tomato, eggplant, potato, tobacco, pepper, banana, peanut, and ginger) and ornamental plants, especially in some developing countries where the disease is endemic (Mansfield et al., 2012; Peeters et al., 2013; Tjou-Tam-Sin et al., 2017). It was shown that *R. solanacearum* was inhibited by up to 45% by *B. amyloliquefaciens* SQR-9 (Raza et al., 2016b), 44% by *B. amyloliquefaciens* T-5 (Raza et al., 2016c), and 51% by *P. fluorescens* (Raza et al., 2016a). Among the 22 and 25 VOCs identified in the volatilome of *B. amyloliquefaciens* SQR-9 and T-5, respectively, nine and 25 compounds were tested *in vitro* against *R. solanacearum* (Raza et al., 2016b, 2016c). Similarly, in both studies, the compounds had weak inhibitory effects when tested individually, reaching up to nearly 10% inhibition. However, different blends of VOCs achieved inhibition rates of 62–85% (Supplementary Table S1; Raza et al., 2016b, 2016c). Among the 13 VOCs identified in *P. fluorescens*, synthetic *m*-xylene and benzaldehyde caused complete inhibition of *R. solanacearum*, while partial inhibition was achieved with toluene, ethylbenzene, DMDS (50–55%), 2-decanol (85%), 2-tridecanol and 1-undecanol (<30%) (Raza et al., 2016a). Thus, VOCs from *Bacillus* and *Pseudomonas* species have been a biocontrol alternative against *R. solanacearum*.

Furthermore, *Bacillus* species seem to have great biocontrol potential (Munjal et al., 2016; Tahir et al., 2017a). The bacteria *B. amyloliquefaciens* and *B. artrophaeus* reduced the growth of *R. solanacearum* by 49 and 47%, respectively (Tahir et al., 2017a); their volatilomes comprised 13 and 10 VOCs, respectively: six were commonly identified in both species, while seven and four were distinct from each bacterium. Fifteen of these VOCs were tested against *R. solanacearum*, and the compounds benzaldehyde (60% inhibition), 1,2-benzisothiazol-3(2H)-one (51%) and 1,3-butadiene (30%) had the most effective results. The volatilome of the two bacteria and the synthetic compounds were also able to reduce wilt index on tobacco seedlings infected with the pathogen. More effectively, it was shown that *B. megaterium* completely inhibited *R. solanacearum* (Munjal et al., 2016). Among the several compounds detected in the *B. megaterium* volatilome, four pyrazine derivative compounds were tested *in vitro* against the pathogen. The synthetic 2-ethyl-3-methyl pyrazine completely inhibited *R. solanacearum*, while 2,5-dimethyl pyrazine, 2-ethyl pyrazine, and 2-methyl pyrazine caused 80, 55, and 32% inhibition, respectively (Munjal et al., 2016). Interestingly, these four pyrazine derivative compounds, also identified in the *Pseudomonas putida* volatilome, had inhibitory effects against *R. pseudosolanacearum*, another species of RSSC (Agisha et al., 2019). The same study showed that dimethyl trisulfide (DMTS) completely inhibited this pathogen.

### Bacterial spot pathogens

The high-ranking positions of important bacterial phytopathogens are occupied by some *Xanthomonas* species (*X. oryzae* pv. *oryzae*, *X. campestris* and *X. axonopodis* pathovars), causing diseases on crops such as rice, cassava, cotton and cultivated brassicas (Mansfield et al., 2012). For such reason, their VOC-mediated biocontrol has been pursued. The growth of *X. oryzae* pv. *oryzae* was reduced by up to 38% by VOCs from *Bacillus* sp. strains (Xie et al., 2018). Among the 12 VOCs identified in the volatilome of the most effective strain, *Bacillus* sp. D13, two compounds, 3,5,5-trimethylhexanol (TMH) and decyl alcohol, were validated with 61 and 54% of inhibition, respectively. Inhibitory effects of TMH were also observed against *X. oryzae* pv. *oryzicola*. Regarding *X. axonopodis* pathovars, studies have demonstrated that they can be also inhibited *via* microbial VOCs (Mitchell et al., 2010; Munjal et al., 2016). Complete inhibition of *X. axonopodis* pv. *citri*, as well as 30 other fungi and bacteria, was achieved using *Muscodor crispans* as antagonist (Mitchell et al., 2010). Volatilome analysis of this fungus identified 17 compounds, but no validation test was performed. Likewise, *X. axonopodis* pv. *punicae* was completely inhibited by *B. megaterium* (Munjal et al., 2016). Although the volatilome of *B. megaterium* was determined (hydrocarbons, acids, alcohols, esters, pyrazines and sulfoxides), validation tests were not performed with this pathogen, only with *Phytophthora capsici, R. soloanacearum* and *Magnaporthe oryzae* (Supplementary Table S1).

### Gray mold pathogens

In relation to phytopathogenic fungi, one of the most critical is the species *Botrytis cinerea*, the causal agent of gray mold, which causes serious problems in agriculture, such as the rotting of postharvest vegetables, fruits, and flowers, especially because it can easily develop resistance to fungicides (Williamson et al., 2007; Dean et al., 2012). *In vitro* and *in vivo* assays demonstrated the potential of VOCs produced by *Ba. velezensis* strains to inhibit *Bo. cinerea* and other pathogens (Table 1; Calvo et al., 2020). In *in vitro* tests, the antagonists were able to inhibit the growth of *Bo. cinerea* by up to 100%. Considering the VOCs identified on the volatilome of the *Ba. velezensis* strains, 11 synthetic compounds presented inhibitory effects against the pathogen, with diacetyl being the most effective bioactive compound at the lowest evaluated dose (Supplementary Table S1 for the complete list of compounds). Individually, the synthetic compounds diacetyl, benzaldehyde and isoamyl alcohol completely inhibited the fungal growth on the fruits (Calvo et al., 2020). The authors also showed that the whole volatilome of the antagonist was able to reduce disease incidence and severity caused by this pathogen on infected grapes by approximately 50%.

The biotechnological potential of *Bacillus* is reinforced against *Bo. cinerea* in other studies. For instance, *Ba. amyloliquefaciens* VOCs suppressed the mycelial growth of the pathogen by 87% (Gotor-Vila et al., 2017). In addition, three of the most abundant VOCs produced by this bacterium, thiophene, 1,3-pentadiene and acetoin (3-hydroxy-2-butanone), were validated as bioactive molecules, showing 83, 63 and 47% inhibition of mycelial growth, respectively (Gotor-Vila et al., 2017). Several other studies also reported inhibition of *Bo. cinerea via* VOCs emitted by *Bacillus* species: *B. amyloliquefaciens* (Asari et al., 2016; Chen et al., 2019), *B. atrophaeus* (Zhang et al., 2013b)*, B. licheniformis* (Chen et al., 2019), *B. pumillus* (Liu et al., 2008), *B. subtilis* (Liu et al., 2008; Gao et al., 2018; Chen et al., 2019) and *B. velezensis* (Jiang et al., 2018; Cheffi et al., 2019; Chen et al., 2019; Myo et al., 2019).

Microorganisms belonging to other taxa also presented inhibitory activity against this pathogen. A recent study showed that the bacterium *Gluconobacter cerinus* and the yeast *Hanseniaspora osmophila* emitted VOCs that were able to inhibit the growth of *Bo. cinerea* by 32 and 39%, respectively (Delgado et al., 2021). Interestingly, a bioproduct composed of those two microorganisms was also tested (Besoain Canales et al., 2017), and it was able to inhibit the pathogen by 86% *via* VOCs, showing a synergic effect of both antagonists (Delgado et al., 2021). This bioproduct was also tested on grape cultivars infected with this pathogen, showing a higher inhibition rate (up to 98%). In addition, both biocontrol agents and the bioproduct were able to inhibit other three phytopathogens *in vitro* and *in vivo* (Table 1). Mostly, the bioproduct had similar or even better results than the antagonists individually tested. Therefore, it demonstrates the biotechnological potential of developing biofungicides based on combined VOC-emitter microorganisms. Furthermore, Parafati et al. (2017) showed that four yeasts, *Wickerhamomyces anomalus*, *Metschnikowia pulcherrima*, *Aureobasidium pullulans* and *Saccharomyces cerevisiae*, strongly reduced conidia germination and mycelial growth of *B. cinerea*. Those antagonists were also able to reduce at least two out of three disease parameters (disease incidence, disease severity and lesion diameter) in strawberries and grapes infected with the pathogen, especially *W. anomalus*, which reduced all parameters by 100%. These yeasts also controlled blue mold decay caused by *Penicillium* species on the same fruit. It is interesting to mention that, in addition to showing the potential use of microbial VOCs to control postharvest diseases, this study demonstrates that the immobilization of microbial cells in a polymeric matrix can be a more suitable and efficient method for biological control in postharvest management.

In this regard, the literature presents more microbial antagonists with such potential *in vitro* and, sometimes, *in vivo*. Regarding bacteria, *Hanseniaspora uvarum* (Qin et al., 2017), *Paenibacillus polymyxa* (Liu et al., 2008), *Pseudomonas stutzeri* (Rojas-Solís et al., 2018), *Stenotrophomonas maltophilia* (Rojas-Solís et al., 2018), *Streptomyces mycarofaciens* (Boukaew et al., 2017)*, Streptomyces platensis* (Wan et al., 2008) and *Streptomyces philanti* (Boukaew et al., 2017) were reported as potential antagonists. As for fungi, especially yeasts, the studies show inhibitory activity induced by VOCs from *Aureobasidium* sp. (Di Francesco et al., 2015; Parafati et al., 2015; Chen et al., 2018; Yalage Don et al., 2020), *Candida* sp. (Arrarte et al., 2017; Chen et al., 2018; Mewa-Ngongang et al., 2019), *Pichia* sp. (Chen et al., 2018; Mewa-Ngongang et al., 2019), *Saccharomyces* sp. (Parafati et al., 2015; Chen et al., 2018), *Monilliela* sp. (Chen et al., 2018), *M. pulcherrima*, *Wickerhamomyces cerevisiae* (Parafati et al., 2015) and *Muscodor crispans* (Mitchell et al., 2010; Table 1). Compounds identified in the volatilome of some of the above mentioned microorganisms, such as 1-octen-3-ol (Zhao et al., 2011), 2-phenylethanol (Di Francesco et al., 2015; Guo et al., 2019a) and DMDS (Rojas-Solís et al., 2018), were validated *in vitro* against the pathogen as well (Supplementary Table S1 for the complete list).

### Anthracnose pathogens

*Colletotrichum* species encompassing critical fungi that causes anthracnose in important fruits, such as mango, avocado, banana and citrus, and in common bean as well (Perfect et al., 1999; Peres et al., 2005; Cannon et al., 2012). In this scenario, the yeast *Debaryomyces nepalensis* has been studied as a sustainable alternative to inhibit *C. gloeosporioides*, being capable of reducing the mycelial growth by 40%, possibly due to the compound 2-phenylethanol (Zhou et al., 2018). The pathogen was also inhibited by VOCs from *Pseudomonas putida* (Sheoran et al., 2015) and, later, some compounds produced by the antagonist were validated, such as DMTS (complete inhibition) and the pyrazine derivative compounds 2,5-dimethyl pyrazine, 2-methyl pyrazine, 2-ethyl-5-methyl pyrazine and 2-ethyl-3,6-dimethyl pyrazine (partial inhibition; Agisha et al., 2019). Furthermore, inhibition of *C. gloeosporioides* was achieved *via* VOCs from *Aureobasidium pullulans, Galactomyces candidum* (Chen et al., 2018), *Bacillus megaterium* (Munjal et al., 2016)*, B. mycoides* (Guevara-Avendaño et al., 2019), *Ba. velezensis* (Cheffi et al., 2019; Guevara-Avendaño et al., 2019)*, Burkholderia tropica* (Tenorio-Salgado et al., 2013), *Ba. subtilis*, as well as by four compounds (benzothiazole, anisole, 3-methylbutanal, and 2,4-di-*tert*-butylthiophenol) identified in the *Ba. subtilis* volatilome (Gao et al., 2018) and *Streptomyces* sp., as well as methyl anthranilate (Gómez et al., 2021; Tables 1; Supplementary Table S1). Interestingly, *Bu. tropica* controlled anthracnose caused by this pathogen on maize plants by almost 80% (Martins et al., 2019).

Moreover, VOCs emitted by *Lysinibacillus* sp. were capable of totally inhibiting the mycelial growth of *C. acutatum* (Che et al., 2017). Validation tests of three VOCs produced by the antagonist showed that 2-ethyl-1-hexanol and benzaldehyde completely inhibited fungal growth, while 2-nonanone presented 61% of inhibition. Furthermore, *A. pullulans* (Di Francesco et al., 2015), *Candida pyralidae* and *Pichia kluyveri* (Mewa-Ngongang et al., 2019) were also reported as antagonists of *C. acutatum*. As for *C. lindemuthianum*, it was reported that this pathogen could be inhibited *in vitro* by *Ba. velezensis* (Gao et al., 2017) and both *in vitro* and *in vivo* (infected beans) by *B. amyloliquefaciens* (Martins et al., 2019). Another species of the genus *Colletotrichum* (*C. fragariae*) was efficiently inhibited by VOCs. Analysis of the volatilome of *Irpex lacteus* detected two VOCs (Koitabashi et al., 2002), lately identified as 5-pentyl-2-furaldehyde and 5-(4-pentenyl)-2-furaldehyde, which completely inhibited *C. fragariae, in vitro* (Koitabashi et al., 2004).

### Fusarium wilt complex

Species of the genus *Fusarium* are soil-borne fungi with a worldwide distribution, affecting almost all existing crops (Bullerman, 2003). Thus, biocontrol alternatives against *Fusarium* species have been also widely pursued. For instance, a study reported that several soil bacterial strains could strongly or partially inhibit the growth of *F. oxysporum*, *F. solani* and *F. culmorum* (Garbeva et al., 2014). The antagonists included bacteria of 16 different genera (e.g., *Burkholderia*, *Collimonas*, *Pseudomonas*, *Serratia* and *Stenotrophomonas*), and they presented inhibitory effects against at least one of the *Fusarium* species (Table 1). Moreover, four *Hypoxylum anthochroum* strains were able to inhibit the growth of *F. oxysporum* in a range of 60 to 80% (Macías-Rubalcava et al., 2018). In total, 36 volatiles were identified in their volatilome, mainly sesquiterpenes (16) and monoterpenes (11). Eucalyptol and 3-methyl-1-butanol were the most abundant in all treatments and, interestingly, they were previously validated as potential biopesticides against *F. oxysporum* (Medina-Romero et al., 2017).

Yet, six *Bacillus* isolates and one *Pseudomonas* sp. partially inhibited *F. solani* (Guevara-Avendaño et al., 2019). Among the VOCs of different chemical classes identified in the volatilome of these strains, six compounds were evaluated *in vitro* (2,3,5-trimethylpyrazine, 2-nonanone, 2-decanone, 2-dodecanone, DMDS and DMTS), and all of them completely inhibited *F. solani* growth, with the exception of 2-dodecanone (38%). Other studies have reported VOC-mediated inhibition *in vitro* of the above-mentioned *Fusarium* species, as well as *F. culmorum*, *F. moniliforme*, *F. flocciferum* and *F. graminearum*. They include efficient antagonists such as *Bacillus* sp. (Liu et al., 2008; Li et al., 2015; Gao et al., 2017; Morita et al., 2019)*, Diaporthe phaseolorum* (Qadri et al., 2015)*, Lysinibacillus* sp. (Che et al., 2017), *M. crispans* (Mitchell et al., 2010)*, Nodulisporium* sp. (Sánchez-Fernández et al., 2016), *P. donghuensis* (Ossowicki et al., 2017), and *Trichoderma* sp. (Chen et al., 2016; Rajani et al., 2021; complete list in Table 1). The synthetic compounds validated in some of these studies are presented in Supplementary Table S1.

Interestingly, *in vivo* inhibition of *Fusarium* species was also achieved. In cherry tomatoes, four *H. anthochroum* strains inhibited the growth of *F. oxysporum* by over 50%, varying the inhibition percentage according to the strain and the incubation period (Macías-Rubalcava et al., 2018). Furthermore, six synthetic VOCs (phenylethyl alcohol, 2-methyl-1-butanol, 3-methyl-1-butanol, eucalyptol, ocimene and terpinolene) identified on the *H. anthochroum* volatilome inhibited the growth of *F. oxysporum* on cherry tomatoes in a dose-dependent manner (Medina-Romero et al., 2017). Individually, the highest doses of 2-methyl-1-butanol, 3-methyl-1-butanol and ocimene completely inhibited the pathogens. Mixtures of the six compounds or of the four alcohols also presented complete inhibition. On tomato leaves infected with *F. oxysporum*, it was observed that the disease symptoms were reduced by spraying *B. velezensis* over them, even as early as the first sampling time (Myo et al., 2019). The plants treated with the antagonist not only suppressed pathogen growth but also increased shoot length and vigor index. Under greenhouse conditions, *B. velezensis* completely controlled *F. oxysporum* growth after up to 14 days. Other studies showed the biocontrol *F. graminearum* and *F. oxysporum* on wheat leaves (Koitabashi et al., 2002), *F. culmorum* and *F. oxysporum* on maize (Tenorio-Salgado et al., 2013) and *F. oxysporum* on watermelon seedlings (Faheem et al., 2015).

### Rice blast pathogens

Other important phytopathogens belong to the family Magnaporthaceae, which comprises approximately 100 fungal species, including plant pathogens that cause diseases to grasses and related species, such as rice, millet, maize and wheat (Ebbole, 2007; Illana et al., 2013). For example, *Magnaporthe oryzae* is present in all rice-growing areas, causing blast disease, the most devastating rice disease worldwide (Illana et al., 2013); for this reason, and because it has been a model in plant-pathogen interaction studies, this pathogen leads a top 10 ranking of fungal pathogens in molecular plant pathology (Dean et al., 2012). In this scenario, a potential bacterial antagonist is *Bacillus megaterium*, which caused complete inhibition of *M. oryzae via* VOCs (Munjal et al., 2016). Among the 20 VOCs identified, four were tested and validated as bioactive compounds: 2-ethyl-3-methyl pyrazine, 2-ethyl pyrazine and 2-methyl pyrazine completely inhibited the pathogen, and 2,5-dimethyl pyrazine inhibited it by up to 74%. It was also reported that VOCs emitted by *Pseudomonas* isolates could inhibit *M. oryzae* by up to almost 50%, and one of these isolates, *Pseudomonas* sp. EA105, could also inhibit appressoria formation (Spence et al., 2014). Curiously, pyrazines (2,5-dimethyl pyrazine, 2-methyl pyrazine, 2-ethyl-5methyl pyrazine and 2-ethyl-3,6-dimethyl pyrazine) were also identified in *P. putida* (Sheoran et al., 2015), and all of them presented inhibitory activity against *M. oryzae* (Agisha et al., 2019).

### Other pathogens

Although we have discussed the VOC-mediated inhibition of important pathogens that have prominent positions within publications in this research area, there are a range of other microbial pathogens that were inhibited *via* microbial VOCs. For instance, the inhibition of food spoilage fungi characterized by aflatoxin production affects postharvest losses during grain storage (Fakruddin et al., 2015; Kumar and Kalita, 2017), such as *Aspergillus flavus* and *A parasiticus* (Jeong et al., 2018; Yang et al., 2019). The studies also explored *Alternaria*, *Cochliobolus*, *Monilinia*, *Penicillium*, *Phytophthora*, *Rhizoctonia, Thielaviopsis* and *Sclerotinia* species. Those and other phytopathogens, that we could not properly discuss in our review, are succinctly presented in Table 1, as well as their respective antagonists. However, the role of VOCs in the inhibition of several phytopathogens ranked as the most important bacteria and fungi (Dean et al., 2012; Mansfield et al., 2012), such as *Xanthomonas campestris* pathovars, *Erwinia amylovira*, *Xylella fastidiosa*, *Dickeya* sp., *Puccinia* sp., *Blumeria graminis*, *Mycospharella graminicola*, *Ustilago maydis*, and *Melampsora lini*, has not yet been explored, and they certainly deserve attention from the scientific community.

To finalize, as we explored in this section, there are a multitude of microorganisms with the potential to inhibit phytopathogens, and it is notable that some bacteria and yeasts have stood out as potential biocontrol agents, such as *Bacillus* (especially *B. amyloliquefaciens*, *B. subtilis* and *B. velezensis*), *Pseudomonas* (especially *P. chlororaphis*, *P. fluorescens* and *P. putida*), *Serratia* and *Streptomyces* species. The VOCs emitted by them display promising futures as efficient and ecological alternatives for controlling diseases in crops.

## VOCs cause several molecular changes on phytopathogens

**Figure 2:**
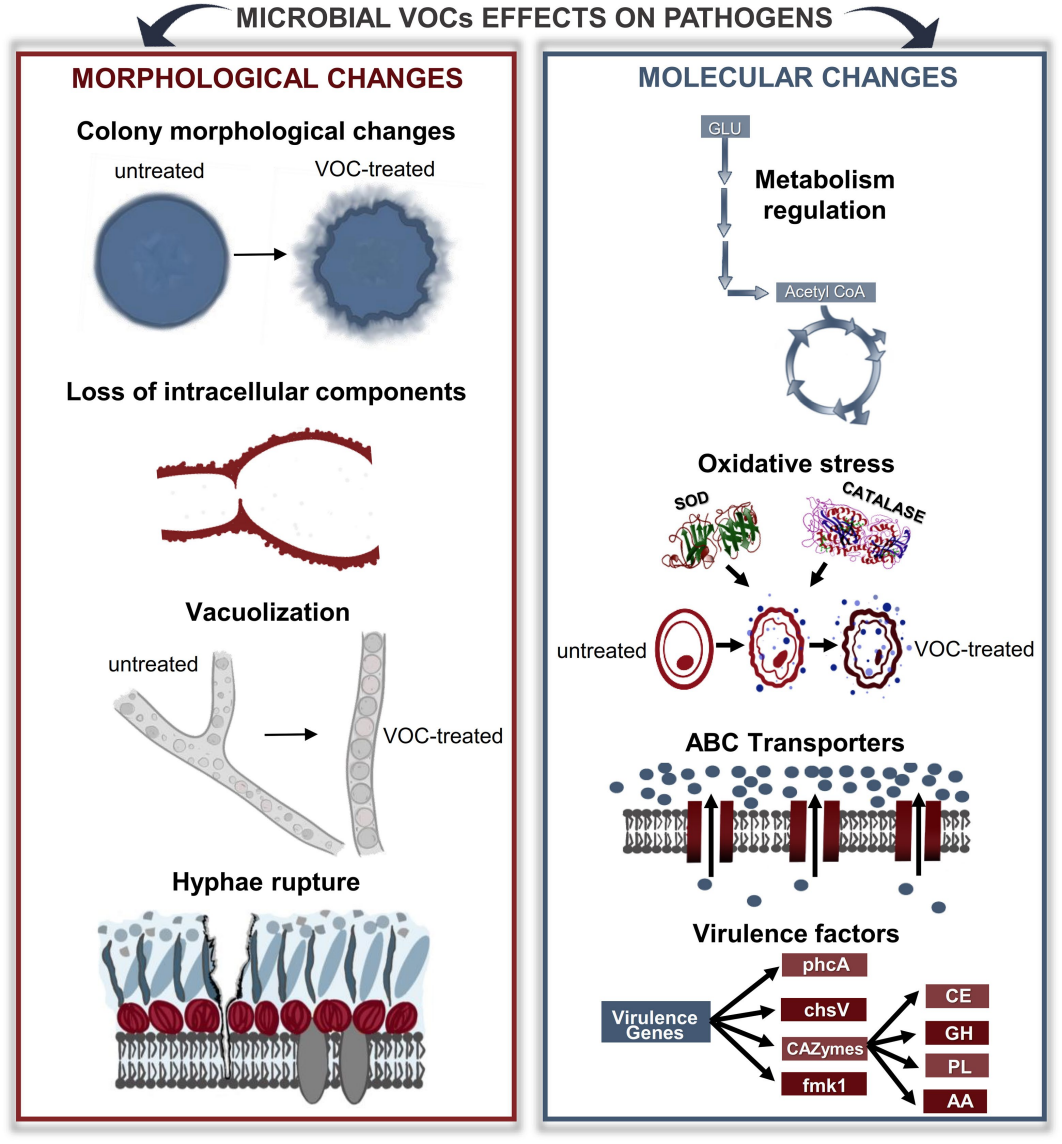
Microbial VOCs affect the morphology and physiology of phytopathogens. Through diverse approaches, it is possible to understand the changes that microbial VOCs cause in phytopathogens. Microscopy analyses revealed changes in the morphology of the colonies, loss of intracellular components, vacuolization, and cell rupture. In addition, omics-approaching studies have shown that many genes and proteins are up-and downregulated under the effects of VOCs, thus interfering with several important metabolic pathways, such as carbon metabolism and oxidative stress. GLU, glucose; SOD, superoxide dismutase; ABC, ATP-binding cassette; CAZymes, carbohydrate-activated enzymes; CE, carbohydrate esterases; GH, glycosyl hydrolases; PL, polysaccharide lyases; AA, auxiliary activities.

Although it is well known that microbial VOCs can inhibit the growth of phytopathogens, the mechanisms involved in such processes remain poorly understood. Recent studies suggest that VOCs affect phytopathogens by modulating the activity of specific enzymes plus changing motility and protein production, which subsequently influence growth, cell morphology and virulence factors (Figure 2). In this section, we will present what these studies have reported and discussed about the mechanisms by which VOCs cause inhibition of these microorganisms.

### VOCs induce critical morphological changes

Microscopy has been the most common technique used to assess and observe the effects of VOCs on phytopathogens. Transmission electron microscopy (TEM), scanning electron microscopy (SEM) and optical microscopy are some types of techniques used to observe changes in cell and organelle morphology and consequently, improve the understanding of the mechanisms involved. Pathogenic bacteria, oomycetes and fungi are responsible for major losses in crops (Toth and Birch, 2005; Makovitzki et al., 2007; Dean et al., 2012; Mansfield et al., 2012; Drenth and Guest, 2016), and regarding the biocontrol of such microorganisms, it is important to bear in mind that bacteria and fungi have great differences in cell components, structure and morphology. While fungi can be multicellular eukaryotes, bacteria are single-celled prokaryotes without nuclei and other membrane-bound organelles and with asexual reproduction. Thus, the morphological changes caused by VOCs in bacteria are mostly associated with degradation of the outer membrane, disruption of the cytoplasmic membrane and loss of internal material that directly affects the bacterial ability to infect host plants (Helander et al., 1998; Bouhdid et al., 2009; Goel, 2017).

For instance, in the presence of VOCs from the rhizobacteria *Bacillus subtilis*, the colonies of *Clavibacter michiganensis* subsp. *sepedonicus* became internally distorted, and cells were damaged (Rajer et al., 2017). A wide range of abnormalities in the pathogen cells, such as misshapenness, disintegration of cells, formation of inclusions, movement of cytoplasmic content toward the ruptured cell walls or cytoplasmic membranes, and a lack of cytoplasmic content or fragmented cytoplasm were observed. Other studies showed similar changes in *Ralstonia solanacearum* morphology after exposing this pathogen to VOCs produced by antagonistic strains (Raza et al., 2016a,b,c; Tahir et al., 2017a). Microbial VOCs were capable of suppressing biofilm formation (Plyuta et al., 2016; Raza et al., 2016a,b,c), an important bacterial defense factor against external conditions that also plays a role in virulence (Danhorn and Fuqua, 2007). In addition, it has been reported that microbial VOCs have effects on virulence factors of phytopathogens, decreasing motility traits (swarming, swimming, and chemotaxis), production of antioxidant enzymes (e.g., superoxide dismutase and catalase) and exopolysaccharides, and the capability of colonizing tomato roots (Raza et al., 2016a,b,c).

Regarding phytopathogenic fungi, morphological changes were shown in their hyphal and mycelial structures. For instance, branched hyphae became straight and tiny when the saprotrophic fungus *Mucor hiemalis* was exposed to volatiles produced by *Collimonas* sp., a mycophagous soil bacterium (Garbeva et al., 2014). These changes were also observed on *Fusarium* sp. exposed to VOCs produced by *Bacillus* sp.; the pathogen developed shorter hyphal segments between septa and slightly distorted, ramified and curved hyphae at the edge of its colonies (Guevara-Avendaño et al., 2019). Flattened and perforated hyphae were observed in *F. oxysporum*, while shrinkage and perforation occurred in *Penicillium digitatum*, when both pathogens were treated with isooctanol, an alcohol identified in the *Corrallococcus* sp. (Ye et al., 2020). An increase in vacuolization processes (i.e., enlargement of vacuoles in mycelia, conidiophores or hyphae) has also been observed in some fungi inhibited by microbial VOCs or the respective synthetic compounds (Chaurasia et al., 2005; Tenorio-Salgado et al., 2013; Giorgio et al., 2015; Medina-Romero et al., 2017; Huang et al., 2018; Freitas et al., 2022). According to Macías-Rubalcava et al. (2018), these changes can compromise the respiration of microorganisms and affect cell membrane permeability, as was observed in the endophytic fungus *Hypoxylon anthochroum* (Macías-Rubalcava et al., 2018). In fact, depending on the severity of the vacuolization, hyphae rupture can also occur (Medina-Romero et al., 2017). Furthermore, large amounts of balloon-shaped cells, aggregation of cytoplasm and protoplasm, degradation of cell walls, cell breakage and leakage of intracellular substances on *F. oxysporum* were also observed due to exposure to volatiles emitted by *Bacillus tropica* (Tenorio-Salgado et al., 2013).

Plasmatic membranes of fungi can also be affected by microbial VOCs. The VOCs of *Saccharomyces cerevisiae* increased the lipid peroxidation of the pathogen *Phyllosticta citricarpa* and, consequently, decreased membrane fluidity, increased permeability to H^+^ and other ions and eventual cellular rupture, which directly affected the progress of the disease symptoms on plants. Such results corroborate the increase in membrane permeability of pathogenic spores of *F. culmorum* and *Cochliobolus sativus* as a result of the decrease in the efflux of K^+^ ions into the intracellular space caused by the compounds methyl propanoate and methyl prop-2-enoate (Kaddes et al., 2019). Moreover, volatiles emitted by *B. subtilis* and the pure compound benzothiazole affected the membrane ergosterol content of *Monilinia fructicola* and inhibited the activity of the enzymes pectinase and cellulase, which have an important role in cell wall and cell membrane integrity (Zhou et al., 2019). These recent findings elucidate that VOCs can directly alter membrane fluidity, resulting in leakage of intracellular contents and loss of cell viability, as well as triggering a cascade reaction to avoid oxidative stress.

Other effects on membranes were also observed when fungi were exposed to VOCs, such as strongly retracted plasma membrane in F*. oxysporum, S. sclerotiorum* (Wu et al., 2015) and *Thielaviopsis ethacetica* (Freitas et al., 2022), congregated and browned protoplasms in *S. sclerotiorum* (Liu et al., 2008), abnormal morphology of appressoria, an alteration that could affect the pathogen infection ability, in *Peronophythora litchi* (Xing et al., 2018) and *M. fructicola* (Zhou et al., 2019). Additionally, it was also shown that the intracellular components in *M. fructicola* were destroyed and an empty shell was formed, corroborating the idea that VOCs can destroy the barrier function of the cell wall and cell membrane (Zhou et al., 2019).

Furthermore, studies have also shown that VOCs mediate changes in the production and germination of conidia and spores. Conidia became swollen and thick-walled, and there was suppression of normal formation of sporangia and oogonia in *Pythium afertile* exposed to microbial VOCs (Chaurasia et al., 2005). The same study reported that the transverse and longitudinal septae completely disappeared in hyphae of *Alternaria alternata*, while conidiophores became vegetative and stunted in *Cladosporium oxysporum* (Chaurasia et al., 2005). Yet, other structural effects such as cell wall granulation, reduced number of mitochondria, destruction of organelles and internal cell darkening can be caused by VOCs, as it was observed in *T. ethacetica* (Freitas et al., 2022).

### VOCs alter several metabolic pathways

More recently, many studies have incorporated modern molecular biology approaches to investigate the mechanisms related to the growth inhibition of phytopathogens. For instance, it was shown that treatment with the microbial VOC α-humulene inhibited the expression of two virulence genes, *fmk1* and *chsV*, of the phytopathogen *Fusarium oxysporum* (Minerdi et al., 2009). The *fmk1* gene encodes a mitogen-activated protein kinase that controls a number of key steps that regulate plant infection and fungal growth in living plant tissue (Roncero et al., 2003). The *chsV* gene encodes a protein homologous to class V chitin synthases that plays an important role in cell wall biosynthesis for pathogen adhesion (Liu et al., 2017). Curiously, in addition to the effects on *Fusarium* wilt pathogen growth and cell wall stability, α-humulene was also validated as a lettuce growth promoter biomolecule (Minerdi et al., 2011).

VOCs emitted by *Bacillus amyloliquefaciens* presented effects over the virulence factors of the phytopathogen *Ralstonia solanacearum* as well (Raza et al., 2016c). The VOCs produced were able to significantly reduce the expression of antioxidant activity-related genes *katG* and *sobB* and the transcriptional regulator *PhcA* by more than two times. *PhcA* is a cell density-dependent regulator that controls the expression of virulence factors such as extracellular polysaccharides (EPS), cell wall-degrading enzymes such as endoglucanases, and bacterial motility (Genin et al., 2005; Hikichi et al., 2017; Khokhani et al., 2017). Decreases in the expression of genes related to flagellum-dependent cell and twitching motility were also observed, supported by the motility trait assay results. On *Xanthomonas oryzae* pv. *oryzae*, after exposure to VOCs from a *Bacillus* strain, the transcriptional levels of the *motA*, *motC* (motility) and *rpf* (biofilm and virulence) genes were downregulated (Xie et al., 2018). Thus, VOCs regulate important genes related to survival, motility and pathogenicity.

To perform a deeper evaluation of VOC effects, omics studies have also been carried out. Proteomics analysis has shown that VOCs downregulate pathogenic proteins related not only to virulence (*PhcA*, proteins related to the type III and type IV secretion systems, EPS and chemotaxis-related) but also to the central metabolism of carbohydrates and amino acids, translation, protein folding and antioxidant activity (Raza et al., 2016a,b). These alterations not only negatively affected pathogen growth but also inhibited the virulence traits, root colonization, and metabolic activity of the pathogen, restricting its movements to colonize and infect plants (Tahir et al., 2017a).

In addition, other proteins seem to be important in the inhibition process. Proteins involved in antioxidant activity, such as thiol peroxidase, catalase, superoxide dismutase and polyphenol oxidase, were downregulated by more than two times, and their activity decreased by more than 60%. These enzymes not only neutralize reactive oxygen species (ROS) accumulated during the metabolism of the pathogen (Soto et al., 2006; Pfeilmeier et al., 2016) but also interact with the oxidative burst generated by their host plants during bacterial invasion and spreading (Huang and Allen, 2000; Afzal et al., 2014; Sahu et al., 2017). *PhcA*, which was downregulated in *R. solanacearum* (Raza et al., 2016a,b), also regulates endoglucanase; however, the activity of this enzyme was not affected in the same way. Controversially, another study showed that genes involved in redox reaction, as well as cell wall synthesis, were upregulated in *F. oxysporum* and *Penicillium digitatum* (Ye et al., 2020). Results also showed a burst of ROS in the pathogen cells. The authors suggested that the increase in the redox genes might be related to the attempt of neutralizing ROS.

Furthermore, it was shown that VOCs can change the synthesis of critical proteins involved in transport, genetic information processing, cellular processes, and metabolism (Fialho et al., 2016). Interestingly, several proteins related to carbohydrate and energy metabolism were downregulated, such as enolase, glyceraldehyde-3-phosphate dehydrogenase, pyruvate dehydrogenase and 6-phosphogluconate dehydrogenase. On the other hand, there was a noticeable increase in the expression of enzymes related to secondary metabolism, such as cyclase/dehydrase-like protein, ESC reductase and tetrahydroxynaphthalene reductase. These enzymes might be necessary to protect *Phyllosticta citricarpa* against VOCs, since they have a role in processes that stabilize the cell wall improve pathogenesis and increase stress resistance against radiation, oxidizing agents and antifungal compounds (Paolo et al., 2006; Atanasova et al., 2013).

More recently, it was demonstrated that the VOC S-methyl methanethiosulfonate has high protective potential *in planta* by affecting the oomycete *Phytophthora infestans* (Chinchilla et al., 2019). Proteomics analysis of the pathogen exposed to the pure compound revealed that 80% of the proteins were downregulated or undetectable. Massive downregulation of proteins involved in redox balance, secretion, posttranslational modification, protein turnover, signal transduction and transcription were also observed. In addition to demonstrating the anti-oomycete activity of S-methyl methanethiosulfonate through a multitarget mode of action, the study also revealed that exposure to closely related individual compounds from the same functional class (such as DMDS and DMTS) differently affects the inhibition and proteome of the pathogen.

An analysis comparing the effects of 2-phenyethanol (2-PE) and the volatilome of the yeast *Candida intermedia* on the global synthesis of proteins of the pathogen *Aspergillus carbonarius* revealed that both yeast volatilome and 2-PE treatments immediately inhibited mycelial growth and greatly reduced the production of the mycotoxin ochratoxin A (Tilocca et al., 2019). The proteomic investigation of both treatments revealed that 2-PE partly reproduced the metabolic changes promoted by *C. intermedia*, and the differences consisted of abundance levels of commonly identified proteins (Tilocca et al., 2019). Most global changes in protein expression patterns were identified in protein biosynthesis, proliferative activity, mitochondrial metabolism, and particularly in detoxification of toxic substances (Tilocca et al., 2019). Both treatments increased the levels of EuKaryotic Orthologous Groups (KOG) classes related to plasma membrane H^+^ transporting ATPase, E1-E2 ATPase, haloacid dehalogenase-like hydrolase, cation transporter/ATPase N-terminus and proteins involved in intra-and intercellular trafficking of vesicles. This shows that the single molecule and natural blend of VOCs target specific points of fungal metabolic pathways, resulting in responses that prevent fungal infections (Tilocca et al., 2019). Remarkably, both treatments stimulated stress response mechanisms in the fungal cells, with different origins for each sample. While 2-PE led to greater amounts of sphingosine phosphate lyase KOG class, which is linked to a cascade of catabolic reactions responding to “heat stress” in yeasts (Cowart and Obeid, 2007; Chen et al., 2013), the synthetic yeast mix volatilome treatment registered a higher expression of proteins with carbon-nitrogen hydrolase region KOG class, which has been matched with “resistance to chemicals” by yeasts (Bork and Koonin, 1994; Suda et al., 2003).

Remarkably, microbial VOCs can damage the DNA of phytopathogens. Ye et al. (2020) observed through fluorescence microscopy that *F. oxysporum* and *Pe. digitatum* presented phosphatidylserine externalization and DNA fragmentation after treated with the isooctanol. Recently, Freitas et al. (2022) showed that VOCs from *Pseudomonas* strains also caused DNA damage on the sugarcane pathogenic fungus *Thielaviopsis ethacetica*. Diverse genes involved in DNA damage response were upregulated, in comparison with phytopathogen cultivated in the absence of VOCs, and, interestingly, DNA cleavage and chromatin fragmentation were indicated *via* Fourier-transform infrared microspectroscopy, corroborating with the transcriptomic analysis. In addition, downregulation of several genes involved in metabolic pathways and morphological changes on mycelia contributed to the growth inhibition of this fungus. Therefore, DNA damage seems to be a crucial VOC-mediated mechanism of phytopathogens inhibition. These findings also demonstrate the importance of including omic approaches and other advanced technologies in order to investigate the molecular mechanisms involved in such processes.

Curiously, some mechanisms triggered by VOCs occur in the plant, helping in the disease control process. Volatiles emitted by *B. subtilis* and the pure compound benzothiazole not only inhibited *Monilinia fructicola* growth *in vitro* but also modulated the activity of cellulase, polygalacturonase, peroxidase, polyphenol oxidase, catalase and superoxide dismutase on infected peach fruits exposed to the volatiles (Zhou et al., 2019). These findings indicate that the damage caused by the phytopathogen on the fruit was mitigated by reducing the activity of the degrading enzymes and by increasing the activity of the antioxidant enzymes, which was also corroborated by the lower concentration of molecules that indicate oxidative stress, on treated fruits. As shown above, until now, few studies have focused on the molecular mechanisms and global analysis involved in pathogen inhibition *via* microbial VOCs (Fialho et al., 2016; Raza et al., 2016a,b,c; Chinchilla et al., 2019; Tilocca et al., 2019; Freitas et al., 2022). Thus, it is still too early to have a consensus on a comprehensive and well-established path by which microbial VOCs inhibit phytopathogens.

## The path to a well established VOC-based bioproduct has a few challenges yet

As we perceived in the previous sections, VOCs have great biotechnological potential as a sustainable tool against phytopathogens, reducing the use of agrochemicals and, consequently, mitigating damage to the environment and human and animal health. However, many questions and uncertainties are still attributed to the control of phytopathogens using VOCs, from the search for an antagonist microorganism to the launch of a final product on the market. Some questions that need to be addressed are as follows: is the use of VOCs harmful to us? What about their impact on other living organisms and the environment? What is the minimum dose/concentration to be applied, that is also efficient? How long do they remain in the environment? Does the food absorb them? How long are they effective? Should they be applied pre, during or postharvest? These questions and others will be discussed hereafter.

In the last section, we mentioned that the molecular mechanisms by which VOCs act on phytopathogens remain poorly understood. Nevertheless, some issues about these mechanisms are frequently addressed, and one of them is whether they are biocide or biostatic. The inhibition caused by VOCs is biocidal when it results in the death of the phytopathogen or biostatic when it only ceases microorganism growth as long as the causal conditions are maintained. For instance, the phytopathogen *Ralsotonia solanacearum* was effectively inhibited by *Bacillus amyloliquefaciens* strains (Raza et al., 2016b) and *Pseudomonas fluorescens* (Raza et al., 2016a), but after removing the pathogen from the culture system, its growth rate was resumed, showing that both antagonists have a bacteriostatic effect. On the other hand, the phytopathogen *Fusarium solani*, after being inhibited by VOCs of *B. velezensis*, did not present mycelial growth when it was transferred to new plates of PDA medium (Cheffi et al., 2019). The damage caused to this pathogen was enough to cause a fungicidal effect. The reason that some VOCs only cease the growth of pathogens while others kill them is still unclear, but it shows that the development of an efficient bioproduct requires a careful analysis of such effects.

Another issue concerning the use of microorganisms or specific volatiles as a product is the difference of results observed with the same pathogen. We previously showed that sometimes, the results obtained with the synthetic volatiles on validation tests are not the same as those with the antagonist strain. For instance, the growth inhibition of *Agrobacterium tumefaciens* by VOCs from *Serratia* sp., mainly due to the compound DMDS, was observed by Dandurishvili et al. (2011), Chernin et al. (2013), Popova et al. (2014) and Plyuta et al. (2016), but not by Ossowicki et al. (2017). Thus, we need to expand our knowledge about which bioactive molecules act individually or in combination and what respective concentrations are necessary to cause inhibition. This can be a tough task, since in our review of the literature, we observed very distinct assays to identify microbial antagonists, several approaches to determine their volatilomes and to perform functional validation of each compound. In addition, microorganisms exhibit substantial genetic and metabolic plasticity, which can lead to different responses to those observed in previous tests or tests performed by different research groups.

One of the main questions about the formulation of a product and its application in the field concerns whether the microorganism will be able to produce the bioactive compounds and in a necessary concentration to inhibit the phytopathogens. Usually, *in vitro* tests are able to determine a minimum dose of compounds or microorganisms with a biocidal or a durable biostatic effect against the pathogen. Nonetheless, we have already illustrated that different cultivation conditions can influence the volatilome of a single microorganism, so in the soil, it would be no different. The physical and nutritional attributes of soil, cultivated crops, climate, human intervention, and rhizospheric microbial community change over time, directly affecting the production and composition of the volatilomes (Peñuelas et al., 2014; Kai et al., 2016; De Boer et al., 2019), as well as resulting in a dynamic and specific system. Accordingly, Bastos and Magan (2007) observed clear differences in the VOC profiles produced by the microbial community of three different soils (sandy loam, calcareous clay soil, and volcanic ash), varying water potential, temperature, and nutrient source. Given the diversity of soils worldwide, the ability of microorganisms to produce VOCs capable of inhibiting pathogens might become limited to certain specific conditions. However, a very recent study reported that a bioproduct has a high capacity to inhibit grape pathogens *via* VOCs in *in vitro* and *in vivo* assays (see further sections) (Besoain Canales et al., 2017; Delgado et al., 2021). It may be prepared in culture media ensuring the production of the molecules of interest (Besoain Canales et al., 2017). Besides, such results demonstrate the synergism of the two antagonists and shed light upon a path that combining different microorganisms can be an interesting alternative to formulate a highly efficient biocontrol product.

Another issue concerns the shelf life of a biological product, and some nanotechnology strategies have been suggested. For instance, the antagonist yeasts *Wickerhamomyces anomalous* and *Aureobasidium pullulans* were encapsulated in hydrogel spheres and applied to strawberry fruits (*Fragaria ananassa*) and tangerines (*Citrus reticulata*) (Parafati et al., 2017). Although the inhibitory effects were promising, the yeasts had a survival time of only 10 days. The minimum shelf life of a biocontrol formula should be approximately 8 months so it could be commercialized (Nakkeeran et al., 2006). It is important to mention that encapsulation requires proper evaluation of the process, since it can alter some physiological properties of immobilized cells and even decrease cell viability (Rathore et al., 2013). However, any formulation consists of an important developing stage that demands much knowledge, technique, time and many tests until a long-lasting output is obtained.

An alternative for a product is to produce it based on bioactive molecules without including microorganisms in the composition. First, it is necessary to know the bioactive compounds and their efficiency as either single molecules or blends to create a viable product. As we have already shown, some individual compounds perform a complete inhibition of the pathogen and, in other cases, it is the mixture of compounds that have a maximum inhibitory activity. However, it is important to mention that occasionally, not all compounds are commercially available, and usually, they have a high cost for just a tiny amount. Consequently, the ability to test many different concentrations and combinations of compounds is limited. However, when bioactive compounds are identified, it is possible to think about new delivery strategies. Recently, it was shown that lipid nanoparticles can be promising carriers for encapsulating VOCs due to their physiochemical properties and biological activity (Fincheira et al., 2020). In addition, it has already been shown that polyamide-based microcapsules with TiO_2_ can be successfully used to trigger the release of volatile organic compounds by UV-A irradiation (Marques et al., 2013). Recently, Mun and Townley (2020) reviewed the different types of nanoparticles to encapsulate plant VOCs, but we believe that the same can be true for microbial volatiles, which is a valuable alternative for using these compounds in the field.

Nevertheless, it is important to know if it is free of risks to human, animal, and environmental health. Even in small quantities, VOCs are extremely active molecules, and they rapidly spread into the atmosphere, thus having the possibility to trigger damage to nontarget organisms (Sharifi and Ryu, 2018). Although VOCs produced by *Muscodor albus* have high efficiency in controlling several fungi, one of its metabolites is toxic to human health, preventing the further use of this biocontrol agent (Braun et al., 2012; Romanazzi et al., 2012). Furthermore, the compound DMDS is a widely found and very controversial compound, since it has promoted growth of *A. thaliana* (Groenhagen et al., 2013) and inhibited some phytopathogens (Supplementary Table S1), but also inhibited the growth of *Nicotiana attenuata* plants (Meldau et al., 2013) and had toxic effects on nematodes and *Drosophila melanogaster* (Popova et al., 2014), as well as on mammals, such as rats, mice and humans (Korpi et al., 2009). Considering that VOCs can be noxious to humans, several *in vitro* and *in vivo* studies are necessary to obtain proper information about their safety and then to construct a robust VOCs database with this information (Korpi et al., 2009).

In this sense, it is also important to know whether VOCs are always benefic to plants, or if they can be harmful. In many cases, microbial VOCs promote changes on plant metabolism (e.g., modulation of biosynthesis and signaling pathways of phytohormones or production of plant VOCs that acts as attractor to natural enemies of the pathogens) resulting in a more effective defense response (Schausberger et al., 2012; Pineda et al., 2013; Sharifi and Ryu, 2016, 2018). Nevertheless, microbial VOCs can sometimes be harmful to plants. For instance, in a screening of 20 *Trichoderma* strains, although nine strains promoted *Arabidopsis* growth, VOCs from *T. atroviride* decreased the total fresh weight and the total chlorophyll content of plants and damaged the plant leaves (Lee et al., 2016). Another study that screened *Trichoderma* species for plant growth promoters found that plants co-cultivated with *T. asperellum* had a lower biomass of up to 74% (Nieto-Jacobo et al., 2017). Although *Trichoderma* species have biofungicidal potential, as we have already shown, not all strains or species are harmless to plants. From the same perspective, as we discussed just above, DMDS can promote or inhibit plant growth. Thus, further investigation is needed to better understand the beneficial effects of VOCs on plants, as well as their potential negative effects, since they can cause undesirable effects.

Although our focus is on the action of VOCs on phytopathogens, knowing how plants sense the molecules is important since VOCs also trigger some plant mechanisms and, in turn, can aid in the inhibition process. Therefore, how do plants sense microbial VOCs? Currently, the understanding of the perception of VOCs by plants is based on well-characterized studies of plant volatiles, which are compounds of diverse chemical classes emitted during biotic stresses and injuries, and they are responsible for plant–plant, plant–animal and plant-microbe interactions (Matsui, 2006; Maffei, 2010; Scala et al., 2013). Thus, based on studies about these volatiles and how plants sense them (Choh et al., 2004; Holopainen, 2004; Mirabella et al., 2015), we gain insights into microbial VOCs receptors in plants and their perception, but a proper investigation about them is still necessary to better understand their occurrence, their mode of action, and whether they can distinguish VOCs from beneficial and pathogenic microorganisms.

These and other questions that we did not address need to be answered to produce more sustainable biocontrol products on the market. From then on, we will reduce our dependence on agrochemicals by integrating microbial VOC-based formulas in crop management. The potential of volatile organic compounds is enormous, and with further studies, the bottlenecks that still hold back their wide usage in agriculture can be overcome.

## Conclusion

By 2050, the global population is expected to reach over 10 billion, and this population increase will require doubling the level of food production. To ensure adequate global food for this growing population, we need to maximize crop production and minimize both the demand for financial resources and the impact on the environment. In this regard, the use of biotechnology with microbial products is now considered a valuable addition to sustainable agriculture. Bacteria and fungi can inhibit the growth of plant pathogens through VOCs, including those of greater scientific and economic importance. Here, we showed that *Agrobacterium*, *Blumeria*, *Botrytis*, *Colletotrichum*, *Fusarium*, *Magnaporthe*, *Pectobacterium*, *Ralstonia* and *Xanthomonas* species were successfully controlled in *in vitro* and *in vivo* assays. As antagonists, *Bacillus* (*B. amyloliquefaciens*, *B. subtilis* and *B. velezensis*), *Pseudomonas* (e.g., *P. chlororaphis*, *P. fluorescens* and *P. putida*), *Serratia* and *Streptomyces* species stand out, and those genera represent promise for the formulation of efficient biocontrol products, as well as several bioactive compounds that they are able to produce. However, despite this potential, their biological activities are not properly explored in the field because there are still numerous scientific questions to be addressed. In this sense, different biotechnology tools have allowed us to better understand the mechanisms by which microbial VOCs inhibit phytopathogens. We still have to learn more about these mechanisms, as well as many other aspects of biosafety, to develop a bioproduct. Despite these concerns, it is very clear that beneficial VOC-producing microorganisms have vast biotechnological potential in helping to enhance sustainable agriculture.

## Author contributions

OA and JO conceived the idea of the manuscript. OA, NA, BD, CF, JO, C-MR, and LC: co-wrote the main text. NA, OA, and CF: designed the figures. OA designed the Supplementary Tables. OA, JO, and CR critically revised the manuscript. All authors approved the final draft.

## Funding

This work was supported by the São Paulo Research Foundation (FAPESP; 2017/24395-5, 2019/08522-2, 2018/04184-2 and 2022/00474-1), Conselho Nacional de Desenvolvimento Científico e Tecnológico (CNPq; project 870370/1997-9), Coordenação de Aperfeiçoamento de Pessoal de Nível Superior (Capes; 88887.514087/2020-00 and 8887.144410/2017-00), and by the National Research Foundation of Korea (NRF) grant funded by the Korea government (MSIP) under Grant numbers 2021-212 and 2021-128, KRIBB Initiate Program.

## Conflict of interest

The authors declare that the research was conducted in the absence of any commercial or financial relationships that could be construed as a potential conflict of interest.

## Publisher’s note

All claims expressed in this article are solely those of the authors and do not necessarily represent those of their affiliated organizations, or those of the publisher, the editors and the reviewers. Any product that may be evaluated in this article, or claim that may be made by its manufacturer, is not guaranteed or endorsed by the publisher.

## Supplementary material

The Supplementary material for this article can be found online at: https://www.frontiersin.org/articles/10.3389/fmicb.2022.951130/full#supplementary-material
