# Supplementary material for: The power of the smallest: The inhibitory activity of microbial volatile organic compounds against phytopathogens
Source: Front Microbiol. 2023 Jan 4;13:951130. doi: 10.3389/fmicb.2022.951130 (PMC9845590; doi:10.3389/fmicb.2022.951130)
Supplement: Supplementary file 1 [file Table_1.pdf]

Supplementary table 1. Microbial volatile organic compounds (VOCs) validated *(in vitro* and/or *in vivo*) as bioactive inhibitors of phytopathogens.

Compounds	Chemical classes	PubChem ID	mVOC*	Identified in	phytopathogen inhibited	Inhibitory concentrations	References
1-(2-aminophenyl)-ethanone	benzenoid / ketone / amine / nitrogen compound	11086	Y	Bacteria	*Peronophythora litchii*	EC50 = 170.3 mg/L	Zheng et al., 2019a
1,2-benzisothiazol-3-(2H)-one	sulfur compound / benzenoid / lactame / thiazole / isothiazole / ketone / nitrogen compound	17520	Y	Bacteria	*Ralstonia solanacearum*	5.0 mg = ±60%	Tahir et al., 2017a
1,3,5-trichloro-2-methoxy benzene	benzenoid / ester / halogenated compound	6884	Y	Bacteria	*Rhizoctonia solani*	100mM and 1M = 100%	Cordovez et al., 2015
1,3-butadiene	alkene	7845	N	Bacteria	*Ra. solanacearum*	6.14 mg = 40%	Tahir et al., 2017a
1,3-pentadiene	alkene	62204	Y	Bacteria	*Botrytis cinerea*	0.67 µL/mL = 63%	Gotor-Vila et al., 2017
10-methyldodec-2-en-4-olide	lactone	21778198	N	Bacteria	*Aeromicrobium marinum*	25 µg (12 mM) = 8 mm of inhibition zone	Dickschat et al., 2005
10-methyldodec-2-en-4-olide	lactone	21778198	N	Bacteria	*Mesorhizobium tianshanense*	25 µg (12 mM) = 9 mm of inhibition zone	Dickschat et al., 2005
10-methyldodec-2-en-4-olide	lactone	21778198	N	Bacteria	*Tenacibaculum mesophilum*	25 µg (12 mM) = +40 mm of inhibition zone	Dickschat et al., 2005
10-methyldodec-2-en-4-olide	lactone	21778198	N	Bacteria	*Zobellia russellii*	25 µg (12 mM) = 24 mm of inhibition zone	Dickschat et al., 2005
1-butanamine	amine / nitrogen compound	8007	Y	Bacteria	*Pochonia chlamydosporia*	spore: 21 mg/L ≥ 99% mycelium: 44 mg/L ≥ 99%	Zou et al., 2007
1-butanamine	amine / nitrogen compound	8007	Y	Bacteria	*Paecilomyces lilacinus*	spore: 20 mg/L ≥ 99% mycelium: 32 mg/L	Zou et al., 2007
1-decanol	alcohol	8174	Y	Bacteria	*Fusarium oxysporum* f. sp. *niveum*	200 µL (concentration N.M.) = 40%	Raza et al., 2015
1-decene	alkene	13381	Y	Bacteria	*Po. chlamydosporia*	spore: 6 mg/L ≥ 99% mycelium: 37 mg/L ≥ 99%	Zou et al., 2007
1-decene	alkene	13381	Y	Bacteria	*Pa. lilacinus*	spore: 6 mg/L ≥ 99% mycelium: 22 mg/L ≥ 99%	Zou et al., 2007

Compounds	Chemical classes	PubChem ID	mVOC*	Identified in	phytopathogen inhibited	Inhibitory concentrations	References
1-dodecanol	alcohol	8193	Y	Bacteria	*F. oxysporum* f. sp. *niveum*	200 µL (concentration N.M.) = 30%	Raza et al., 2015
1-hexadecanol	alcohol	2682	Y	Bacteria	*Bursaphelenchus xylophilus*	2.0 mmol = 9%	Gu et al., 2007
1-hexadecanol	alcohol	2682	Y	Bacteria	*Colletotrichum gloeosporioides*	200 µL (200 µL/mL liquid or 100 mg/mL solid compounds) = 35%	Rajaofera et al., 2019
1-methyl-naphthalene	benzenoid	7002	Y	Bacteria	*F. oxysporum* f. sp. *niveum*	200 µL (concentration N.M.) = 95%	Raza et al., 2015
1-methyl-naphthalene	benzenoid	7002	Y	Bacteria	*Ra. solanacearum*	40 µg/100µL (methanol) = ±50%	Raza et al., 2015
1-octanol	alcohol	957	Y	Bacteria	*Monilinia fructicola*	EC50 = 4.77 x 10^{-5} mol/L	Gao et al., 2018
1-octen-3-ol	alcohol / alkene	18827	Y	Bacteria	*Alternaria brassicae*	7.5 mg = mycelia reduced in 45.2 mm (±65%)	Zhao et al., 2011
1-octen-3-ol	alcohol / alkene	18827	Y	Bacteria	*Bo. cinerea*	5.0 mg = mycelia reduced in 64.4 mm (±90%)	Zhao et al., 2011
1-octen-3-ol	alcohol / alkene	18827	Y	Bacteria	*Colletotrichum capsici*	7.5 mg = mycelia reduced in 38.5 mm (±59%)	Zhao et al., 2011
1-octen-3-ol	alcohol / alkene	18827	Y	Bacteria	*F. oxysporum*	7.5 mg = mycelia reduced in 46.3 mm (±69%)	Zhao et al., 2011
1-octen-3-ol	alcohol / alkene	18827	Y	Bacteria	*Phytophthora capsici*	7.5 mg = mycelia reduced in 53.3 mm (±79%)	Zhao et al., 2011
1-octen-3-ol	alcohol / alkene	18827	Y	Bacteria	*Phytophthora melonis*	7.5 mg = mycelia reduced in 59 mm (±89%)	Zhao et al., 2011
1-octen-3-ol	alcohol / alkene	18827	Y	Bacteria	*Pestalotiopsis photiniae*	7.5 mg = mycelia reduced in 59.2 mm (±85%)	Zhao et al., 2011
1-octen-3-ol	alcohol / alkene	18827	Y	Bacteria	*Rh. solani*	7.5 mg = mycelia reduced in 68.4 mm (±91%)	Zhao et al., 2011

Compounds	Chemical classes	PubChem ID	mVOC*	Identified in	phytopathogen inhibited	Inhibitory concentrations	References
1-octen-3-ol	alcohol / alkene	18827	Y	Fungi	*Bo. cinerea*	10uL (0.1M) = ± 2mm (reduction of lesion size)	Kishimoto et al., 2007
1-undecanol	alcohol	8184	Y	Bacteria	*Ra. solanacearum*	40 µg/100µL (methanol) = ±50%	Raza et al., 2016a
1-undecene	alkene	13190	Y	Bacteria	*Thielaviopsis ethacetica*	5 mM = 100%	Freitas et al., 2022
2,3,5-trimethyl pyrazine	pyrazine / nitrogen compound / heterocyclic compound	26808	Y	Bacteria	*Fusarium solani*	200 µL (standard) = 100%	Guevara-Avendaño et al., 2019
2,3-butanediol	alcohol	262	Y	Bacteria	*Erwinia carotovora* subsp. *carotovora*	0.2 pg – 20 µg = reduced symptomatic leaves in *Arabidopsis*	Ryu et al., 2004
2,4-di-tert-butyl phenol	phenol	7311	N	Bacteria	*Alternaria solani*	80 µL (standard) = 89.14%	Gao et al., 2017
2,4-di-tert-butyl phenol	phenol	7311	N	Bacteria	*Bo. cinerea*	80 µL (standard) = 81.18%	Gao et al., 2017
2,4-di-tert-butyl phenol	alcohol / benzenoid	7311	Y	Bacteria	*Co. gloeosporioides*	EC50 = 1.26×10^{-2} mol/L	Gao et al., 2018
2,4-di-tert-butyl phenol	alcohol / benzenoid	7311	Y	Bacteria	*Mo. fructicola*	EC50 = 9.9×10^{-4} mol/L	Gao et al., 2018
2,5-dimethyl pyrazine	pyrazine / nitrogen compound / heterocyclic compound	31252	Y	Bacteria	*Athelia rolfsii*	EC95 = 400.2 µg/cm^3	Agisha et al., 2019
2,5-dimethyl pyrazine	pyrazine / nitrogen compound / heterocyclic compound	31252	Y	Bacteria	*Al. solani*	80 µL (standard) = 87.5%	Gao et al., 2017
2,5-dimethyl pyrazine	pyrazine / nitrogen compound / heterocyclic compound	31252	Y	Bacteria	*Bo. cinerea*	80 µL (standard) = 100%	Gao et al., 2017
2,5-dimethyl pyrazine	pyrazine / nitrogen compound / heterocyclic compound	31252	Y	Bacteria	*Co. gloeosporioides*	EC95 = 571.0 µg/cm^3	Agisha et al., 2019
2,5-dimethyl pyrazine	pyrazine / nitrogen compound / heterocyclic compound	31252	Y	Bacteria	*Giberella moliniformis*	EC95 = 1058 µg/cm^3	Agisha et al., 2019

Compounds	Chemical classes	PubChem ID	mVOC*	Identified in	phytopathogen inhibited	Inhibitory concentrations	References
2,5-dimethyl pyrazine	pyrazine / nitrogen compound / heterocyclic compound	31252	Y	Bacteria	*Magnaporthe oryzae*	EC95 = 32217 µg/cm^3	Agisha et al., 2019
2,5-dimethyl pyrazine	pyrazine / nitrogen compound / heterocyclic compound	31252	Y	Bacteria	*Ma. oryzae*	1 mL (672 µg/mL) = 74%	Munjal et al., 2016
2,5-dimethyl pyrazine	pyrazine / nitrogen compound / heterocyclic compound	31252	Y	Bacteria	*Phyt. capsici*	EC95 = 748.1 µg/cm^3	Agisha et al., 2019
2,5-dimethyl pyrazine	pyrazine / nitrogen compound / heterocyclic compound	31252	Y	Bacteria	*Phyt. capsici*	1 mL (504 µg/mL) = 100%	Munjal et al., 2016
2,5-dimethyl pyrazine	pyrazine / nitrogen compound / heterocyclic compound	31252	Y	Bacteria	*Per. litchii*	EC50 = 744.5 mg/L	Zheng et al., 2019a
2,5-dimethyl pyrazine	pyrazine / nitrogen compound / heterocyclic compound	31252	Y	Bacteria	*Pythium myriotilum*	EC95 = 185.0 µg/cm^3	Agisha et al., 2019
2,5-dimethyl pyrazine	pyrazine / nitrogen compound / heterocyclic compound	31252	Y	Bacteria	*Pythium ultimum*	10 mg > 50%	Vlassi et al., 2020
2,5-dimethyl pyrazine	pyrazine / nitrogen compound / heterocyclic compound	31252	Y	Bacteria	*Ra. pseudosolanacearum*	679 µg/cm^3 = 80%	Agisha et al., 2019
2,5-dimethyl pyrazine	pyrazine / nitrogen compound / heterocyclic compound	31252	Y	Bacteria	*Ra. solanacearum*	1 mL (672 µg/mL) = 80%	Munjal et al., 2016
2,5-dimethyl pyrazine	pyrazine / nitrogen compound / heterocyclic compound	31252	Y	Bacteria	*Rh. solani*	EC95 = 219.3 µg/cm^3	Agisha et al., 2019
2,5-dimethyl pyrazine	pyrazine / nitrogen compound / heterocyclic compound	31252	Y	Bacteria	*Rh. solani*	10 mg > 50%	Vlassi et al., 2020
2,5-dimethyl pyrazine	pyrazine / nitrogen compound / heterocyclic compound	31252	Y	Bacteria	*Sclerotinia sclerotiorum*	10 mg ≈ 80%	Vlassi et al., 2020
2-decanol	alcohol	14254	Y	Bacteria	*Ra. solanacearum*	40 µg/100µL (methanol) = ±50%	Raza et al., 2016a

Compounds	Chemical classes	PubChem ID	mVOC*	Identified in	phytopathogen inhibited	Inhibitory concentrations	References
2-decanone	ketone	12741	Y	Bacteria	*Colletotrichum acutatum*	80 µL (standard) = 61%	Che et al., 2017
2-decanone	ketone	12741	Y	Bacteria	*F. solani*	200 µL (standard) = 100%	Guevara-Avendaño et al., 2019
2-dodecanone	ketone	22556	Y	Bacteria	*F. solani*	200 µL (standard) = 38%	Guevara-Avendaño et al., 2019
2-ethyl pyrazine	pyrazine	26331	N	Bacteria	*Ma. oryzae*	1 mL (672 µg/mL) = 100%	Munjal et al., 2016
2-ethyl pyrazine	pyrazine	26331	N	Bacteria	*Phyt. capsici*	1 mL (504 µg/mL) = 100%	Munjal et al., 2016
2-ethyl pyrazine	pyrazine	26331	N	Bacteria	*Ra. solanacearum*	1 mL (672 µg/mL) = 55.12%	Munjal et al., 2016
2-ethyl-3,6-dimethyl pyrazine	pyrazine / nitrogen compound	25916	Y	Bacteria	*At. rolfsii*	EC95 = 85.0 µg/cm^3	Agisha et al., 2019
2-ethyl-3,6-dimethyl pyrazine	pyrazine / nitrogen compound	25916	Y	Bacteria	*Co. gloeosporioides*	EC95 = 108.0 µg/cm^3	Agisha et al., 2019
2-ethyl-3,6-dimethyl pyrazine	pyrazine / nitrogen compound	25916	Y	Bacteria	*G. moliniformis*	EC95 = 137.3 µg/cm^3	Agisha et al., 2019
2-ethyl-3,6-dimethyl pyrazine	pyrazine / nitrogen compound	25916	Y	Bacteria	*Ma. oryzae*	EC95 = 937.9 µg/cm^3	Agisha et al., 2019
2-ethyl-3,6-dimethyl pyrazine	pyrazine / nitrogen compound	25916	Y	Bacteria	*Phyt. capsici*	EC95 = 382.1 µg/cm^3	Agisha et al., 2019
2-ethyl-3,6-dimethyl pyrazine	pyrazine / nitrogen compound	25916	Y	Bacteria	*Py. myriotilum*	EC95 = 78.2 µg/cm^3	Agisha et al., 2019
2-ethyl-3,6-dimethyl pyrazine	pyrazine / nitrogen compound	25916	Y	Bacteria	*Ra. pseudosolanacearum*	679 µg/cm^3 = 93%	Agisha et al., 2019
2-ethyl-3,6-dimethyl pyrazine	pyrazine / nitrogen compound	25916	Y	Bacteria	*Rh. solani*	EC95 = 49.5 µg/cm^3	Agisha et al., 2019
2-ethyl-3-methoxy pyrazine	pyrazine / nitrogen compound	33135	**	Bacteria	*Py. ultimum*	10 mg > 50%	Vlassi et al., 2020
2-ethyl-3-methoxy pyrazine	pyrazine / nitrogen compound	33135	**	Bacteria	*Rh. solani*	10 mg > 50%	Vlassi et al., 2020
2-ethyl-3-methoxy pyrazine	pyrazine / nitrogen compound	33135	**	Bacteria	*S. sclerotiorum*	10 mg ≈ 90%	Vlassi et al., 2020

Compounds	Chemical classes	PubChem ID	mVOC*	Identified in	phytopathogen inhibited	Inhibitory concentrations	References
2-ethyl-3-methyl pyrazine	pyrazine	27457	N	Bacteria	*Ma. oryzae*	1 mL (168 µg/mL) = 100%	Munjal et al., 2016
2-ethyl-3-methyl pyrazine	pyrazine	27457	N	Bacteria	*Meloidogyne* sp.	20 µL (standard) = 50%	Wolfgang et al., 2019
2-ethyl-3-methyl pyrazine	pyrazine	27457	N	Bacteria	*Phyt. capsici*	1 mL (168 µg/mL) = 100%	Munjal et al., 2016
2-ethyl-3-methyl pyrazine	pyrazine	27457	N	Bacteria	*Ra. solanacearum*	1 mL (672 µg/mL) = 96%	Munjal et al., 2016
2-ethyl-5-methyl pyrazine	pyrazine / nitrogen compound / heterocyclic compound	25915	Y	Bacteria	*At. rolfsii*	EC95 = 145.3 µg/cm^3	Agisha et al., 2019
2-ethyl-5-methyl pyrazine	pyrazine / nitrogen compound / heterocyclic compound	25915	Y	Bacteria	*Co. gloeosporioides*	EC95 = 232.2 µg/cm^3	Agisha et al., 2019
2-ethyl-5-methyl pyrazine	pyrazine / nitrogen compound / heterocyclic compound	25915	Y	Bacteria	*G. moliniformis*	EC95 = 290.4 µg/cm^3	Agisha et al., 2019
2-ethyl-5-methyl pyrazine	pyrazine / nitrogen compound / heterocyclic compound	25915	Y	Bacteria	*Ma. oryzae*	EC95 = 937.9 µg/cm^3	Agisha et al., 2019
2-ethyl-5-methyl pyrazine	pyrazine / nitrogen compound / heterocyclic compound	25915	Y	Bacteria	*Phyt. capsici*	EC95 = 491.0 µg/cm^3	Agisha et al., 2019
2-ethyl-5-methyl pyrazine	pyrazine / nitrogen compound / heterocyclic compound	25915	Y	Bacteria	*Py. myriotilum*	EC95 = 95.0 µg/cm^3	Agisha et al., 2019
2-ethyl-5-methyl pyrazine	pyrazine / nitrogen compound / heterocyclic compound	25915	Y	Bacteria	*Ra. pseudosolanacearum*	679 µg/cm^3 = 83%	Agisha et al., 2019
2-ethyl-5-methyl pyrazine	pyrazine / nitrogen compound / heterocyclic compound	25915	Y	Bacteria	*Rh. solani*	EC95 = 75.3 µg/cm^3	Agisha et al., 2019
2-ethylhexan-1-ol	alcohol	7720	Y	Bacteria	*Co. acutatum*	10 µL (standard) = 100%	Che et al., 2017
2-ethylhexan-1-ol	alcohol	7720	Y	Bacteria	*Per. litchii*	EC50 = 180.1 mg/L	Zheng et al., 2019a

Compounds	Chemical classes	PubChem ID	mVOC*	Identified in	phytopathogen inhibited	Inhibitory concentrations	References
2-ethylhexan-1-ol	alcohol	7720	Y	Bacteria	*Phyt. capsici*	10 µg/mL (volume N.M.) = ± 60% inhibition	Syed-Ab-Rahman et al., 2019
2-ethylhexan-1-ol	alcohol	7720	Y	Bacteria	*S. sclerotiorum*	100 and 150 µL = 100%	Fernando et al., 2005
2-heptanone	ketone	8051	Y	Bacteria	*Agrobacterium tumefasciens*	100 µmol = 100%	Popova et al., 2014
2-heptanone	ketone	8051	Y	Bacteria	*Ag. tumefaciens*	75 µmol = 100%	Plyuta et al., 2016
2-heptanone	ketone	8051	Y	Bacteria	*Phyt. capsici*	10 µg/mL (volume N.M.) = ± 60%	Syed-Ab-Rahman et al., 2019
2-heptanone	ketone	8051	Y	Bacteria	*Synechococcus* sp.	100 µmol = 100%	Popova et al., 2014
2-hexadecanol	alcohol	85779	N	Bacteria	*F. oxysporum* f. sp. *niveum*	200 µL (concentration N.M.) = 60%	Raza et al., 2015
2-isobutyl-3-methyl pyrazine	pyrazine	26333	N	Bacteria	*Meloidogyne* sp.	20 µL (standard) = 68%	Wolfgang et al., 2019
2-isopropyl-3-methoxy pyrazine	pyrazine	33166	**	Bacteria	*Py. ultimum*	10 mg > 40%	Vlassi et al., 2020
2-isopropyl-3-methoxy pyrazine	pyrazine	33166	**	Bacteria	*Rh. solani*	7.5 mg > 10%	Vlassi et al., 2020
2-isopropyl-3-methoxy pyrazine	pyrazine	33166	**	Bacteria	*S. sclerotiorum*	10 mg ≈ 90%	Vlassi et al., 2020
2-methoxy-3-methyl pyrazine	pyrazine	17898	N	Bacteria	*Meloidogyne* sp.	175 nmol = 76%	Wolfgang et al., 2019
2-methyl butyric acid	carboxylic acid / ester	8314	Y	Bacteria	*Colletotrichum lindemuthianum*	10 µL (102.3 g/mol) = up to 94% inhibition	Martins et al., 2019
2-methyl pyrazine	pyrazine / nitrogen compound	7976	Y	Bacteria	*At. rolfsii*	EC95 = 578.3 µg/cm^3	Agisha et al., 2019
2-methyl pyrazine	pyrazine / nitrogen compound	7976	Y	Bacteria	*Co. gloeosporioides*	EC95 = 519.2 µg/cm^3	Agisha et al., 2019
2-methyl pyrazine	pyrazine / nitrogen compound	7976	Y	Bacteria	*G. moliniformis*	EC95 = 1624 µg/cm^3	Agisha et al., 2019
2-methyl pyrazine	pyrazine / nitrogen compound	7976	Y	Bacteria	*Ma. oryzae*	1 mL (672 µg/mL) = 100%	Munjal et al., 2016

Compounds	Chemical classes	PubChem ID	mVOC*	Identified in	phytopathogen inhibited	Inhibitory concentrations	References
2-methyl pyrazine	pyrazine / nitrogen compound	7976	Y	Bacteria	*Ma. oryzae*	EC95 = 1802 µg/cm^3	Agisha et al., 2019
2-methyl pyrazine	pyrazine / nitrogen compound	7976	Y	Bacteria	*Phyt. capsici*	EC95 = 1571 µg/cm^3	Agisha et al., 2019
2-methyl pyrazine	pyrazine / nitrogen compound	7976	Y	Bacteria	*Phyt. capsici*	1 mL (672 µg/mL) = 100%	Munjal et al., 2016
2-methyl pyrazine	pyrazine / nitrogen compound	7976	Y	Bacteria	*Py. myriotilum*	EC95 = 361.1 µg/cm^3	Agisha et al., 2019
2-methyl pyrazine	pyrazine / nitrogen compound	7976	Y	Bacteria	*Ra. solanacearum*	1 mL (672 µg/mL) = 32%	Munjal et al., 2016
2-methyl pyrazine	pyrazine / nitrogen compound	7976	Y	Bacteria	*Rh. solani*	EC95 = 207.8 µg/cm^3	Agisha et al., 2019
2-methyl pyrazine	pyrazine / nitrogen compound	7976	Y	Bacteria	*Ra. pseudosolanacearum*	679 µg/cm^3 = 32%	Agisha et al., 2019
2-methyl-1-butanol	alcohol	8723	Y	Fungi	*Bo. cinerea*	EC50 = 1.38 µL/mL	Di Francesco et al., 2015
2-methyl-1-butanol	alcohol	8723	Y	Fungi	*Co. acutatum*	EC50 = 1.27 µL/mL	Di Francesco et al., 2015
2-methyl-1-butanol	alcohol	8723	Y	Fungi	*F. oxysporum*	1000 µg/mL = ± 20%	Medina-Romero et al., 2017
2-methyl-1-butanol	alcohol	8723	Y	Bacteria	*F. oxysporum* f. sp. *niveum*	200 µL (concentration N.M.) = 100%	Raza et al., 2015
2-methyl-1-butanol	alcohol	8723	Y	Fungi	*Mo. fructicola*	EC50 = 0.017 µL/mL	Di Francesco et al., 2020
2-methyl-1-butanol	alcohol	8723	Y	Fungi	*Monilinia fructigena*	EC50 = 0.014 µL/mL	Di Francesco et al., 2020
2-methyl-1-butanol	alcohol	8723	Y	Fungi	*Monilinia laxa*	EC50 = 0.012 µL/mL	Di Francesco et al., 2020
2-methyl-1-butanol	alcohol	8723	Y	Fungi	*Monilinia. polystroma*	EC50 = 0.011 µL/mL	Di Francesco et al., 2020
2-methyl-1-butanol	alcohol	8723	Y	Fungi	*Phyllosticta citricarpa*	50 µL (concentration N.M.) = 100%	Toffano et al., 2017
2-methyl-1-butanol	alcohol	8723	Y	Fungi	*Phytophthora crispans*	EC50 = 3.67 µL/L	Di Francesco et al., 2017
2-methyl-1-butanol	alcohol	8723	Y	Fungi	*Penicillium digitatum*	EC50 = 0.48 µL/mL	Di Francesco et al., 2015

Compounds	Chemical classes	PubChem ID	mVOC*	Identified in	phytopathogen inhibited	Inhibitory concentrations	References
2-methyl-1-butanol	alcohol	8723	Y	Fungi	*Penicillium expansum*	EC50 = 0.89 µL/mL	Di Francesco et al., 2015
2-methyl-1-butanol	alcohol	8723	Y	Fungi	*Penicillium italicum*	EC50 = 0.85 µL/mL	Di Francesco et al., 2015
2-methyl-1-propanol	alcohol	6560	Y	Fungi	*Bo. cinerea*	EC50 = 0.82 µL/mL	Di Francesco et al., 2015
2-methyl-1-propanol	alcohol	6560	Y	Fungi	*Co. acutatum*	EC50 = 1.54 µL/mL	Di Francesco et al., 2015
2-methyl-1-propanol	alcohol	6560	Y	Fungi	*Mo. fructicola*	EC50 = 0.021 µL/mL	Di Francesco et al., 2020
2-methyl-1-propanol	alcohol	6560	Y	Fungi	*Mo. fructigena*	EC50 = 0.127 µL/mL	Di Francesco et al., 2020
2-methyl-1-propanol	alcohol	6560	Y	Fungi	*Mo. laxa*	EC50 = 0.019 µL/mL	Di Francesco et al., 2020
2-methyl-1-propanol	alcohol	6560	Y	Fungi	*Mo. polystroma*	EC50 = 0.019 µL/mL	Di Francesco et al., 2020
2-methyl-1-propanol	alcohol	6560	Y	Fungi	*Phyt. crispans*	EC50 = 4.51 µL/L	Di Francesco et al., 2017
2-methyl-1-propanol	alcohol	6560	Y	Fungi	*Pen. digitatum*	EC50 = 1.71 µL/mL	Di Francesco et al., 2015
2-methyl-1-propanol	alcohol	6560	Y	Fungi	*Pen. expansum*	EC50 = 1.28 µL/mL	Di Francesco et al., 2015
2-methyl-1-propanol	alcohol	6560	Y	Fungi	*Pen. italicum*	EC50 = 1.65 µL/mL	Di Francesco et al., 2015
2-methyl-1-propanol	alcohol	6560	Y	Bacteria	*T. ethacetica*	5 mM = 100%	Freitas et al., 2022
2-nonanol	alcohol	12367	Y	Bacteria	*T. ethacetica*	0.5 mM = 100%	Freitas et al., 2022
2-nonanone	ketone	13187	Y	Bacteria	*Ag. tumefaciens*	20 µmol = 100%	Plyuta et al., 2016
2-nonanone	ketone	13187	Y	Bacteria	*Ag. tumefaciens*	100 µmol = 100%	Popova et al., 2014
2-nonanone	ketone	13187	Y	Fungi	*Bo. cinerea*	125 µL/L = 100%	Guo et al., 2019
2-nonanone	ketone	13187	Y	Bacteria	*Burs. xylophilus*	0.40 mmol = 100%	Gu et al., 2007

**Compounds**	**Chemical classes**	**PubChem ID**	**mVOC***	**Identified in**	**phytopathogen inhibited**	**Inhibitory concentrations**	**References**
2-nonanone	ketone	13187	Y	Bacteria	*F. oxysporum* f. sp. *niveum*	200 µL (concentration N.M.) = 100%	Raza et al., 2015
2-nonanone	ketone	13187	Y	Bacteria	*F. solani*	200 µL (standard) = 100%	Guevara-Avendaño et al., 2019
2-nonanone	ketone	13187	Y	Bacteria	*Rh. solani*	100 µmol = 100%	Popova et al., 2014
2-nonanone	ketone	13187	Y	Bacteria	*S. sclerotiorum*	mycelium MIQ = 4.92 mg<br>sclerotia MIQ = 16.4 mg	Giorgio et al., 2015
2-nonanone	ketone	13187	Y	Bacteria	*Synechococcus* sp.	100 µmol = 100%	Popova et al., 2014
2-nonanone	ketone	13187	Y	Bacteria	*T. ethacetica*	0.5 mM = 100%	Freitas et al., 2022
2-octanol	alcohol	20083	Y	Bacteria	*Burs. xylophilus*	0.06 mmol = 100%	Gu et al., 2007
2-pentylfuran	furan / ether / heterocyclic compound	19602	Y	Bacteria	*F. oxysporum* f. sp. *cucumerinum*	80 µL (standard) = 25%	Wu et al., 2015
2-pentylfuran	furan / ether / heterocyclic compound	19602	Y	Bacteria	*S. sclerotiorum*	80 µL (standard) = 77%	Wu et al., 2015
2-phenethylethanol (phenylethyl alcohol)	benzenoid / alcohol	6054	Y	Fungi	*F. oxysporum*	1000 µg/mL = ± 50%	Medina-Romero et al., 2017
2-phenethylethanol	benzenoid / alcohol	6054	Y	Not identified	*Aspergillus flavus*	1000 µL/L = ±55%<br>There are also results with conidial germination (*in vitro*), sporulation (*in vitro* and *in vivo*), and spoilage control by fumigation	Boukaew and Prasertsan, 2018
2-phenethylethanol	benzenoid / alcohol	6054	Y	Not identified	*Aspergillus parasiticus*	1000 µL/L = ±30%<br>There are also results with conidial germination (*in vitro*), sporulation (*in vitro* and *in vivo*), and spoilage control by fumigation	Boukaew and Prasertsan, 2018

Compounds	Chemical classes	PubChem ID	mVOC*	Identified in	phytopathogen inhibited	Inhibitory concentrations	References
2-phenethylethanol	benzenoid / alcohol	6054	Y	Fungi	*Bo. cinerea*	EC50 = 0.57 µL/mL	Di Francesco et al., 2015
2-phenethylethanol	benzenoid / alcohol	6054	Y	Fungi	*Bo. cinerea*	625 µL/L = 100%	Guo et al., 2019
2-phenethylethanol	benzenoid / alcohol	6054	Y	Fungi	*Co. acutatum*	EC50 = 1.97 µL/mL	Di Francesco et al., 2015
2-phenethylethanol	benzenoid / alcohol	6054	Y	Fungi	*Co. gloeosporioides*	EC50 = 1.99 µL/mL	Zhou et al., 2018
2-phenethylethanol	benzenoid / alcohol	6054	Y	Fungi	*Mo. fructicola*	0.56 µL/mL = 100%	Di Francesco et al., 2020
2-phenethylethanol	benzenoid / alcohol	6054	Y	Fungi	*Mo. laxa*	0.56 µL/mL = 100%	Di Francesco et al., 2020
2-phenethylethanol	benzenoid / alcohol	6054	Y	Fungi	*Mo. fructigena*	0.56 µL/mL = 100%	Di Francesco et al., 2020
2-phenethylethanol	benzenoid / alcohol	6054	Y	Fungi	*Mo. polystroma*	0.56 µL/mL = 100%	Di Francesco et al., 2020
2-phenethylethanol	benzenoid / alcohol	6054	Y	Bacteria	*Per. litchii*	1000 µL/L (volume N.M.) = mycelia reduced in 12.9 mm (±25%)	Xing et al., 2018
2-phenethylethanol	benzenoid / alcohol	6054	Y	Fungi	*Pen. digitatum*	EC50 = 0.61 µL/mL	Di Francesco et al., 2015
2-phenethylethanol	benzenoid / alcohol	6054	Y	Fungi	*Pen. expansum*	EC50 = 0.79 µL/mL	Di Francesco et al., 2015
2-phenethylethanol	benzenoid / alcohol	6054	Y	Fungi	*Pen. italicum*	EC50 = 0.62 µL/mL	Di Francesco et al., 2015
2-phenethylethanol	alcohol / benzenoid	6054	Y	Fungi	*Phyl. citricarpa*	50 µL (concentration N.M.) = 35%	Toffano et al., 2017
2-phenethylethanol	benzenoid / alcohol	6054	Y	Fungi	*Phyt. crispans*	EC50 = 3.67 µL/L	Di Francesco et al., 2017
2-phenethylethanol	benzenoid / alcohol	6054	Y	Bacteria	*T. ethacetica*	50 mM = 100%	Freitas et al., 2022
2R, 3R-butanediol	alcohol	262	Y	Bacteria	*E. carotovora* subsp. *carotovora*	100 µg = 11% (D.I.)	Han et al., 2006
2-tridecanol	alcohol	15449	Y	Bacteria	*Ra. solanacearum*	40 µg/100µL (methanol) = ±50%	Raza et al., 2016a

Compounds	Chemical classes	PubChem ID	mVOC*	Identified in	phytopathogen inhibited	Inhibitory concentrations	References
2-tridecanone	ketone	11622	Y	Bacteria	*F. oxysporum* f. sp. *niveum*	200 µL (concentration N.M.) = 70%	Raza et al., 2015
2-tridecanone	ketone	11622	Y	Bacteria	*T. ethacetica*	0.5 mM = 100 %	Freitas et al., 2022
2-undecanol	alcohol		Y	Bacteria	*T. ethacetica*	0.5 mM = 100%	Freitas et al., 2022
2-undecanone	ketone	8163	Y	Bacteria	*Ag. tumefaciens*	100 µmol = 80%	Plyuta et al., 2016
2-undecanone	ketone	8163	Y	Bacteria	*Burs. xylophilus*	0.05 mmol = 100%	Gu et al., 2007
2-undecanone	ketone	8163	Y	Bacteria	*Meloidogyne* sp.	100 nmol = 95%	Wolfgang et al., 2019
2-undecanone	ketone	8163	Y	Bacteria	*Per. litchii*	EC50 = 764.3 mg/L	Zheng et al., 2019a
2-undecanone	ketone	8163	Y	Bacteria	*S. sclerotiorum*	mycelium MIQ = 14.85 mg<br>sclerotia MIQ = 16.5 mg	Giorgio et al., 2015
2-undecanone	ketone	8163	Y	Bacteria	*Synechococcus* sp.	100 µmol = 100%	Popova et al., 2014
3-methyl-1-butanol	alcohol	31260	Y	Fungi	*Bo. cinerea*	EC50 = 0.78 µL/mL	Di Francesco et al., 2015
3-methyl-1-butanol	alcohol	31260	Y	Fungi	*Bo. cinerea*	1250 µL/L = 100%	Guo et al., 2019
3-methyl-1-butanol	alcohol	31260	Y	Fungi	*Co. acutatum*	EC50 = 1.35 µL/mL	Di Francesco et al., 2015
3-methyl-1-butanol	alcohol	31260	Y	Fungi	*F. oxysporum*	1000 µg/mL = ± 20%	Medina-Romero et al., 2017
3-methyl-1-butanol	alcohol	31260	Y	Fungi	*M.o fructicola*	EC50 = 0.015 µL/mL	Di Francesco et al., 2020
3-methyl-1-butanol	alcohol	31260	Y	Fungi	*Mo. fructigena*	EC50 = 0.013 µL/mL	Di Francesco et al., 2020
3-methyl-1-butanol	alcohol	31260	Y	Fungi	*Mo. laxa*	EC50 = 0.012 µL/mL	Di Francesco et al., 2020
3-methyl-1-butanol	alcohol	31260	Y	Fungi	*Mo. polystroma*	EC50 = 0.010 µL/mL	Di Francesco et al., 2020

Compounds	Chemical classes	PubChem ID	mVOC*	Identified in	phytopathogen inhibited	Inhibitory concentrations	References
3-methyl-1-butanol	alcohol	31260	Y	Fungi	*Phyt. crispans*	EC50 = 3.62 µL/L	Di Francesco et al., 2017
3-methyl-1-butanol	alcohol	31260	Y	Fungi	*Pen. digitatum*	EC50 = 0.73 µL/mL	Di Francesco et al., 2015
3-methyl-1-butanol	alcohol	31260	Y	Fungi	*Pen. expansum*	EC50 = 1.01 µL/mL	Di Francesco et al., 2015
3-methyl-1-butanol	alcohol	31260	Y	Fungi	*Pen. italicum*	EC50 = 1.13 µL/mL	Di Francesco et al., 2015
3-methyl-1-butanol	alcohol	31260	Y	Bacteria	*Phyt. capsici*	10 µg/mL (volume N.M.) = ± 65% inhibition	Syed-Ab-Rahman et al., 2019
3-methylbutanal	aldehyde	11552	Y	Bacteria	*Co. gloeosporioides*	EC50 = 7.67 x 10^{-3} mol/L	Gao et al., 2018
3-methylbutanal	aldehyde	11552	Y	Bacteria	*Phyt. capsici*	10 µg/mL (volume N.M.) = 100% inhibition	Syed-Ab-Rahman et al., 2019
3-phenyl-1-propanol	alcohol / benzenoid	31234	Y	Fungi	*Bo. cinerea*	1250 µL/L = 75%	Guo et al., 2019
4-chloro-3-methyl phenol	phenol	1732	N	Bacteria	*Al. solani*	80 µL (standard) = 100%	Gao et al., 2017
4-chloro-3-methyl phenol	phenol	1732	N	Bacteria	*Bo. cinerea*	80 µL (standard) = 100%	Gao et al., 2017
4-ethylphenol	phenol	31242	N	Fungi	*Bo. cinerea*	100 mg/L = 89%	Guo et al., 2019
4-ethylphenol	phenol	31242	N	Bacteria	*Per. litchii*	1000 µL/L (volume N.M.) = 100%	Xing et al., 2018
4-methoxystyrene	benzenoid / alkene / ether	12507	Y	Bacteria	*F. oxysporum* f. sp. *cucumerinum*	80 µL (standard) = 46%	Wu et al., 2015
4-methoxystyrene	benzenoid / alkene / ether	12507	Y	Bacteria	*S. sclerotiorum*	80 µL (standard) = 91%	Wu et al., 2015
5-isobutyl-2,3-dimethyl pyrazine	pyrazine	528108	N	Bacteria	*Meloidogyne* sp.	20 µL (standard) = 44%	Wolfgang et al., 2019
5-(4-pentenyl)-2-furaldehyde	furan / aldehyde	86008063	**	Bacteria	*Colletotrichum fragariae*	N.I. (1:10 dilution of pure compound) = 100%	Koitabashi et al., 2004
5-pentyl-2-furaldehyde	furan / aldehyde	68532-62-7	**	Bacteria	*Bo. cinerea*	8 µg/mL = 100%	Koitabashi et al., 2004

Compounds	Chemical classes	PubChem ID	mVOC*	Identified in	phytopathogen inhibited	Inhibitory concentrations	References
5-pentyl-2-furaldehyde	furan / aldehyde	68532-62-7	**	Bacteria	*Co. fragariae*	8 µg/mL = 100%	Koitabashi et al., 2004
5-pentyl-2-furaldehyde	furan / aldehyde	68532-62-7	**	Bacteria	*F. oxysporum*	2 µg/mL = 100%	Koitabashi et al., 2004
acetamide	amide / nitrogen compound	178	Y	Bacteria	*Pa. lilacinus*	spore: 2 mg/L ≥ 99% mycelium: 53 mg/L ≥ 99%	Zou et al., 2007
acetamide	amide / nitrogen compound	178	Y	Bacteria	*Po. chlamydosporia*	spore: 4 mg/L ≥ 99% mycelium: 67 mg/L ≥ 99%	Zou et al., 2007
acetic acid	acid / carboxylic acid	176	Y	Bacteria	*S.. sclerotiorum*	mycelium MIQ = 4.19 mg sclerotia MIQ = 9.44 mg	Giorgio et al., 2015
acetoin	ketone / alcohol	179	Y	Bacteria	*Bo. cinerea*	1.35 µL/mL = 47%	Gotor-Vila et al., 2017
acetoin	ketone / alcohol	179	Y	Bacteria	*Co. lindemuthianum*	10 µL (88.8 g/mol) = partial inhibition (% N.M.)	Martins et al., 2019
acetoin	ketone / alcohol	179	Y	Bacteria	*Mo. laxa*	1.35 µL/mL = 23%	Gotor-Vila et al., 2017
acetophenone	benzenoid / ketone	7410	Y	Not identified	*As. flavus*	100 and 1000 µL/L = 100% There are also results with conidial germination (*in vitro*), sporulation (*in vitro* and *in vivo*), and spoilage control by fumigation	Boukaew and Prasertsan, 2018
acetophenone	ketone	7410	Y	Bacteria	*Burs. xylophilus*	2.0 mmol = 100%	Gu et al., 2007
acetophenone	benzenoid / ketone	7410	Y	Not identified	*As. parasiticus*	100 and 1000 µL/L = 100% There are also results with conidial germination (*in vitro*), sporulation (*in vitro* and	Boukaew and Prasertsan, 2018

Compounds	Chemical classes	PubChem ID	mVOC*	Identified in	phytopathogen inhibited	Inhibitory concentrations	References
						in vivo), and spoilage control by fumigation	
acetophenone	benzenoid / ketone	7410	Y	Bacteria	*F. oxysporum* f. sp. *niveum*	200 µL (concentration N.M.) = 100%	Raza et al., 2015
a-copaene	terpene	70678558	Y	Bacteria	*Per. litchii*	500 µL/L (volume N.M.) = 100%	Xing et al., 2018
ammonia	nitrogen compound	222	N	Bacteria	*Pythium aphanidermatum*	10uL (25 g/mL) = 100%	Huang et al., 2018
ammonia	nitrogen compound	222	N	Bacteria	*Py. ultimum*	8 µL (concentration N.M.) = 100%	Howell, 1988
ammonia	nitrogen compound	222	N	Bacteria	*Rh. solani*	10uL (25 g/mL) = 100%	Huang et al., 2018
ammonia	nitrogen compound	222	N	Bacteria	*Rh. solani*	8 µL (concentration N.M.) = 100%	Howell, 1988
anisole	benzenoid / ether	7519	Y	Bacteria	*Co. gloeosporioides*	EC50 = 0.59 mol/L	Gao et al., 2018
anisole	benzenoid / ether	7519	Y	Bacteria	*F. oxysporum* f. sp. *cucumerinum*	80 µL (standard) = 38%	Wu et al., 2015
anisole	benzenoid / ether	7519	Y	Bacteria	*S. sclerotiorum*	80 µL (standard) = 76%	Wu et al., 2015
benzaldehyde	benzenoid / aldehyde	240	Y	Bacteria	*Burs. xylophilus*	0.39 mmol = 100%	Gu et al., 2007
benzaldehyde	benzenoid / aldehyde	240	Y	Bacteria	*Clavibacter michiganensis* subsp. *sepedonicus*	100 µl (10 and 100 µL/mL and undiluted) = cell viability reduced in up to ±7 log CFU/mL	Rajer et al., 2017
benzaldehyde	benzenoid / aldehyde	240	Y	Bacteria	*Co. acutatum*	40 µL (standard) = 100%	Che et al., 2017
benzaldehyde	benzenoid / aldehyde	240	Y	Bacteria	*F. oxysporum* sp. *niveum*	200 µL (concentration N.M.) = 100%	Raza et al., 2015
benzaldehyde	benzenoid / aldehyde	240	Y	Bacteria	*Mo. fructicola*	EC50 = 6.7 x 10^{-4} mol/L	Gao et al., 2018
benzaldehyde	benzenoid / aldehyde	240	Y	Bacteria	*Po. chlamydosporia*	spore: 6 mg/L ≥ 99% mycelium: 40 mg/L ≥ 99%	Zou et al., 2007

Compounds	Chemical classes	PubChem ID	mVOC*	Identified in	phytopathogen inhibited	Inhibitory concentrations	References
benzaldehyde	benzenoid / aldehyde	240	Y	Bacteria	*Pa. lilacinus*	spore: 4 mg/L ≥ 99% mycelium: 16 mg/L ≥ 99%	Zou et al., 2007
benzaldehyde	benzenoid / aldehyde	240	Y	Bacteria	*Ra. solanacearum*	5.2 mg = ±60%	Tahir et al., 2017a
benzaldehyde	benzenoid / aldehyde	240	Y	Bacteria	*Ra. solanacearum*	40 µg/100µL (methanol) = ±50%	Raza et al., 2016a
benzaldehyde	benzenoid / aldehyde	240	Y	Bacteria	*T. ethacetica*	5 mM = 100%	Freitas et al., 2022
benzeneethanol	alcohol	21965192	N	Bacteria	*Burs. xylophilus*	2.0 mmol = 85%	Gu et al., 2007
benzoic acid	carboxylic acid / benzenoid	243	Y	Bacteria	*Mo. fructicola*	EC50 = 1.46×10^{-3} mol/L	Gao et al., 2018
benzothiazole	benzenoid / thiazole / sulfur compound / nitrogen compound	7222	Y	Bacteria	*Al. solani*	80 µL (standard) = 100%	Gao et al., 2017
benzothiazole	benzenoid / thiazole / sulfur compound / nitrogen compound	7222	Y	Bacteria	*Al. brassicae*	7.5 mg = mycelia reduced in 62.6 mm (±90%)	Zhao et al., 2011
benzothiazole	benzenoid / thiazole / sulfur compound / nitrogen compound	7222	Y	Bacteria	*Bo. cinerea*	80 µL (standard) = 100%	Gao et al., 2017
benzothiazole	benzenoid / thiazole / sulfur compound / nitrogen compound	7222	Y	Bacteria	*Bo. cinerea*	5.0 mg = mycelia reduced in 64.4 mm (±90%)	Zhao et al., 2011
benzothiazole	benzenoid / thiazole / sulfur compound / nitrogen compound	7222	Y	Bacteria	*Co. gloeosporioides*	EC50 = 0.36 mol/L	Gao et al., 2018
benzothiazole	benzenoid / thiazole / sulfur compound / nitrogen compound	7222	Y	Bacteria	*Cl. michiganensis* subsp. *sepedonicus*	100 µl (10 and 100 µL/mL and undiluted) = cell viability reduced in up to ±3 log CFU/mL	Rajer et al., 2017
benzothiazole	benzenoid / thiazole / sulfur compound / nitrogen compound	7222	Y	Bacteria	*Co. capsici*	7.5 mg = mycelia reduced in 58.7 mm (±89%)	Zhao et al., 2011
benzothiazole	benzenoid / thiazole / sulfur compound / nitrogen compound	7222	Y	Bacteria	*F. oxysporum*	7.5 mg = mycelia reduced in 39.7 mm (±60%)	Zhao et al., 2011

Compounds	Chemical classes	PubChem ID	mVOC*	Identified in	phytopathogen inhibited	Inhibitory concentrations	References
benzothiazole	benzenoid / thiazole / sulfur compound / nitrogen compound	7222	Y	Bacteria	*F. oxysporum* f. sp. *niveum*	200 µL (concentration N.M.) = 100%	Raza et al., 2015
benzothiazole	benzenoid / thiazole / sulfur compound / nitrogen compound	7222	Y	Bacteria	*Mo. fructicola*	1 mol/L = 65%	(Zhou et al., 2019)
benzothiazole	benzenoid / thiazole / sulfur compound / nitrogen compound	7222	Y	Bacteria	*Mo. fructicola*	EC50 = 2.29 x 10^{-2} mol/L	Gao et al., 2018
benzothiazole	benzenoid / thiazole / sulfur compound / nitrogen compound	7222	Y	Bacteria	*Po. chlamydosporia*	mycelium: (≥ 99%) = 158 mg/L	Zou et al., 2007
benzothiazole	benzenoid / thiazole / sulfur compound / nitrogen compound	7222	Y	Bacteria	*Pa. lilacinus*	mycelium: (≥ 99%) = 29 mg/L	Zou et al., 2007
benzothiazole	benzenoid / thiazole / sulfur compound / nitrogen compound	7222	Y	Bacteria	*Per. litchii*	EC50 = 146.4 mg/L	Zheng et al., 2019a
benzothiazole	benzenoid / thiazole / sulfur compound / nitrogen compound	7222	Y	Bacteria	*Pes. photiniae*	7.5 mg = mycelia reduced in 57.7 mm (±82%)	Zhao et al., 2011
benzothiazole	benzenoid / thiazole / sulfur compound / nitrogen compound	7222	Y	Bacteria	*Phyt. capsici*	7.5 mg = mycelia reduced in 60.2 mm (±90%)	Zhao et al., 2011
benzothiazole	benzenoid / thiazole / sulfur compound / nitrogen compound	7222	Y	Bacteria	*Ph. melonis*	7.5 mg = mycelia reduced in 59 mm (±89%)	Zhao et al., 2011
benzothiazole	benzenoid / thiazole / sulfur compound / nitrogen compound	7222	Y	Bacteria	*Ra. solanacearum*	40 µg/100µL (methanol) = ±50%	Raza et al., 2016a
benzothiazole	benzenoid / thiazole / sulfur compound / nitrogen compound	7222	Y	Bacteria	*Rh. solani*	7.5 mg = mycelia reduced in 68.4 mm (±91%)	Zhao et al., 2011
benzothiazole	benzenoid / thiazole / sulfur compound / nitrogen compound	7222	Y	Bacteria	*S. sclerotiorum*	100 and 150 µL (concentration N.M.) = 100%	Fernando et al., 2005
benzyl alcohol	benzenoid / alcohol	244	Y	Fungi	*Bo. cinerea*	625 µL/L = 100%	Guo et al., 2019

Compounds	Chemical classes	PubChem ID	mVOC*	Identified in	phytopathogen inhibited	Inhibitory concentrations	References
benzyl alcohol	benzenoid / alcohol	244	Y	Bacteria	*F. oxysporum* f. sp. *niveum*	200 µL (concentration N.M.) = 70%	Raza et al., 2015
benzyl alcohol	benzenoid / alcohol	244	Y	Bacteria	*T. ethacetica*	50 mM = 100%	Freitas et al., 2022
caryophyllene	terpene	5281515	Y	Bacteria	*Per. litchii*	1000 µL/L (volume N.M.) = mycelia reduced in 32.6 mm (±64%)	Xing et al., 2018
chloroacetic acid, tetradecyl ester	ester / halogenated compound	519540	N	Bacteria	*Co. gloeosporioides*	200 µL (200 µL/mL liquid or 100 mg/mL solid compounds) = 100%	Rajaofera et al., 2019
citronellol	terpene / alcohol	8842	Y	Bacteria	*Al. brassicae*	7.5 mg = mycelia reduced in 26.3 mm (±38%)	Zhao et al., 2011
citronellol	terpene / alcohol	8842	Y	Bacteria	*Bo. cinerea*	7.5 mg = mycelia reduced in 24.4 mm (±34%)	Zhao et al., 2011
citronellol	terpene / alcohol	8842	Y	Bacteria	*Co. capsici*	7.5 mg = mycelia reduced in 44.3 mm (±67%)	Zhao et al., 2011
citronellol	terpene / alcohol	8842	Y	Bacteria	*F. oxysporum*	7.5 mg = mycelia reduced in 53.5 mm (±80%)	Zhao et al., 2011
citronellol	terpene / alcohol	8842	Y	Bacteria	*Phyt. capsici*	7.5 mg = mycelia reduced in 46.7 mm (±69%)	Zhao et al., 2011
citronellol	terpene / alcohol	8842	Y	Bacteria	*Ph. melonis*	7.5 mg = mycelia reduced in 51.7 mm (±78%)	Zhao et al., 2011
citronellol	terpene / alcohol	8842	Y	Bacteria	*Pes. photiniae*	7.5 mg = mycelia reduced in 31.6 mm (±45%)	Zhao et al., 2011
citronellol	terpene / alcohol	8842	Y	Bacteria	*Rh. solani*	7.5 mg = mycelia reduced in 45.7 mm (±61%)	Zhao et al., 2011

Compounds	Chemical classes	PubChem ID	mVOC*	Identified in	phytopathogen inhibited	Inhibitory concentrations	References
cyclohexanol	alcohol	7966	Y	Bacteria	*S. sclerotiorum*	100 and 150 µL (concentration N.M.) = 100%	Fernando et al., 2005
cyclohexene	alkene	8079	N	Bacteria	*Burs. xylophilus*	0.99 mmol = 100%	Gu et al., 2007
decanal	aldehyde	8175	Y	Bacteria	*Burs. xylophilus*	0.20 mmol = 100%	Gu et al., 2007
decanal	aldehyde	8175	Y	Bacteria	*F. oxysporum* f. sp. *niveum*	200 µL (concentration N.M.) = 100%	Raza et al., 2015
decanal	aldehyde	8175	Y	Bacteria	*S. sclerotiorum*	100 and 150 µL (concentration N.M.) = 100%	Fernando et al., 2005
decanoic acid	carboxylic acid	2969	Y	Fungi	*Bo. cinerea*	100 mg/L = 23%	Guo et al., 2019
dimethyl disulfide	sulfide / thioether / sulfur compound	12232	Y	Bacteria	*Ag. tumefasciens*	Effective doses of 20 µL at 50, 100 and 200 µmol (inhibition % N.M.)	Dandurishvili et al., 2011
dimethyl disulfide	sulfide / thioether / sulfur compound	12232	Y	Bacteria	*Ag. tumefasciens*	20 mg = inhibition % N.M.	Chernin et al., 2013
dimethyl disulfide	sulfide / thioether / sulfur compound	12232	Y	Bacteria	*Ag. tumefasciens*	150 µmol = ~100% biofilm formation inhibited	Plyuta et al., 2016
dimethyl disulfide	sulfide / thioether / sulfur compound	12232	Y	Bacteria	*Agrobacterium vitis*	Effective doses of 20 µL at 50, 100 and 200 µmol (inhibition % N.M.)	Dandurishvili et al., 2011
dimethyl disulfide	sulfide / thioether / sulfur compound	12232	Y	Bacteria	*Bo. cinerea*	1000 µM (volume N.M.) = mycelia reduced in ±13 mm (±37%)	Rojas-Solís et al., 2018
dimethyl disulfide	sulfide / thioether / sulfur compound	12232	Y	Bacteria	*Burs. xylophilus*	0.44 mmol = 100%	Gu et al., 2007
dimethyl disulfide	sulfide / thioether / sulfur compound	12232	Y	Bacteria	*F. solani*	200 µL (standard) = 100%	Guevara-Avendaño et al., 2019

Compounds	Chemical classes	PubChem ID	mVOC*	Identified in	phytopathogen inhibited	Inhibitory concentrations	References
dimethyl disulfide	sulfide / thioether / sulfur compound	12232	Y	Bacteria	*Py. aphanidermatum*	1.5 µL (98 g/mL) = 100%	Huang et al., 2018
dimethyl disulfide	sulfide / thioether / sulfur compound	12232	Y	Bacteria	*Py. ultimum*	1 µl (± 11.1 µM) = 30.0mm treated / control 45.0 mm	Ossowicki et al., 2017
dimethyl disulfide	sulfide / thioether / sulfur compound	12232	Y	Bacteria	*Ra. solanacearum*	40 µg/100µL (methanol) = ±50%	Raza et al., 2016a
dimethyl disulfide	sulfide / thioether / sulfur compound	12232	Y	Bacteria	*Rh. solani*	8 µL (98 g/mL) = 100%	Huang et al., 2018
dimethyl disulfide	sulfide / thioether / sulfur compound	12232	Y	Bacteria	*Rh. solani*	1 µl (± 11.1 µM) = 41.0mm treated / control 45.0 mm	Ossowicki et al., 2017
dimethyl disulfide	sulfide / thioether / sulfur compound	12232	Y	Bacteria	*S. sclerotiorum*	100 and 150 µL (concentration N.M.) = 100%	Fernando et al., 2005
dimethyl disulfide	sulfide / thioether / sulfur compound	12232	Y	Bacteria	*S. sclerotiorum*	mycelium MIQ = 31.38 mg<br>sclerotia MIQ = 73.22 mg	Giorgio et al., 2015
dimethyl disulfide	sulfide / thioether / sulfur compound	12232	Y	Bacteria	*Synechococcus* sp.	100 µmol = 100%	Popova et al., 2014
dimethyl disulfide	sulfide / thioether / sulfur compound	12232	Y	Bacteria	*T. ethacetica*	5 mM = 100%	Freitas et al., 2022
dimethyl trisulfide	sulfide / thioether/ sulfur compound	19310	Y	Bacteria	*At. rolfsii*	Complete inhibition at all tested concentrations MIC = 2.65 µg/cm^3	Agisha et al., 2019
dimethyl trisulfide	sulfide / thioether/ sulfur compound	19310	Y	Bacteria	*Co. gloeosporioides*	Complete inhibition at all tested concentrations MIC = 2.65 µg/cm^3	Agisha et al., 2019
dimethyl trisulfide	sulfide / thioether/ sulfur compound	19310	Y	Bacteria	*F. solani*	200 µL (standard) = 100%	Guevara-Avendaño et al., 2019
dimethyl trisulfide	sulfide / thioether/ sulfur compound	19310	Y	Bacteria	*G. moliniformis*	Complete inhibition at all tested concentrations MIC = 2.65 µg/cm^3	Agisha et al., 2019
dimethyl trisulfide	sulfide / thioether/ sulfur compound	19310	Y	Bacteria	*Ma. oryzae*	Complete inhibition at all tested concentrations MIC = 2.65 µg/cm^3	Agisha et al., 2019

Compounds	Chemical classes	PubChem ID	mVOC*	Identified in	phytopathogen inhibited	Inhibitory concentrations	References
dimethyl trisulfide	sulfide / thioether/ sulfur compound	19310	Y	Bacteria	*Phyt. capsici*	Complete inhibition at all tested concentrations MIC = 2.65 µg/cm^3	Agisha et al., 2019
dimethyl trisulfide	sulfide / thioether/ sulfur compound	19310	Y	Bacteria	*Py. myriotilum*	Complete inhibition at all tested concentrations MIC = 2.65 µg/cm^3	Agisha et al., 2019
dimethyl trisulfide	sulfide / thioether/ sulfur compound	19310	Y	Bacteria	*Py. ultimum*	1 µl (± 9.5 µM) = 100%	Ossowicki et al., 2017
dimethyl trisulfide	sulfide / thioether/ sulfur compound	19310	Y	Bacteria	*Ra. pseudosolanacearum*	Complete inhibition at all tested concentrations 21 µg/cm^3 = 100%	Agisha et al., 2019
dimethyl trisulfide	sulfide / thioether/ sulfur compound	19310	Y	Bacteria	*Rh. solani*	Complete inhibition at all tested concentrations MIC = 2.65 µg/cm^3	Agisha et al., 2019
dimethyl trisulfide	sulfide / thioether/ sulfur compound	19310	Y	Bacteria	*Rh. solani*	1 µl (± 9.5 µM) = 100%	Ossowicki et al., 2017
dimethyl trisulfide	sulfide / thioether/ sulfur compound	19310	Y	Bacteria	*S. sclerotiorum*	mycelium MIQ = 24.04 mg sclerotia MIQ = 24.04 mg	Giorgio et al., 2015
dimethyl trisulfide	sulfide / thioether/ sulfur compound	19310	Y	Bacteria	*T. ethacetica*	0.5 mM = 100%	Freitas et al., 2022
dl-limonene	Terpene / monoterpene	22311	Y	Bacteria	*S. sclerotiorum*	mycelium MIQ = 17.2 mg sclerotia MIQ = 30.1 mg	Giorgio et al., 2015
docosane	alkane	12405	Y	Bacteria	*Co. gloeosporioides*	200 µL (200 µL/mL liquid or 100 mg/mL solid compounds) = 79%	Rajaofera et al., 2019
dodecanal	aldehyde	8194	Y	Bacteria	*F. oxysporum* f. sp. *niveum*	200 µL (concentration N.M.) = 100%	Raza et al., 2015
dodecane	alkane	8182	Y	Bacteria	*Burs. xylophilus*	2.0 mmol = 42%	Gu et al., 2007
ethyl 3-hydroxybutyrate	ester / alcohol	62572	N	Fungi	*Bo. cinerea*	1250 µL/L = 58%	Guo et al., 2019
ethyl acetate	ester	8857	Y	Fungi	*Bo. cinerea*	1250 µL/L = 100%	Guo et al., 2019

Compounds	Chemical classes	PubChem ID	mVOC*	Identified in	phytopathogen inhibited	Inhibitory concentrations	References
ethyl acetate	ester	8857	Y	Fungi	*Phyl. citricarpa*	50 µL (concentration N.M.) = 35%	Toffano et al., 2017
ethyl caprate	ester	8048	Y	Fungi	*Bo. cinerea*	1250 µL/L = 34%	Guo et al., 2019
ethyl caprylate	ester	7799	Y	Fungi	*Bo. cinerea*	125 µL/L = 100%	Guo et al., 2019
ethyl cinnamate	alkyl cinnamate / ester	637758	N	Fungi	*Bo. cinerea*	1250 µL/L = 71%	Guo et al., 2019
ethyl octanoate	ester	7799	Y	Fungi	*Phyl. citricarpa*	50 µL (concentration N.M.) = 56%	Toffano et al., 2017
ethyl phenylacetate	benzenoid / ester	7590	N	Bacteria	*Per. litchii*	500 µL/L (volume N.M.) = 100%	Xing et al., 2018
ethyl propionate	ester	7749	Y	Fungi	*Bo. cinerea*	625 µL/L = 100%	Guo et al., 2019
ethyl-2-methylbutyrate	ester	24020	Y	Fungi	*Bo. cinerea*	625 µL/L = 100%	Guo et al., 2019
ethyl-3-hydroxyhexanoate	ester / alcohol	61293	N	Fungi	*Bo. cinerea*	1250 µL/L = 95%	Guo et al., 2019
ethylbenzene	benzenoid / alkylbenzene	7500	Y	Bacteria	*F. oxysporum* f. sp. *niveum*	200 µL (concentration N.M.) = 62%	Raza et al., 2015
ethylbenzene	benzenoid / alkylbenzene	7500	Y	Bacteria	*Ra. solanacearum*	40 µg/100µL (methanol) = ±50%	Raza et al., 2016a
heptadecane	alkane	12398	Y	Bacteria	*F. oxysporum* f. sp. *niveum*	200 µL (concentration N.M.) = 15%	Raza et al., 2015
hexadecanal	aldehyde	984	N	Bacteria	*F. oxysporum* f. sp. *niveum*	200 µL (concentration N.M.) = 100%	Raza et al., 2015
hexadecane	alkane	11006	Y	Bacteria	*Burs. xylophilus*	2.0 mmol = 22%	Gu et al., 2007
hexadecane	alkane	11006	Y	Bacteria	*F. oxysporum* f. sp. *niveum*	200 µL (concentration N.M.) = 20%	Raza et al., 2015
hexanedioic acid, bis(2-ethylhexyl) ester	diester	7641	N	Bacteria	*Co. gloeosporioides*	200 µL (200 µL/mL liquid or 100 mg/mL solid compounds) = 11%	Rajaofera et al., 2019
hexanoic acid	acid / carboxylic acid	8892	Y	Fungi	*Bo. cinerea*	625 µL/L = 100%	Guo et al., 2019

Compounds	Chemical classes	PubChem ID	mVOC*	Identified in	phytopathogen inhibited	Inhibitory concentrations	References
hexyl alcohol	alcohol	8103	Y	Fungi	*Bo. cinerea*	625 µL/L = 100%	Guo et al., 2019
indole	indole / nitrogen compound	798	Y	Bacteria	*F. oxysporum* f. sp. *niveum*	200 µL (concentration N.M.) = 60%	Raza et al., 2015
isoamyl acetate	ester	31276	Y	Fungi	*Bo. cinerea*	625 µL/L = 100%	Guo et al., 2019
isoamyl propionate	ester	7772	Y	Bacteria	*Phyt. capsici*	10 µg/mL (volume N.M.) = 35% inhibition	Syed-Ab-Rahman et al., 2019
isobutyl acetate	ester	8038	Y	Fungi	*Bo. cinerea*	625 µL/L = 100%	Guo et al., 2019
isovaleric acid	carboxylic acid	10430	N	Bacteria	*Co. lindemuthianum*	10 µL (102.3 g/mol) = up to 94%	Martins et al., 2019
isovaleric acid	carboxylic acid	10430	N	Bacteria	*Phyt. capsici*	10 µg/mL (volume N.M.) = ± 40% inhibition	Syed-Ab-Rahman et al., 2019
lauric acid	carboxylic acid	3893	Y	Fungi	*Bo. cinerea*	100 mg/L = 11%	Guo et al., 2019
m-cymene	benzenoid / terpene	10812	Y	Bacteria	*S. sclerotiorum*	mycelium MIQ = 13.77 mg<br>sclerotia MIQ = 17.22 mg	Giorgio et al., 2015
methyl anthranilate	benzenoid / amine / ester / nitrogen compound	8635	Y	Bacteria	*Per. litchii*	1000 µL/L (volume N.M.) = mycelia reduced in 38.7 mm (±76%)	Xing et al., 2018
methyl jasmonate	jasmonate derivate / ester	5281929	N	Bacteria	*Per. litchii*	EC50 = 206.96 mg/L	Zheng et al., 2019a
methyl salicylate	benzenoid / ester / alcohol	4133	Y	Bacteria	*Per. litchii*	200 µL/L (volume N.M.) = 100%	Xing et al., 2018
Methyl vinyl ketone	ketone	6570	N	Bacteria	*Cladosporium cladosporioides*	0,22 mg/L = 99%	Herrington et al., 1987
methyl-1-butanol	alcohol	22386	Y	Fungi	*Phyl. citricarpa*	50 µL (concentration N.M.) = 100%	Toffano et al., 2017
methyl-2-methylpentanoate	ester	519890	Y	Bacteria	*Rh. solani*	1M = 47% (1 day) and 25% (2 days)	Cordovez et al., 2015

Compounds	Chemical classes	PubChem ID	mVOC*	Identified in	phytopathogen inhibited	Inhibitory concentrations	References
methylamine	amine / nitrogen compound	6329	Y	Bacteria	*Burkholderia arboris*	MVIC = 20 mM	Sannino et al., 2017
methylamine	amine / nitrogen compound	6329	Y	Bacteria	*Burkholderia cenocepacia*	MVIC = 10 mM	Sannino et al., 2017
methylamine	amine / nitrogen compound	6329	Y	Bacteria	*Burkholderia contaminans*	MVIC = 20 mM	Sannino et al., 2017
methylamine	amine / nitrogen compound	6329	Y	Bacteria	*Burkholderia latens*	MVIC > 35 mM	Sannino et al., 2017
methylamine	amine / nitrogen compound	6329	Y	Bacteria	*Burkholderia metallica*	MVIC = 20 mM	Sannino et al., 2017
methylamine	amine / nitrogen compound	6329	Y	Bacteria	*Burkholderia multivorans*	MVIC = 20 mM	Sannino et al., 2017
methylamine	amine / nitrogen compound	6329	Y	Bacteria	*Burkholderia pseudomultivorans*	MVIC = 20 mM	Sannino et al., 2017
methylamine	amine / nitrogen compound	6329	Y	Bacteria	*Burkholderia stabilis*	MVIC = 20 mM	Sannino et al., 2017
methylamine	amine / nitrogen compound	6329	Y	Bacteria	*Burkholderia ubonensis*	MVIC = 20 mM	Sannino et al., 2017
methylamine	amine / nitrogen compound	6329	Y	Bacteria	*Po. chlamydosporia*	spore: 9 mg/L ≥ 99% mycelium: 33 mg/L ≥ 99%	Zou et al., 2007
methylamine	amine / nitrogen compound	6329	Y	Bacteria	*Pa. lilacinus*	spore: 7 mg/L ≥ 99% mycelium: 21 mg/L ≥ 99%	Zou et al., 2007
m-xylene	benzenoid / alkylbenzene	7929	Y	Bacteria	*Ra. solanacearum*	40 µg/100µL (methanol) = ±50%	Raza et al., 2016a
n-hexadecanoic acid	carboxylic acid	985	Y	Bacteria	*F. oxysporum* f. sp. *niveum*	200 µL (concentration N.M.) = 35%	Raza et al., 2015
nonadecane	alkane	12401	Y	Bacteria	*F. oxysporum* f. sp. *niveum*	200 µL (concentration N.M.) = 10%	Raza et al., 2015
nonadecanone	ketone	137263	N	Bacteria	*F. oxysporum* f. sp. *niveum*	200 µL (concentration N.M.) = 4%	Raza et al., 2015
nonanal	aldehyde	31289	Y	Bacteria	*Cl. michiganensis* subsp. *sepedonicus*	100 µl (10 and 100 µL/mL and undiluted) = cell viability reduced in up to ±5 log CFU/mL	Rajer et al., 2017

Compounds	Chemical classes	PubChem ID	mVOC*	Identified in	phytopathogen inhibited	Inhibitory concentrations	References
nonanal	aldehyde	31289	Y	Bacteria	*F. oxysporum* f. sp. *niveum*	200 µL (concentration N.M.) = 100%	Raza et al., 2015
nonanal	aldehyde	31289	Y	Bacteria	*S. sclerotiorum*	100 and 150 µL (concentration N.M.) = 100%	Fernando et al., 2005
nonane	alkane	8141	Y	Bacteria	*Burs. xylophilus*	2.0 mmol = 79%	Gu et al., 2007
nonanoic acid	carboxylic acid	8158	Y	Bacteria	*F. oxysporum* f. sp. *niveum*	200 µL (concentration N.M.) = 35%	Raza et al., 2015
ocimene	terpene	5281553	Y	Fungi	*F. oxysporum*	1000 µg/mL = ± 20%	Medina-Romero et al., 2017
octadecane	alkane	11635	Y	Bacteria	*Co. gloeosporioides*	200 µL (200 µL/mL liquid or 100 mg/mL solid compounds) = 29%	Rajaofera et al., 2019
oleic acid	carboxylic acid / alkene	445639	Y	Bacteria	*F. oxysporum* f. sp. *niveum*	200 µL (concentration N.M.) = 20%	Raza et al., 2015
pentadecane	alkane	12391	Y	Bacteria	*F. oxysporum* f. sp. *niveum*	200 µL (concentration N.M.) = 20%	Raza et al., 2015
phenol	benzenoid / alcohol	996	Y	Bacteria	*Burs. xylophilus*	1.71 mmol = 100%	Gu et al., 2007
phenol	benzenoid / alcohol	996	Y	Bacteria	*F. oxysporum* f. sp. *niveum*	200 µL (concentration N.M.) = 100%	Raza et al., 2015
phenylacetaldehyde (benzacetaldehyde)	benzenoid / aldehyde	998	Y	Bacteria	*Burs. xylophilus*	0.85 mmol = 100%	Gu et al., 2007
phenylacetaldehyde	benzenoid / aldehyde	998	Y	Bacteria	*Po. chlamydosporia*	spore: 8 mg/L ≥ 99% mycelium: 34 mg/L ≥ 99%	Zou et al., 2007
phenylacetaldehyde	benzenoid / aldehyde	998	Y	Bacteria	*Pa. lilacinus*	spore: 6 mg/L ≥ 99% mycelium: 17 mg/L ≥ 99%	Zou et al., 2007
phenylacetaldehyde	benzenoid / aldehyde	998	Y	Bacteria	*T. ethacetica*	5 mM = 100%	Freitas et al., 2022
propanoic acid	carboxylic acid	1032	Y	Bacteria	*Burs. xylophilus*	2.0 mmol = 25%	Gu et al., 2007

Compounds	Chemical classes	PubChem ID	mVOC*	Identified in	phytopathogen inhibited	Inhibitory concentrations	References
propanone	ketone	180	Y	Bacteria	*Burs. xylophilus*	2.0 mmol = 75%	Gu et al., 2007
salicylic acid	beta hydroxy acid / benzenoid	338	N	Bacteria	*Per. litchii*	EC50 = 175.97 mg/L	Zheng et al., 2019a
styrene	alkene / benzenoid	7501	Y	Bacteria	*F. oxysporum* f. sp. *cucumerinum*	80 µL (standard) = 15%	Wu et al., 2015
styrene	alkene / benzenoid	7501	Y	Bacteria	*S. sclerotiorum*	80 µL (standard) = 51%	Wu et al., 2015
terpineol	alcohol / terpene	17100	Y	Bacteria	*Burs. xylophilus*	2.0 mmol = 78%	Gu et al., 2007
terpinolene	terpene	11463	Y	Fungi	*F. oxysporum*	1000 µg/mL = ± 20%	Medina-Romero et al., 2017
tetradecane	alkane	12389	Y	Bacteria	*Burs. xylophilus*	2.0 mmol = 19%	Gu et al., 2007
tetradecane	alkane	12389	Y	Bacteria	*F. oxysporum* f. sp. *cucumerinum*	80 µL (standard) = 21%	Wu et al., 2015
tetradecane	alkane	12389	Y	Bacteria	*F. oxysporum* f. sp. *niveum*	200 µL (concentration N.M.) = 30%	Raza et al., 2015
tetradecane	alkane	12389	Y	Bacteria	*S. sclerotiorum*	80 µL (standard) = 13%	Wu et al., 2015
tetradecanoic acid	carboxylic acid	11005	Y	Bacteria	*F. oxysporum* f. sp. *niveum*	200 µL (concentration N.M.) = 20%	Raza et al., 2015
tetradecanol	alcohol	8209	Y	Bacteria	*F. oxysporum* f. sp. *niveum*	200 µL (concentration N.M.) = 50%	Raza et al., 2015
thiophene	thiophene / thioether / sulfur compound	8030	Y	Bacteria	*Bo. cinerea*	1.35 µL/mL = 83% EC50 = 6.67 µL/mL	Gotor-Vila et al., 2017
thiophene	thiophene / thioether / sulfur compound	8030	Y	Bacteria	*Mo. fructicola*	1.35 µL/mL = 100% EC50 = 0.06 µL/mL	Gotor-Vila et al., 2017
thiophene	thiophene / thioether / sulfur compound	8030	Y	Bacteria	*Mo. laxa*	1.35 µL/mL = 95% EC50 = 2.62 µL/mL	Gotor-Vila et al., 2017
toluene	benzenoid / alkylbenzene	1140	Y	Bacteria	*F. oxysporum* f. sp. *cucumerinum*	80 µL (standard) = 9%	Wu et al., 2015
toluene	benzenoid / alkylbenzene	1140	Y	Bacteria	*F. oxysporum* f. sp. *niveum*	200 µL (concentration N.M.) = 100%	Raza et al., 2015
toluene	benzenoid / alkylbenzene	1140	Y	Bacteria	*Ra. solanacearum*	40 µg/100µL (methanol) = ±50%	Raza et al., 2016a

Compounds	Chemical classes	PubChem ID	mVOC*	Identified in	phytopathogen inhibited	Inhibitory concentrations	References
toluene	benzenoid / alkylbenzene	1140	Y	Bacteria	*S. sclerotiorum*	80 µL (standard) = 51%	Wu et al., 2015
trans-cinnamaldehyde	aldehyde / carboxylic acid / benzenoid	637511	Y	Fungi	*Bo. cinerea*	6.25 µL/L = 100%	Guo et al., 2019
trimethyl pyrazine	pyrazine / nitrogen compound / heterocyclic compound	26808	Y	Bacteria	*Burs. xylophilus*	2.0 mmol = 13%	Gu et al., 2007
undecanal	aldehyde	8186	Y	Bacteria	*F. oxysporum* f. sp. *niveum*	200 µL (concentration N.M.) = 100%	Raza et al., 2015
undecanoic acid	carboxylic acid	8180	N	Bacteria	*F. oxysporum* f. sp. *niveum*	200 µL (concentration N.M.) = 5%	Raza et al., 2015
xylene	benzenoid	7237	**	Bacteria	*F. oxysporum* f. sp. *niveum*	200 µL (concentration N.M.) = 85%	Raza et al., 2015
VOC mixture: 1,3,5-trichloro-2-methoxy benzene (A); methyl butanoate (B); 3-octanone (C); methyl 2-methylpentanoate (D); methyl 3-methylpentanoate (E)	benzenoid (A); ester (B); ketone (C); ester (D); ester (E)	6884 (A); 12180 (B); 246728 (C); 519890 (D); 519891 (E)	Y (A); ** (B); Y (C); Y (D); **(E)	Bacteria	*Rh. solani*	200mM (final concentration) = 58% (1 day) and 42% (2 days)	Cordovez et al., 2015
VOC mixture: acetoin (A); 2-methyl butyric acid (B)	ketone (A); acid (B)	179 (A); 8314 (B)	Y (A); Y (B)	Bacteria	*Co. lindemuthianum*	10 µL of each compound (acetoin 88.8 g/mol, 2-methyl butyric acid 102.3 g/mol) = up to 94%	Martins et al., 2019
VOC mixture: acetoin (A); isovaleric acid (B)	ketone (A); acid (B)	179 (A); 10340 (B)	Y (A); N (B)	Bacteria	*Co. lindemuthianum*	10 µL of each compound (acetoin 88.8 g/mol, isovaleric acid 102.3 g/mol) = up to 94%	Martins et al., 2019
VOC mixture: isovaleric acid (A); 2-methyl butyric acid (B)	acid (A); acid (B)	10430 (A); 8314 (B)	N (A); Y (B)	Bacteria	*Co. lindemuthianum*	10 µL of each compound (2-methyl butyric acid 102.3 g/mol, isovaleric acid 102.3 g/mol) = up to 94% inhibition	Martins et al., 2019

Compounds	Chemical classes	PubChem ID	mVOC*	Identified in	phytopathogen inhibited	Inhibitory concentrations	References
VOC mixture: benzaldehyde (A); 2-ethylhexan-1-ol (B); 2-decanone (C)	benzenoid (A); alcohol (B); ketone (C)	240 (A); 7720 (B); 12741 (C)	Y (A); Y (B); Y (C)	Bacteria	*Co. acutatum*	20 µL (proportion of each compound relative to its GC peak area GC – 7:24:1) = 99%	Che et al., 2017
VOC mixture: 2-phenylethanol (A); 2-methyl-1-butanol (B); 3-methyl-1-butanol (C)	alcohol (A); alcohol (B); alcohol (C)	6054 (A); 8723 (B); 31260 (C)	Y (A); Y (B); Y (C)	Fungi	*F. oxysporum*	1000 µg/mL = 100%	Medina-Romero et al., 2017
VOC mixture: ethanol (A); ethyl acetate (B); 3-methyl-1-butanol (C); 2-methyl-1-butanol (D); 2-phenylethanol (E); ethyl octanoate (F)	alcohol (A); esther (B); alcohol (C); alcohol (D); alcohol (E); Esther (F)	702 (A); 8857 (B); 31260 (C); 8723 (D); 6054 (E); 7799 (F)	Y (A); Y (B); Y (C); Y (D); Y(E); Y (F)	Bacteria	*Phyl. citricarpa*	0.4 – 2.0 µ/mL at 4 – 12 days = up to 100% (0.8 – 2.0 µ/mL at 4 days)	Toffano et al., 2017
VOC mixture: DMDS (A); S-methyl thioacetate (MTA) (B)	sulfide/thioether (A); thioesther (B)	12232 (A); 73750 (B)	Y (A); ** (B)	Bacteria	*Py. ultimum*	1 µl of each compound (MTA 11.4 µM, DMDS± 11.1 µM) = ±27.0mm treated / control 45.0 mm	Ossowicki et al., 2017
VOC mixture: DMDS (A); S-methyl thioacetate (MTA) (B)	sulfide/thioether (A); thioesther (B)	12232 (A); 73750 (B)	Y (A); ** (B)	Bacteria	*Rh. solani*	1 µl of each compound (MTA 11.4 µM, DMDS ± 11.1 µM) = ±29.0mm treated / control 45.0 mm	Ossowicki et al., 2017
VOC mixture: 2-phenylethanol (A); 2-methyl-1-butanol(B); 3-methyl-1-butanol(C); eucalyptol (D); ocimene (E); terpinolene (F)	alcohol/benzenoid(A); alcohol (B); alcohol(C); cyclic ether (D); alkatriene(E); monoterpene (F)	6054 (A); 8723 (B); 31260 (C); 2758 (D); 5281553 (E); 11463 (F)	Y (A); Y (B); Y (C); ** (D); Y (E); Y (F)	Fungi	*F. oxysporum*	0.5 mL (125 and 250 µg/mL of each compound) = 100%	Macías-Rubalcava et al., 2018
VOC mixture: 2-phenylethanol (A); 2-methyl-1-butanol(B); 3-methyl-1-butanol(C); eucalyptol (D); ocimene (E); terpinolene (F)	alcohol/benzenoid(A); alcohol (B); alcohol(C); cyclic ether (D); alkatriene(E); monoterpene (F)	6054 (A); 8723 (B); 31260 (C); 2758 (D); 5281553 (E); 11463 (F)	Y (A); Y (B); Y (C); ** (D); Y (E); Y (F)	Fungi	*F. oxysporum*	1000 µg/mL = 100%	Medina-Romero et al., 2017
VOC mixture: acetaldehyde (A); ethyl acetate (B); 2-	aldehyde (A); ester (B); ketone (C); fatty acid	177 (A); 8857 (B); 6569 (C);	** (A); Y (B);	Fungi	*Py. ultimum*, *Bipolaris sorokiniana*,	15uL (proportion according with the GC-	Mitchell et al., 2010

Compounds	Chemical classes	PubChem ID	mVOC*	Identified in	phytopathogen inhibited	Inhibitory concentrations	References
butanone (C); propanoic acid, 2-methyl-, methyl ester (D); ethanol (E); acetic acid, 2-methylpropyl ester (F); 1-propanol, 2-methyl- (G); 2-butenal, 2-methyl-, (*E*)- (H); 1-butanol, 3-methyl-, acetate (I); propanoic acid, 2-methyl-, 3-methylbutyl ester (J); 1-butanol, 3-methyl- (K); propanoic acid, 2-methyl- (L); acetic acid, 2-phenylethyl ester (M)	methyl ester (D); alcohol (E); acetate ester (F); alcohol (G); aldehyde (H); acetate ester (I); fatty acid ester (J); alcohol (K); branched fatty acid (L); acetate ester (M)	11039 (D); 702 (E); 8038 (F); 6560 (G); 5321950 (H); 31276 (I); 519786 (J); 31260 (K); 6590 (L); 7654 (M)	** (C); ** (D); Y (E); Y (F); Y (G); ** (H); Y (I); ** (J); Y (K); ** (L); ** (M)		*Stagonospora* sp., *F. oxysporum; F. solani; Verticillium dahliae; Bo. cinerea; Aspergillus fumigatus* and many others (view reference for entire list)	MS peaks) = 100% for almost organisms (view reference for checking each inhibition rate)	

Notes: The inhibitory activity of each compound is expressed as it was reported by the respective authors. %: percentage of growth inhibition; MIQ: minimum inhibitory quantity; MIC: minimum inhibitory concentration; MVIC: minimum volatile inhibitory concentration; EC50 and EC95: effective concentrations causing 50 and 95% of inhibition, respectively; D.I: disease incidence; and N.M.: information is not mentioned. Only the most effective concentration tested is shown in the table, please consult the reference for other values or information. As we standardized the name of all compounds by the PUBCHEM ID, the compounds may appear with a different name than the reported in the original article.

*mVOC column refers if the compound is (Y, yes) or is not (N, no) listed in the mVOC 2.0 Database (Lemfack et al., 2018).

** mVOC database website was unavailable at the moment of the search for those compounds until the conclusion of this final draft.

REFERENCES

Agisha, V. N., Kumar, A., Eapen, S. J., Sheoran, N., and Suseelabhai, R. (2019). Broad-spectrum antimicrobial activity of volatile organic compounds from endophytic *Pseudomonas putida* BP25 against diverse plant pathogens. *Biocontrol Sci. Technol.* 29, 1069–1089. doi:10.1080/09583157.2019.1657067.

Boukaew, S., and Prasertsan, P. (2018). Inhibitory effects of acetophenone or phenylethyl alcohol as fumigant to protect soybean seeds against two aflatoxin-producing fungi. *J. Food Sci. Technol.* 55, 5123–5132. doi:10.1007/s13197-018-3458-6.

Che, J., Liu, B., Liu, G., Chen, Q., and Lan, J. (2017). Volatile organic compounds produced by *Lysinibacillus* sp. FJAT-4748 possess antifungal activity against *Colletotrichum acutatum*. *Biocontrol Sci. Technol.* 27, 1349–1362. doi:10.1080/09583157.2017.1397600.

Cordovez, V., Carrion, V. J., Etalo, D. W., Mumm, R., Zhu, H., van Wezel, G. P., et al. (2015). Diversity and functions of volatile organic compounds produced by Streptomyces from a disease-suppressive soil. *Front. Microbiol.* 6, 1–13. doi:10.3389/fmicb.2015.01081.

Dandurishvili, N., Toklikishvili, N., Ovadis, M., Eliashvili, P., Giorgobiani, N., Keshelava, R., et al. (2011). Broad-range antagonistic rhizobacteria *Pseudomonas fluorescens* and *Serratia plymuthica* suppress *Agrobacterium* crown gall tumours on tomato plants. *J. Appl. Microbiol.* 110, 341–352. doi:10.1111/j.1365-2672.2010.04891.x.

Di Francesco, A., Di Foggia, M., and Baraldi, E. (2020). *Aureobasidium pullulans* volatile organic compounds as alternative postharvest method to control brown rot of stone fruits. *Food Microbiol.* 87, 103395. doi:10.1016/J.FM.2019.103395.

Di Francesco, A., Milella, F., Mari, M., and Roberti, R. (2017). A preliminary investigation into *Aureobasidium pullulans* as a potential biocontrol agent against *Phytophthora infestans* of tomato. *Biol. Control* 114, 144–149. doi:10.1016/J.BIOCONTROL.2017.08.010.

Di Francesco, A., Ugolini, L., Lazzeri, L., and Mari, M. (2015). Production of volatile organic compounds by *Aureobasidium pullulans* as a potential mechanism of action against postharvest fruit pathogens. *Biol. Control* 81, 8–14. doi:10.1016/j.biocontrol.2014.10.004.

Dickschat, J. S., Martens, T., Brinkhoff, T., Simon, M., and Schulz, S. (2005). Volatiles released by a *Streptomyces* species isolated from the North Sea. *Chem. Biodivers.* 2, 837–865. doi:10.1002/cbdv.200590062.

Fernando, W. G. D., Ramarathnam, R., Krishnamoorthy, A. S., and Savchuk, S. C. (2005). Identification and use of potential bacterial organic antifungal volatiles in biocontrol. *Soil Biol. Biochem.* 37, 955–964. doi:10.1016/j.soilbio.2004.10.021.

Freitas, C. S. A., Maciel, L. F., Corrêa dos Santos, R. A., Costa, O. M. M. M., Maia, F. C. B., Rabelo, R. S., et al. (2022). Bacterial volatile organic compounds induce adverse ultrastructural changes and DNA damage to the sugarcane pathogenic fungus *Thielaviopsis ethacetica*. *Environ. Microbiol.* doi:10.1111/1462-2920.15876.

Gao, H., Li, P., Xu, X., Zeng, Q., and Guan, W. (2018). Research on volatile organic compounds from *Bacillus subtilis* CF-3: biocontrol effects on fruit fungal pathogens and dynamic changes during fermentation. *Front. Microbiol.* 9. doi:10.3389/fmicb.2018.00456.

Gao, Z., Zhang, B., Liu, H., Han, J., and Zhang, Y. (2017). Identification of endophytic *Bacillus*

velezensis ZSY-1 strain and antifungal activity of its volatile compounds against *Alternaria solani* and *Botrytis cinerea*. *Biol. Control* 105, 27–39. doi:10.1016/j.biocontrol.2016.11.007.

Giorgio, A., De Stradis, A., Lo Cantore, P., and Iacobellis, N. S. (2015). Biocide effects of volatile organic compounds produced by potential biocontrol rhizobacteria on *Sclerotinia sclerotiorum*. *Front. Microbiol.* 6. doi:10.3389/fmicb.2015.01056.

Gotor-Vila, A., Teixidó, N., Di Francesco, A., Usall, J., Ugolini, L., Torres, R., et al. (2017). Antifungal effect of volatile organic compounds produced by *Bacillus amyloliquefaciens* CPA-8 against fruit pathogen decays of cherry. *Food Microbiol.* 64, 219–225. doi:10.1016/j.fm.2017.01.006.

Gu, Y. Q., Mo, M. H., Zhou, J. P., Zou, C. S., and Zhang, K. Q. (2007). Evaluation and identification of potential organic nematicidal volatiles from soil bacteria. *Soil Biol. Biochem.* 39, 2567–2575. doi:10.1016/j.soilbio.2007.05.011.

Guevara-Avendaño, E., Bejarano-Bolívar, A. A., Kiel-Martínez, A. L., Ramírez-Vázquez, M., Méndez-Bravo, A., von Wobeser, E. A., et al. (2019). Avocado rhizobacteria emit volatile organic compounds with antifungal activity against *Fusarium solani*, *Fusarium* sp. associated with Kuroshio shot hole borer, and *Colletotrichum gloeosporioides*. *Microbiol. Res.* 219, 74–83. doi:10.1016/j.micres.2018.11.009.

Guo, H., Qin, X., Wu, Y., Yu, W., Liu, J., Xi, Y., et al. (2019). Biocontrol of gray mold of cherry tomatoes with the volatile organic monomer from *Hanseniaspora uvarum*, trans-cinnamaldehyde. *Food Bioprocess Technol.* 12, 1809–1820. doi:10.1007/s11947-019-02319-6.

Han, S. H., Lee, S. J., Moon, J. H., Park, K. H., Yang, K. Y., Cho, B. H., et al. (2006). GacS-dependent production of 2R, 3R-butanediol by *Pseudomonas chlororaphis* O6 is a major determinant for eliciting systemic resistance against *Erwinia carotovora* but not against *Pseudomonas syringae* pv. tabaci in tobacco. *Mol. Plant-Microbe Interact.* 19, 924–930. doi:10.1094/MPMI-19-0924.

Herrington, P. R., Craig, J. T., and Sheridan, J. E. (1987). Methyl vinyl ketone: a volatile fungistatic inhibitor from *Streptomyces griseoruber*. *Soil Biol. Biochem.* 19, 509–512. doi:10.1016/0038-0717(87)90092-7.

Howell, C. R. (1988). Production of ammonia by *Enterobacter cloacae* and its possible role in the biological control of *Pythium* preemergence damping-off by the bacterium. *Phytopathology* 78, 1075. doi:10.1094/phyto-78-1075.

Huang, J. S., Peng, Y. H., Chung, K. R., and Huang, J. W. (2018). Suppressive efficacy of volatile compounds produced by *Bacillus mycoides* on damping-off pathogens of cabbage seedlings. *J. Agric. Sci.* 156, 795–809. doi:10.1017/S0021859618000746.

Kishimoto, K., Matsui, K., Ozawa, R., and Takabayashi, J. (2007). Volatile 1-octen-3-ol induces a defensive response in *Arabidopsis thaliana*. *J. Gen. Plant Pathol.* 73, 35–37. doi:10.1007/s10327-006-0314-8.

Koitabashi, M., Kajitani, Y., and Hirashima, K. (2004). Antifungal substances produced by fungal strain Kyu-W63 from wheat leaf and its taxonomic position. *J. Gen. Plant Pathol. 2004 702* 70, 124–130. doi:10.1007/S10327-003-0095-2.

Lemfack, M. C., Gohlke, B. O., Toguem, S. M. T., Preissner, S., Piechulla, B., and Preissner, R. (2018). MVOC 2.0: A database of microbial volatiles. *Nucleic Acids Res.* 46, D1261–D1265. doi:10.1093/nar/gkx1016.

Macías-Rubalcava, M. L., Sánchez-Fernández, R. E., Roque-Flores, G., Lappe-Oliveras, P., and Medina-Romero, Y. M. (2018). Volatile organic compounds from *Hypoxylon anthochroum* endophytic strains as postharvest mycofumigation alternative for cherry tomatoes. *Food Microbiol.* 76, 363–373. doi:10.1016/j.fm.2018.06.014.

Martins, S. J., Faria, A. F., Pedroso, M. P., Cunha, M. G., Rocha, M. R., and Medeiros, F. H. V. (2019). Microbial volatiles organic compounds control anthracnose (*Colletotrichum lindemuthianum*) in common bean (*Phaseolus vulgaris* L.). *Biol. Control* 131, 36–42. doi:10.1016/j.biocontrol.2019.01.003.

Medina-Romero, Y. M., Roque-Flores, G., and Macías-Rubalcava, M. L. (2017). Volatile organic compounds from endophytic fungi as innovative postharvest control of *Fusarium oxysporum* in cherry tomato fruits. *Appl. Microbiol. Biotechnol.* 101, 8209–8222. doi:10.1007/s00253-017-8542-8.

Mitchell, A. M., Strobel, G. A., Moore, E., Robison, R., and Sears, J. (2010). Volatile antimicrobials from *Muscodor crispans,* a novel endophytic fungus. *Microbiology* 156, 270–277. doi:10.1099/mic.0.032540-0.

Munjal, V., Nadakkakath, A. V., Sheoran, N., Kundu, A., Venugopal, V., Subaharan, K., et al. (2016). Genotyping and identification of broad spectrum antimicrobial volatiles in black pepper root endophytic biocontrol agent, *Bacillus megaterium* BP17. *Biol. Control* 92, 66–76. doi:10.1016/j.biocontrol.2015.09.005.

Ossowicki, A., Jafra, S., and Garbeva, P. (2017). The antimicrobial volatile power of the rhizospheric isolate *Pseudomonas donghuensis* P482. *PLoS One* 12, e0174362. doi:10.1371/journal.pone.0174362.

Plyuta, V., Lipasova, V., Popova, A., Koksharova, O., Kuznetsov, A., Szegedi, E., et al. (2016). Influence of volatile organic compounds emitted by Pseudomonas and Serratia strains on Agrobacterium tumefaciens biofilms. *APMIS* 124, 586–594. doi:10.1111/APM.12547.

Popova, A. A., Koksharova, O. A., Lipasova, V. A., Zaitseva, J. V., Katkova-Zhukotskaya, O. A., Eremina, S. I., et al. (2014). Inhibitory and toxic effects of volatiles emitted by strains of *Pseudomonas* and *Serratia* on growth and survival of selected microorganisms, *Caenorhabditis elegans*, and *Drosophila melanogaster*. *Biomed Res. Int.* 2014. doi:10.1155/2014/125704.

Rajaofera, M. J. N., Wang, Y., Dahar, G. Y., Jin, P., Fan, L., Xu, L., et al. (2019). Volatile organic compounds of *Bacillus atrophaeus* HAB-5 inhibit the growth of *Colletotrichum gloeosporioides*. *Pestic. Biochem. Physiol.* 156, 170–176. doi:10.1016/j.pestbp.2019.02.019.

Rajer, F. U., Wu, H., Xie, Y., Xie, S., Raza, W., Tahir, H. A. S., et al. (2017). Volatile organic compounds produced by a soil-isolate, *Bacillus subtilis* FA26 induce adverse ultra-structural changes to the cells of *Clavibacter michiganensis* ssp. *Sepedonicus*, the causal agent of bacterial ring rot of potato. *Microbiol. (United Kingdom)* 163, 523–530. doi:10.1099/mic.0.000451.

Raza, W., Ling, N., Liu, D., Wei, Z., Huang, Q., and Shen, Q. (2016). Volatile organic compounds produced by *Pseudomonas fluorescens* WR-1 restrict the growth and virulence traits of *Ralstonia solanacearum*. *Microbiol. Res.* 192, 103–113. doi:10.1016/j.micres.2016.05.014.

Raza, W., Yuan, J., Ling, N., Huang, Q., and Shen, Q. (2015). Production of volatile organic compounds by an antagonistic strain *Paenibacillus polymyxa* WR-2 in the presence of root exudates and organic fertilizer and their antifungal activity against *Fusarium oxysporum* f. sp. *niveum*. *Biol. Control* 80, 89–95. doi:10.1016/j.biocontrol.2014.09.004.

Rojas-Solís, D., Zetter-Salmón, E., Contreras-Pérez, M., Rocha-Granados, M. del C., Macías-Rodríguez, L., and Santoyo, G. (2018). *Pseudomonas stutzeri* E25 and *Stenotrophomonas maltophilia* CR71 endophytes produce antifungal volatile organic compounds and exhibit additive plant growth-promoting effects. *Biocatal. Agric. Biotechnol.* 13, 46–52. doi:10.1016/j.bcab.2017.11.007.

Ryu, C. M., Farag, M. A., Hu, C. H., Reddy, M. S., Kloepper, J. W., and Paré, P. W. (2004). Bacterial volatiles induce systemic resistance in *Arabidopsis*. *Plant Physiol.* 134, 1017–1026. doi:10.1104/pp.103.026583.

Sannino, F., Parrilli, E., Apuzzo, G. A., de Pascale, D., Tedesco, P., Maida, I., et al. (2017). *Pseudoalteromonas haloplanktis* produces methylamine, a volatile compound active against *Burkholderia cepacia* complex strains. *N. Biotechnol.* 35, 13–18. doi:10.1016/j.nbt.2016.10.009.

Syed-Ab-Rahman, S. F., Carvalhais, L. C., Chua, E. T., Chung, F. Y., Moyle, P. M., Eltanahy, E. G., et al. (2019). Soil bacterial diffusible and volatile organic compounds inhibit *Phytophthora capsici* and promote plant growth. *Sci. Total Environ.* 692, 267–280. doi:10.1016/j.scitotenv.2019.07.061.

Tahir, H. A. S., Gu, Q., Wu, H., Niu, Y., Huo, R., and Gao, X. (2017). *Bacillus* volatiles adversely affect the physiology and ultra-structure of *Ralstonia solanacearum* and induce systemic resistance in tobacco against bacterial wilt. *Sci. Rep.* 7, 1–15. doi:10.1038/srep40481.

Toffano, L., Fialho, M. B., and Pascholati, S. F. (2017). Potential of fumigation of orange fruits with volatile organic compounds produced by *Saccharomyces cerevisiae* to control citrus black spot disease at postharvest. *Biol. Control* 108, 77–82. doi:10.1016/j.biocontrol.2017.02.009.

Vlassi, A., Nesler, A., Perazzolli, M., Lazazzara, V., Büschl, C., Parich, A., et al. (2020). Volatile organic compounds from *Lysobacter capsici* AZ78 as potential candidates for biological control of soilborne plant pathogens. *Front. Microbiol.* 0, 1748. doi:10.3389/FMICB.2020.01748.

Wolfgang, A., Taffner, J., Guimarães, R. A., Coyne, D., and Berg, G. (2019). Novel strategies for soil-borne diseases: Exploiting the microbiome and volatile-based mechanisms toward controlling *Meloidogyne*-based disease complexes. *Front. Microbiol.* 10, 1–15. doi:10.3389/fmicb.2019.01296.

Wu, Y., Yuan, J., E, Y., Raza, W., Shen, Q., and Huang, Q. (2015). Effects of volatile organic compounds from *Streptomyces albulus* NJZJSA2 on growth of two fungal pathogens. *J.*

Basic Microbiol. 55, 1104–1117. doi:10.1002/jobm.201400906.

Xing, M., Zheng, L., Deng, Y., Xu, D., Xi, P., Li, M., et al. (2018). Antifungal activity of natural volatile organic compounds against litchi downy blight pathogen *Peronophythora litchii*. *Molecules* 23, 358. doi:10.3390/molecules23020358.

Zhao, L. jing, Yang, X. nan, Li, X. ying, Mu, W., and Liu, F. (2011). Antifungal, insecticidal and herbicidal properties of volatile components from *Paenibacillus polymyxa* strain BMP-11. *Agric. Sci. China* 10, 728–736. doi:10.1016/S1671-2927(11)60056-4.

Zheng, L., Situ, J. jian, Zhu, Q. feng, Xi, P. gen, Zheng, Y., Liu, H. xia, et al. (2019). Identification of volatile organic compounds for the biocontrol of postharvest litchi fruit pathogen *Peronophythora litchii*. *Postharvest Biol. Technol.* 155, 37–46. doi:10.1016/j.postharvbio.2019.05.009.

Zhou, M., Li, P., Wu, S., Zhao, P., and Gao, H. (2019). *Bacillus subtilis* CF-3 volatile organic compounds inhibit *Monilinia fructicola* growth in peach fruit. *Front. Microbiol.* 10. doi:10.3389/fmicb.2019.01804.

Zhou, Y., Li, W., Zeng, J., and Shao, Y. (2018). Mechanisms of action of the yeast *Debaryomyces nepalensis* for control of the pathogen *Colletotrichum gloeosporioides* in mango fruit. *Biol. Control* 123, 111–119. doi:10.1016/j.biocontrol.2018.05.014.

Zou, C. S., Mo, M. H., Gu, Y. Q., Zhou, J. P., and Zhang, K. Q. (2007). Possible contributions of volatile-producing bacteria to soil fungistasis. *Soil Biol. Biochem.* 39, 2371–2379. doi:10.1016/j.soilbio.2007.04.009.
